# Deciphering colorectal cancer radioresistance and immune microrenvironment: unraveling the role of EIF5A through single-cell RNA sequencing and machine learning

**DOI:** 10.3389/fimmu.2024.1466226

**Published:** 2024-09-03

**Authors:** Yaqi Zhong, Xingte Chen, Shiji Wu, Huipeng Fang, Liang Hong, Lingdong Shao, Lei Wang, Junxin Wu

**Affiliations:** ^1^ Department of Radiation Oncology, Clinical Oncology School of Fujian Medical University Fujian Cancer Hospital, (Fujian Branch of Fudan University Shanghai Cancer Center), Fujian Cancer Hospital, Fuzhou, China; ^2^ Department of Hepatopancreatobiliary Surgery, Clinical Oncology School of Fujian Medical University, (Fujian Branch of Fudan University Shanghai Cancer Center), Fuzhou, China; ^3^ Department of Radiation Oncology, Jiangxi Clinical Research Center for Cancer, Jiangxi Cancer Hospital, The Second Affiliated Hospital of Nanchang Medical College, Nanchang, Jiangxi, China

**Keywords:** colorectal cancer, radiotherapy, eIF5A, cancer stem cells, tumor immune micorenvironment

## Abstract

**Background:**

Radiotherapy (RT) is a critical component of treatment for locally advanced rectal cancer (LARC), though patient response varies significantly. The variability in treatment outcomes is partly due to the resistance conferred by cancer stem cells (CSCs) and tumor immune microenvironment (TiME). This study investigates the role of EIF5A in radiotherapy response and its impact on the CSCs and TiME.

**Methods:**

Predictive models for preoperative radiotherapy (preRT) response were developed using machine learning, identifying EIF5A as a key gene associated with radioresistance. EIF5A expression was analyzed via bulk RNA-seq and single-cell RNA-seq (scRNA-seq). Functional assays and *in vivo* experiments validated EIF5A’s role in radioresistance and TiME modulation.

**Results:**

EIF5A was significantly upregulated in radioresistant colorectal cancer (CRC) tissues. EIF5A knockdown in CRC cell lines reduced cell viability, migration, and invasion after radiation, and increased radiation-induced apoptosis. Mechanistically, EIF5A promoted cancer stem cell (CSC) characteristics through the Hedgehog signaling pathway. Analysis of the TiME revealed that the radiation-resistant group had an immune-desert phenotype, characterized by low immune cell infiltration. *In vivo* experiments showed that EIF5A knockdown led to increased infiltration of CD8+ T cells and M1 macrophages, and decreased M2 macrophages and Tregs following radiation therapy, thereby enhancing the radiotherapy response.

**Conclusion:**

EIF5A contributes to CRC radioresistance by promoting CSC traits via the Hedgehog pathway and modulating the TiME to an immune-suppressive state. Targeting EIF5A could enhance radiation sensitivity and improve immune responses, offering a potential therapeutic strategy to optimize radiotherapy outcomes in CRC patients.

## 1 Introduction

Preoperative radiotherapy (preRT) serves as a fundamental component of neoadjuvant treatment for locally advanced rectal cancer (LARC), encompassing both long-course and short-course modalities (1). Despite its pivotal role, the efficacy of preRT varies, with complete response rates reported between 6% (2) and 28% (3), and even as high as 48.1% (4) in contexts involving intensified neoadjuvant systemic therapy. Nonetheless, around 40% of LARC patients exhibit resistance to preRT, underscoring the critical need to investigate the molecular bases of rectal radioresistance and to devise strategies that could enhance the treatment outcomes for these patients (5, 6).

Cancer stem cells (CSCs), characterized by their self-renewal capabilities, multidirectional differentiation potential, and heightened treatment resistance, are key contributors to radioresistance (7–9). Research indicates that CSCs may support radioresistance via several mechanisms: 1) efficient DNA damage repair, allowing them to survive post-radiation (10, 11); 2) a higher proportion of cells in the quiescent G0 phase compared to rapidly dividing tumor cells (12); 3) prevalence in hypoxic tumor regions which are less amenable to radiotherapy (13); 4) unique metabolic traits that bolster their radiation resistance (14); 5) lower levels of reactive oxygen species and higher expression of ROS scavengers, reducing radiation-induced damage (15). However, the specific role of CSCs in radioresistance among CRC patients remain poorly understood.

Growing evidence suggests that radiotherapy (RT)-induced regulation of the tumor immune microenvironment (TiME) is a double-edged sword, maintaining a delicate balance between immune activation and immunosuppression (16, 17). On one hand, RT effectively promotes local and systemic anti-tumor immunity:1) Pro-inflammatory mediators produced in the tumor post-RT enhance antigen presentation by dendritic cells (DCs) and activate natural killer (NK) cells, thereby inducing *in situ* immune regulation (18, 19); 2) RT can generate an *in situ* vaccination (ISV) effect, converting the patient’s own tumor into a nidus of enhanced antigen presentation, leading to a stronger immune response that can also target distant disease sites (i.e., abscopal effects) (20, 21). On the other hand, RT can negatively modulate tumor immunity: 1) The reduction of lymphocytes and NK cells following RT leads to immunosuppression (22); 2) RT-induced activation of TGF-β promotes the proliferation of myeloid-derived suppressor cells (MDSCs) and M2 macrophages, creating an immunosuppressive tumor microenvironment (23). Therefore, identifying the essential molecules responsible for modulating the TiME following radiation is crucial.

In this research, we developed 17 predictive models of radiation response using machine learning techniques and identified EIF5A as a principal gene associated with radioresistance, based on the Shapley value of each gene included. Subsequent experiments showed that EIF5A was upregulated in radioresistant CRC tissues, as confirmed by both bulk RNA-seq and single-cell RNA-seq analyses. In addition, downregulation of EIF5A was observed to reverse the radioresistant phenotype *in vitro*, with subsequent investigations indicating a potential role for EIF5A in promoting the transition of epithelial cells to stem cells via the Hedgehog signaling pathway. Compared with radioresistant samples, radiosensitive samples demonstrated a higher level of immune cell infiltration, with the low-EIF5A subgroup showing particularly higher infiltration levels. Furthermore, knockdown of EIF5A was shown to augment the effects of radiation *in vivo* and alter TiME. Collectively, these findings illuminate the novel functions of EIF5A in CRC and propose it as a potential target to mitigate radioresistance and remodel TiME in CRC patients.

## 2 Materials and methods

### 2.1 Gene expression data from public databases

Gene expression data and clinical information for CRC patients were obtained from the Gene Expression Omnibus (GEO, https://www.ncbi.nlm.nih.gov/geo/) and The Cancer Genome Atlas (TCGA) cohort (https://tcga-data.nci.nih.gov/tcga/). Immunohistochemical (IHC) data for the TCGA cohort were sourced from the Human Protein Atlas (HPA) database (https://www.proteinatlas.org/). Five bulk RNA-seq cohorts receiving preRT were included, namely GSE35452, GSE68204, GSE145037, and GSE150082, providing complete preRT response information categorized by tumor regression grade (TRG). TRG 0-1 denoted radiosensitive samples, while TRG 2-3 represented radioresistant samples (24). Additionally, GSE87211 comprised normal tissue samples with detailed overall survival (OS) and disease-free survival (DFS) data. The “ComBat” method within the “sva” R package was applied to mitigate batch effects across all cohorts. Three single-cell RNA-seq datasets (GSE132465, GSE166555, and GSE178318) of CRC were retrieved from the GEO database. **Supplementary Table S1** presents a summary of the baseline characteristics of the cohorts.

### 2.2 Construction of preRT response predicting model by machine learning

Seventeen machine learning algorithms were deployed in the GSE68204 cohort to formulate diagnostic models predicting the efficacy of preRT. These algorithms, including glmBoost, random forest (RF), Lasso, Ridge, Enet, SVM, multiNom, plsRglm, RDA, LDA, amdai, GBM, KNN, XGBoost, Stepglm, NaiveBayes, and LogisticR, were subsequently validated across three additional cohorts (GSE35452, GSE145037, and GSE150082), with model performance assessed via receiver operating characteristic (ROC) analysis.

Key radioresistant gene screening by SHapley Additive exPlanations (SHAP). SHAP was employed to interpret machine learning models by quantifying each feature’s contribution to the predictions (25). The “fastshap” R package and “shapviz” R package were utilized to compute the mean Shapley value of each gene across the four cohorts within each model (26). Subsequently, genes demonstrating significant impacts on the models were filtered for further analysis.

### 2.3 Single-cell RNA sequencing data quality control and processing

The Seurat single-cell standard workflow was implemented for analysis and integration (27). Cells with <250 or >5000 measured genes and those with >25% mitochondrial contamination were filtered out. A total of 106,634 cells were selected for processing, comprising 65,412 cells from 52 tumor tissue samples and 41,222 cells from 29 normal tissue samples. The merged objects were normalized and the 3,000 most variable genes were identified. Principal component analysis (PCA) was applied to scale gene expression and reduce dimensionality. Batch effects were corrected, and objects were integrated using the “Harmony” R package. T-distributed stochastic neighbor embedding (t-SNE) was employed for visualization.

### 2.4 scRNA-seq cell type annotation and analysis

Distinct cell types were labeled using canonical marker genes based on classical immune cell markers sourced from CellMarker 2.0. Dotplots visualized gene expression for each cluster, and the “scissor” R package identified phenotype-driven single-cell subpopulations. Cell-cell communication was analyzed using the “CommPath” R package, and signaling pathway networks within clusters were depicted via heatmaps. Cellular trajectories were inferred using the “Monocle2” R package, facilitating pseudotime analysis to describe high-dimensional expression values.

### 2.5 Functional analysis

Gene set enrichment analysis (GSEA) was employed to assess biological functions and signaling pathways in bulk RNA-seq and scRNA-seq datasets. Stemness, indicating the differentiation level and potential of cells, was evaluated using the One-Class Logistic Regression (OCLR) algorithm (28).

### 2.6 Cell culture and animals

MC38 cells (murine colorectal cancer cells) and HCT116 cells (human colorectal cancer cells) were obtained from the national cell line resource infrastructure of China and cultured in RPMI-1640 medium supplemented with 10% fetal bovine serum and 1% penicillin-streptomycin.

In order to conduct *in vivo* studies, female C57BL/6 mice aged 6-8 weeks were procured from the Shanghai Wushi Experimental Animal Center in Shanghai, China. Approval for all animal studies was obtained from the Institutional Animal Care and Use Committee at Fujian Medical University.

### 2.7 Transfection and irradiation

shRNA and siRNA targeting EIF5A, control scrambled shRNA and siRNA, and lentiviral vector were procured from ZolGene. Transfection was performed in accordance with manufacturer’s protocols. Scrambled shRNA and siRNA were used as control. Following validation was conducted through the utilization of quantitative reverse transcription PCR (qRT-PCR) and western blotting. Both cell lines and mice in the irradiation (IR) group received 6 Gy of X-ray radiation using a Varian Truebeam linear accelerator.

### 2.8 Western blotting, qRT-PCR and immunofluorescence assay

Total protein collection, RNA extraction and reverse transcription were conducted according to the methods described in a previous study (29). EIF5A expression was normalized to β-actin. Western blot and qRT-PCR analysis was performed in triplicate, and the average value was calculated using the gray value and 2^-ΔΔCt^ method. The primer sequences for EIF5A and β-actin are listed in **Supplementary Table S2**. Samples of tumor tissues obtained from mice were collected for immunofluorescence analysis according to the manufacturers protocol. Primary antibodies information is listed in **Supplementary Table S3**.

### 2.9 Wound healing assay

Cells were inoculated in 6-well plates, and wounds were scraped with 200 μL pipettes when the cells reached 90% confluency. The cells positions were then photographed and recorded at 0, 24, and 48h. Lastly, the migration rate of each group was calculated using the following formula: (initial area-final area)/initial area.

### 2.10 Transwell migration and invasion assay

Cells suspension were seeded in a 24-well perforated transwell chamber with pores size of 8.0µm (Corning Costar, USA) with/without pre-coated Matrigel. The culture medium containing 10% FBS was poured into the lower chamber. After 24 hours of incubation, the membranes were collected, stained with crystal violet solution and removing cells that cannot migrate or invade through pores. Migrating and invading cells were counted and photographed under a microscope in 5 different fields.

### 2.11 Cell viability and rescue experiment

The viability of cells was assessed through Cell Count Kit-8 (CCK-8), Calcein-AM/Propidium Iodide (PI) staining and 5-Ethynyl-2-Deoxyuridine (EdU) assay, respectively, according to the manufacturer’s instructions. Cells were inoculated into 96-well culture plates and irradiation group was given 6Gy irradiation. All groups’ cells were analyzed optical density (OD) rate at 0, 24, 48, 72 and 96h. The fluorescence images were acquired by using a fluorescence microscope (Leica DM2500). Image Pro advanced software was used to analyze the mean fluorescence intensity (MFI). SAG (HY-12848, MedChemExpress, USA), as the agonist of Hedgehog pathway, was used for the rescue experiment of Hedgehog pathway.

### 2.12 Colony formation assay

Cells were inoculated into 6-well culture plates for 8 to 10 days. The irradiation group was given 6Gy irradiation. During the culture period, the medium containing 10% FBS was changed half times every 2-3 days. Subsequent crystal violet staining and colonies counting were performed when single visible clone was observed in the negative control (NC) group.

### 2.13 Reactive oxygen species assay

Cells were inoculated into 96-well culture plates and irradiation group was given 6Gy irradiation. All groups’ cells were incubated with the peroxide-sensitive fluorescent probe DCFH-DA (1mM) for 2h in the dark, before imaging. The fluorescence images were acquired by using a fluorescence microscope (Leica DM2500) and image Pro advanced software was used to analyze the MFI.

### 2.14 Cell apoptosis

Cells were inoculated into 24-well culture plates and irradiation group was given 6Gy irradiation. All groups’ cells were analyzed with an annexin V-APC/PI double staining apoptosis detection kit (KeyGen BioTech) according to the manufacturer’s instructions, respectively. And then the apoptosis of cells detected by flow cytometry (FCM).

### 2.15 Sphere formation assay

Cells were planted in low-adsorption 6-well plates and cultured with DMEM/F12 medium for 8 to 10 days, which contained 20g/ml epidermal growth factor (EGF), 10ng/ml basic fibroblast growth factor (bFGF) and 2% B27. Then the spheres were collected and photographed, and their sizes were measured by the Image pro plus software.

### 2.16 Patients and tissue samples

A total of 80 frozen colorectal cancer samples (including 15 paired normal tissue samples) undergoing both nCRT and radical surgery were received at Fujian Provincial Cancer Hospital (FJCH) between March 2016 and March 2022. Two consultant pathologists assigned preRT response assessed by TRG according to the American Joint Committee on Cancer (AJCC) criteria. This study was approved by the Ethics Committee of FJCH (K2023-181-01).

### 2.17 IHC

IHC staining were performed using the antibodies against EIF5A (1: 5000, ab32443, Abcam) as described previously. All immunostaining images were evaluated blindly based on the histochemical score. As reported previously, the H-score was determined by multiplying the staining intensity score (negative = 0, weak = 1, moderate = 2, strong = 3) with the positive rate score (negative=0, 1-25% = 1, 26-50% = 2, 51-75% = 3, 76-100% = 4).

### 2.18 Statistical analyses

All analyses were conducted using R (version 4.0.3). Normality was assessed using the Shapiro-Wilk test, with Student’s t-test or Wilcoxon test employed for group comparisons depending on data distribution. Parametric and nonparametric tests were used for multiple group comparisons. Correlation analyses were conducted using Spareman and distance correlation coefficients. P<0.05 indicates statistical significance.

## 3 Results

### 3.1 Machine learning-based screening of key genes for radioresistance in CRC

**Figure 1:**
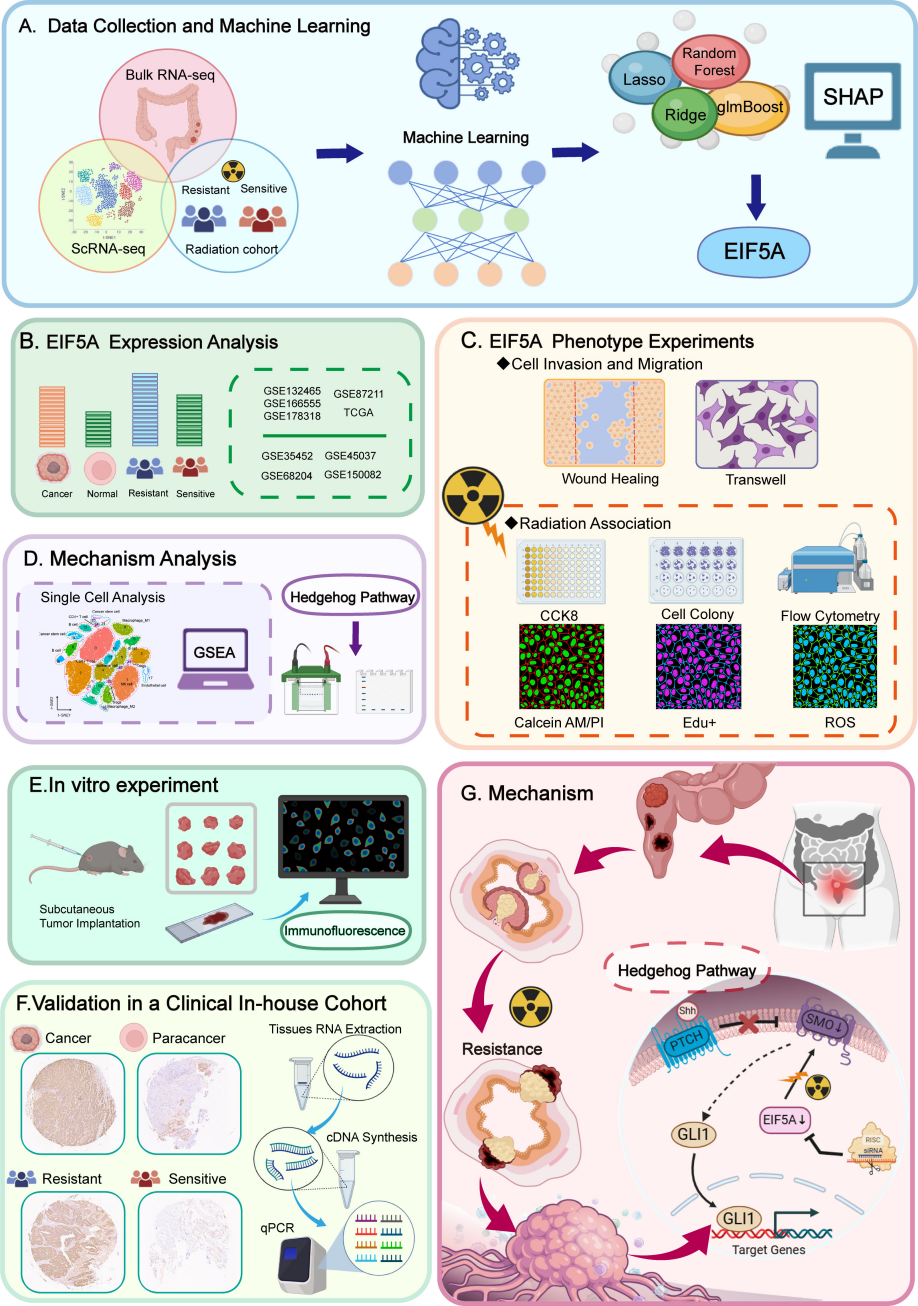
Flowchart of the present study.

**Figure 2:**
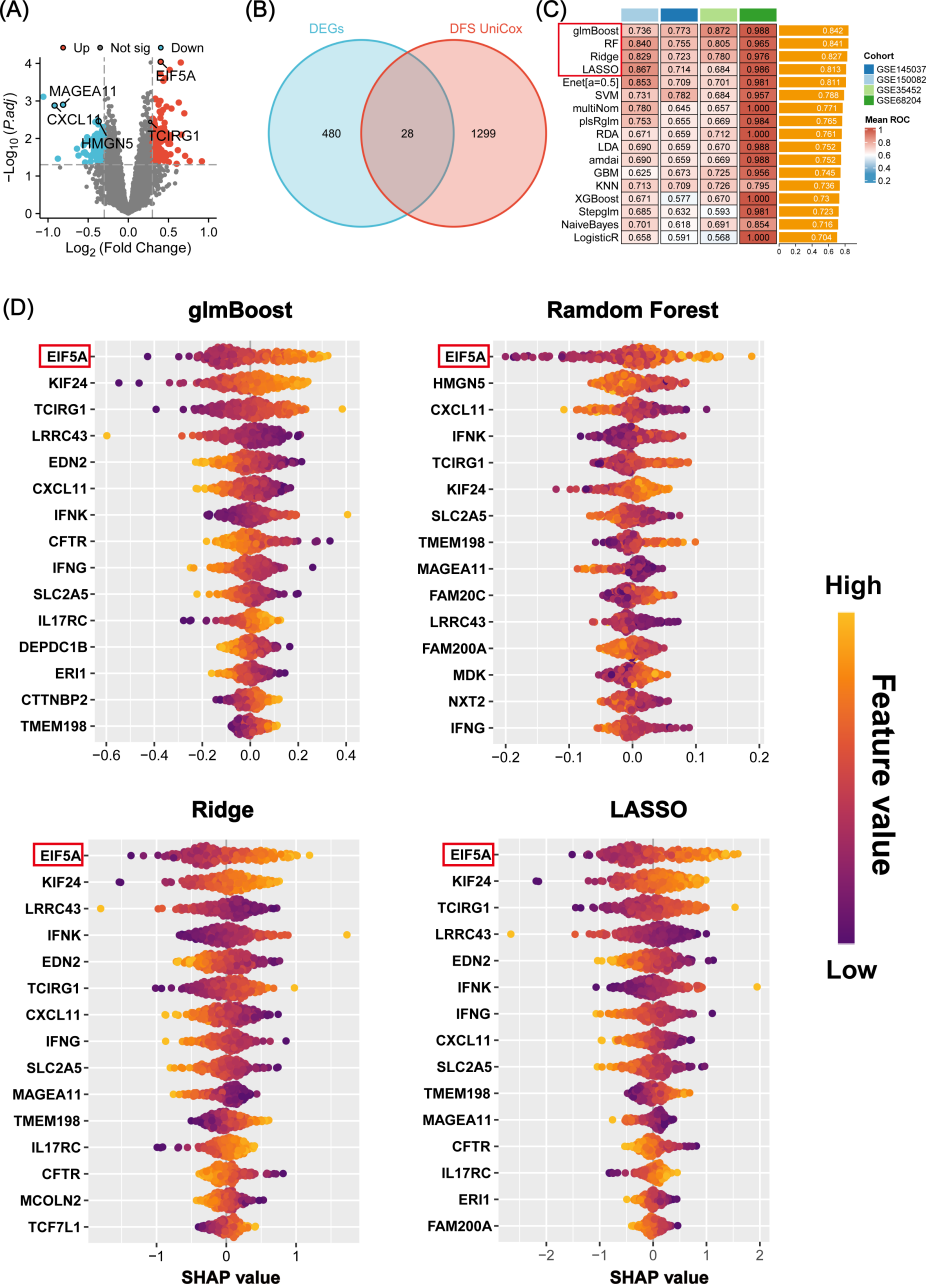
Machine learning-based screening of key genes for radiation sensitivity. **(A)** Volcano plot showing the DEGs between the radiosensitive and radioresistant groups in meta-cohort. **(B)** The 28 candidate genes were identified via venn diagram. **(C)** A total of 17 prediction models and the ROC of each model across all cohorts. **(D)** Beeswarm plot of SHAP values (shows how and how much each gene influences the predictions) in four machine learning models across all cohorts. ROC, Receiver operating characteristic; SHAP, SHapley Additive explanation.

The study’s overall concept is depicted in the flow chart of **Figure 1**. Initially, 508 differentially expressed genes (DEGs) were identified between radiosensitive tissues and radioresistant tumors in the meta-cohort (**Supplementary Table S4**; **Figure 2A**), while 1,327 genes were associated with patient DFS in the GSE87211 cohort (**Supplementary Table S5**). Subsequently, 28 intersection genes between DEGs and prognosis-related genes were considered as candidate genes to establish response predicting models (**Figure 2B**). Seventeen models of machine learning were then developed in the GSE68204 cohort and validated across GSE150082, GSE145037, and GSE35452, achieving mean area under curve (AUC) of the ROC curve ranging from 0.704 to 0.842 (**Figure 2C**). The top four performing models, based on mean AUC, were glmBoost, RF, Ridge, and LASSO. Utilizing the SHAP algorithm, SHAP summary plot of shows how and how much each gene influences the prediction in the four machine learning models (**Figure 2D**). Notably, EIF5A was identified as the predominant feature that exerted the greatest influence on all four machine learning models. Consequently, EIF5A was designated as the key gene associated with radioresistance in rectal cancer.

### 3.2 Multiomics validation of radiation resistance by EIF5A

**Figure 3:**
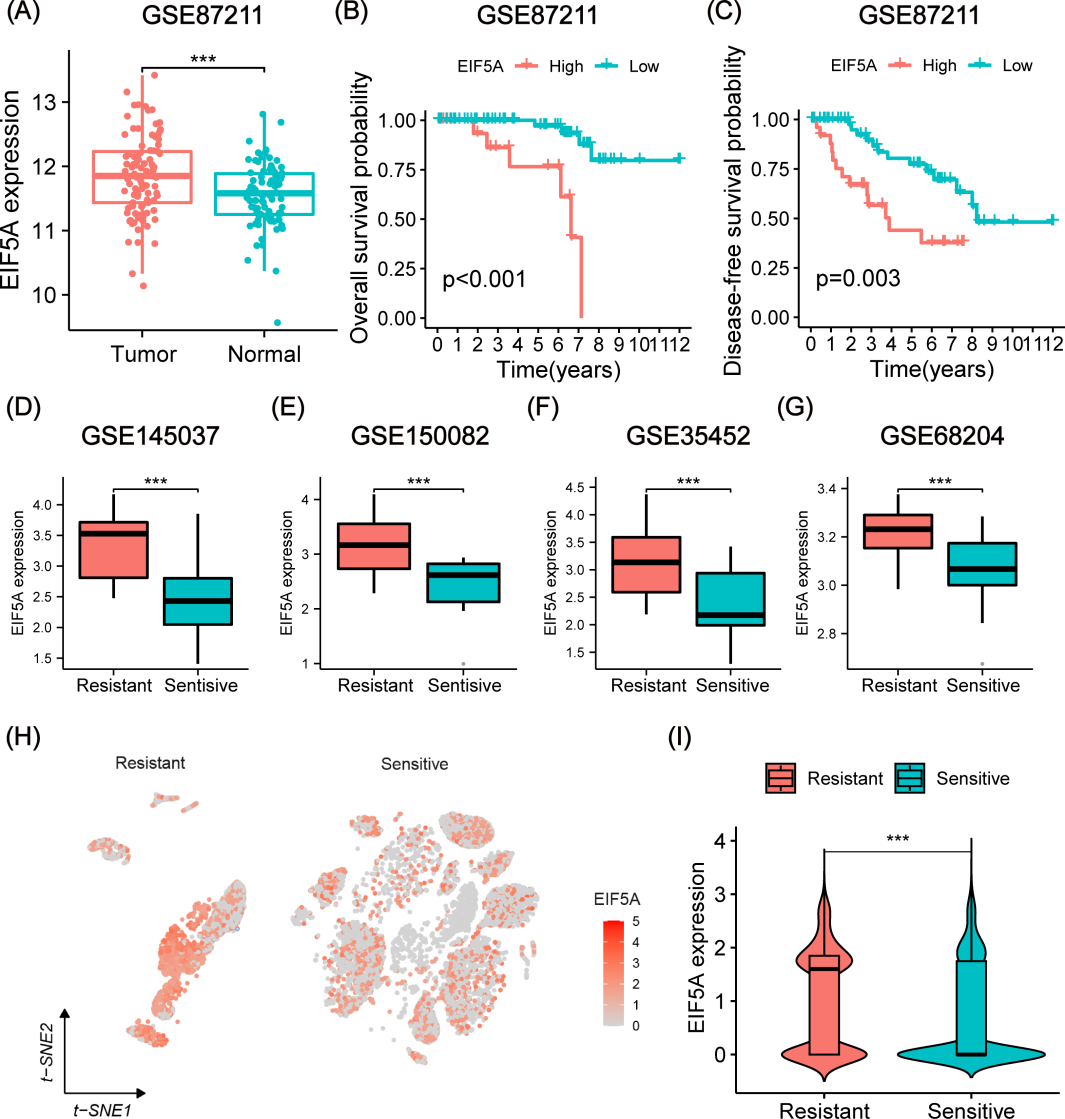
Prognosis and differential expression of EIF5A in cohorts receiving preRT. **(A)** Boxplot showing differential expression of EIF5A between normal/peritumor and tumor in the GSE87211 cohort. **(B, C)** Kaplan-Meier curves of OS and DFS with different EIF5A expression groups in the GSE87211 cohort. **(D-G)** Boxplots of the difference in EIF5A expression between the radioresistant and radiosensitive groups in the GSE145037, GSE150082, GSE35452, and GSE68204 cohort. **(H-I)** t-SNE plot and violin plot showing the distribution of EIF5A expression between radioresistant and radiosensitive cells. OS, Overall survival; DFS, Disease free survival; t-SNE, Stochastic neighbor embedding; ***: P < 0.001.

As anticipated, EIF5A exhibited upregulation in both colon and rectal cancer within the TCGA cohort, as confirmed by GEPIA analysis (**Supplementary Figure S1A**). This finding was further validated using IHC and scRNA-seq analyses (**Supplementary Figures S1B–D**). Analysis of the GSE87211 cohort revealed overexpression of EIF5A in tumor tissues compared to normal tissues (P<0.001, **Figure 3A**), with higher EIF5A expression correlating with worse OS (P<0.001, **Figure 3B**) and DFS (P=0.003, **Figure 3C**). Similarly, EIF5A expression was significantly elevated in radioresistant samples compared to radiosensitive ones across multiple cohorts of GSE145037, GSE150082, GSE35452, and GSE68204 (P<0.001, **Figures 3D–G**). In the scRNA-seq meta-cohort, 106,634 cells from 81 samples (including 52 tumor tissues and 29 normal tissues) were collected for further processing, and the t-SNE plot were depicted in **Figure 3H**. Similarly, the EIF5A expression in the radioresistant samples was also significantly higher than those in the radiosensitive samples based on “scissor” algorithm(P<0.001, **Figure 3I**).

### 3.3 *In vitro* validation of radioresistance by EIF5A

**Figure 4:**
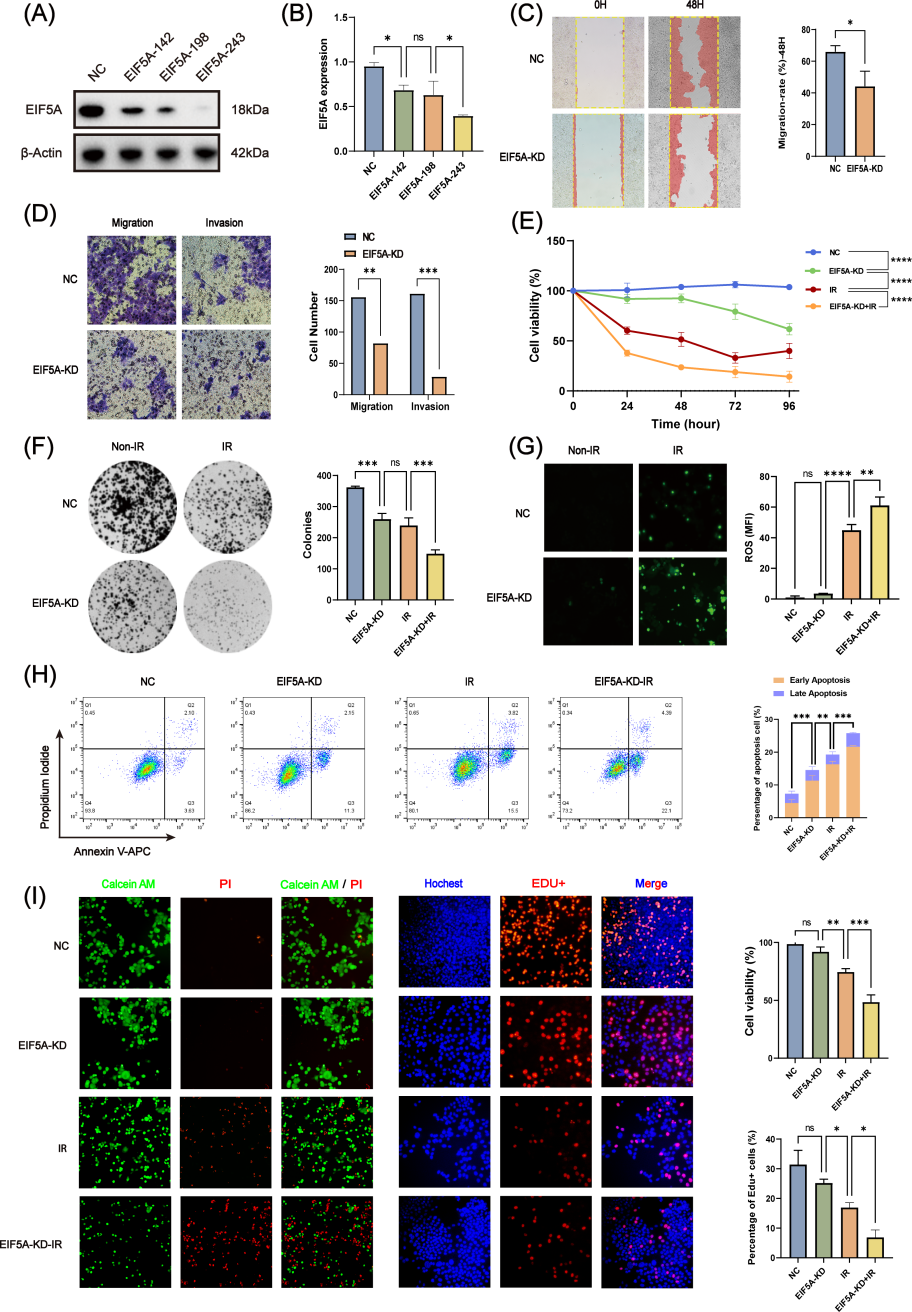
*In vitro* validation of radiation resistance by EIF5A. **(A, B)** The transfection efficiency of siEIF5A in HCT116 cell line, which was examined by western blot and qRT-PCR. **(C)** Representative wound healing images of migration after knocking down EIF5A. **(D)** Representative transwell images of migration and invasion after knocking down EIF5A. **(E)** CCK-8 assay to analyze the effect of radiation on cell proliferation of the irradiated and non-irradiated groups after knocking down EIF5A. **(F)** Representative colony formation images of the irradiated and non-irradiated groups after knocking down EIF5A. **(G)** Representative ROS production images of the irradiated and non-irradiated groups after knocking down EIF5A. **(H)** Flow cytometry assay to analyze the cell apoptosis changes in irradiated and non-irradiated groups after knocking down EIF5A. **(I)** Calcein-AM/PI staining assay and EdU incorporation assay to detected the cell death and proliferation in irradiated and non-irradiated groups after knocking down EIF5A. qRT-PCR, Quantitative reverse transcription polymerase chain reaction; CCK-8, Cell counting kit-8; ROS, Reactive oxygen species; EdU, 5-Ethynyl-2’-deoxyuridine; NC, Negative control; KD, Knock down; IR, Irradiation; ns, Non-statistics significance;*: P < 0.05; **: P < 0.01; ***: P < 0.001.

To further verify whether EIF5A could result in the radioresistance of CRC, we knock down the EIF5A in the HCT116 cell line by three different siRNAs. Through western blot and qRT-PCR (**Figures 4A, B**, **Supplementary Figure S2**), we selected EIF5A-243 as the candidate siRNA which was used in the following experiment. First, knock down of EIF5A sharply was found to decrease the migration and invasion of HCT116 cells, compared with NC group of HCT116 cells (all P<0.05, **Figures 4C, D**). Upon 6 Gy X ray, the cell viability of EIF5A-KD cells was decreased sharply to 9.6% compared with NC group (27.8%, P<0.001, **Figure 4E**), as well as impaired clones (149.0 vs. 239.3, P<0.001, **Figure 4F**). From the other hand, the ROS production was significantly increased in the EIF5A-KD cells upon 6 Gy radiation compared with NC group (61.18 vs. 44.9, MFI, P<0.001, **Figure 4G**). FCM results revealed that 6 Gy irradiation resulted in increased apoptosis in EIF5A-KD group compared with NC group, both in early apoptosis and total apoptosis (35.5% vs. 50.0%, P<0.001; 73.4% vs. 89.1%, P<0.001; **Figure 4H**). Calcein-AM/PI staining assay showed that the cell viability was decreased from 74.5% (irradiation group) to 48.5% (irradiation+EIF5A-KD group, P<0.001, **Figure 4I**), which was also verified by EdU staining (P<0.05, **Figure 4I**). Taken together, knock down of EIF5A could reverse the radioresistance in CRC.

### 3.4 The underlying mechanism of radioresistance by EIF5A

**Figure 5:**
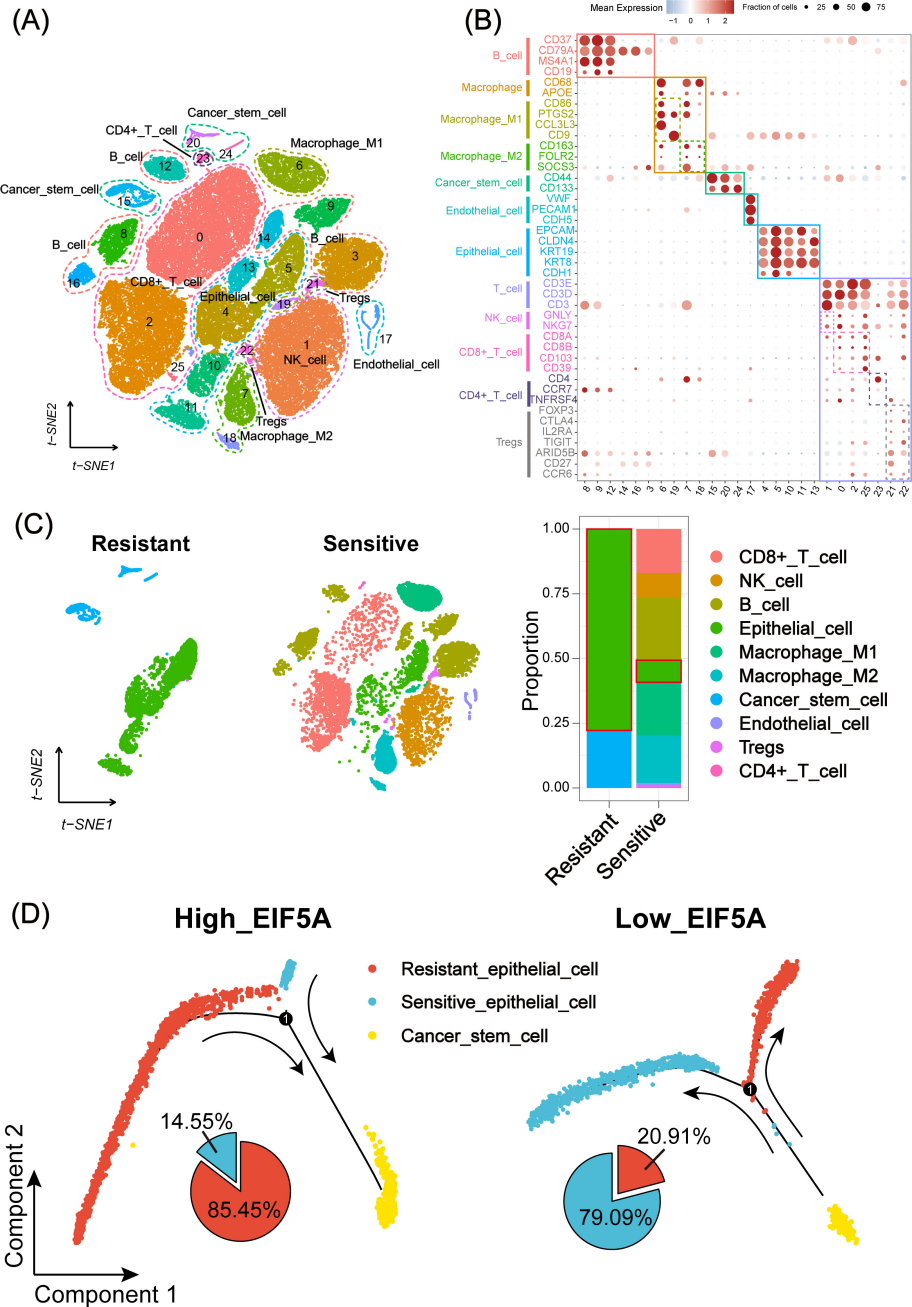
Landscape and potential mechanism of radiation resistance via scRNA-seq. **(A)** The t-SNE plot of 26 cell clusters from the multicellular ecosystem of three CRC scRNA-seq cohorts. **(B)** Dotplot showing the percentage of expressed cells and average expression levels of canonical marker genes of major cell types in 26 cell clusters. **(C)** t-SNE plot and bar plots indicating the landscape and proportion of major cell lineages between radioresistant and radiosensitive cells. **(D)** Pseudotime analysis of epithelial cell and cancer stem cell in high- and low-EIF5A expression groups. scRNA-seq, Single-cell RNA sequencing; t-SNE, Stochastic neighbor embedding; CRC, Colorectal cancer.

As is known to all, CSCs plays an important role in the radioresistance (22). The tumor samples in three CRC scRNA-seq cohorts were collected to explore the potential mechanism of EIF5A in the radioresistance. Totally, 65,412 cells were identified as 26 cell clusters (**Figure 5A**), which were annotated with canonical marker genes of major cell types (**Figure 5B**). Similarly, cells were classified as radioresistant cells and radiosensitive cells according to “scissor” algorithm. Interestingly, the proportion of CSCs in the resistant cells was greatly higher than that in the sensitive ones (**Figure 5C**, P<0.05). Pseudotime analysis revealed a distinct tendency that more epithelial cells would transform into CSCs in high-EIF5A cells, regardless of radioresistant or radiosensitive, compared with low-EIF5A ones (**Figure 5D**, **Supplementary Figure S3**). Hence, we supposed that EIF5A might promote the transformation of epithelial cell into CSCs and result in radioresistance. Cellular communication were then conducted to explore the potential interaction counts and interaction intensity of ligands and receptors among cell subpopulations using “CommPath” method (**Supplementary Figures S4A, B**). The potential pathways activated in cell subpopulations were identified from via GSEA, among which Hedgehog signaling pathway was activated both in CSC-resistant cells and epithelial-resistant cells (**Supplementary Figure S4C**).

**Figure 6:**
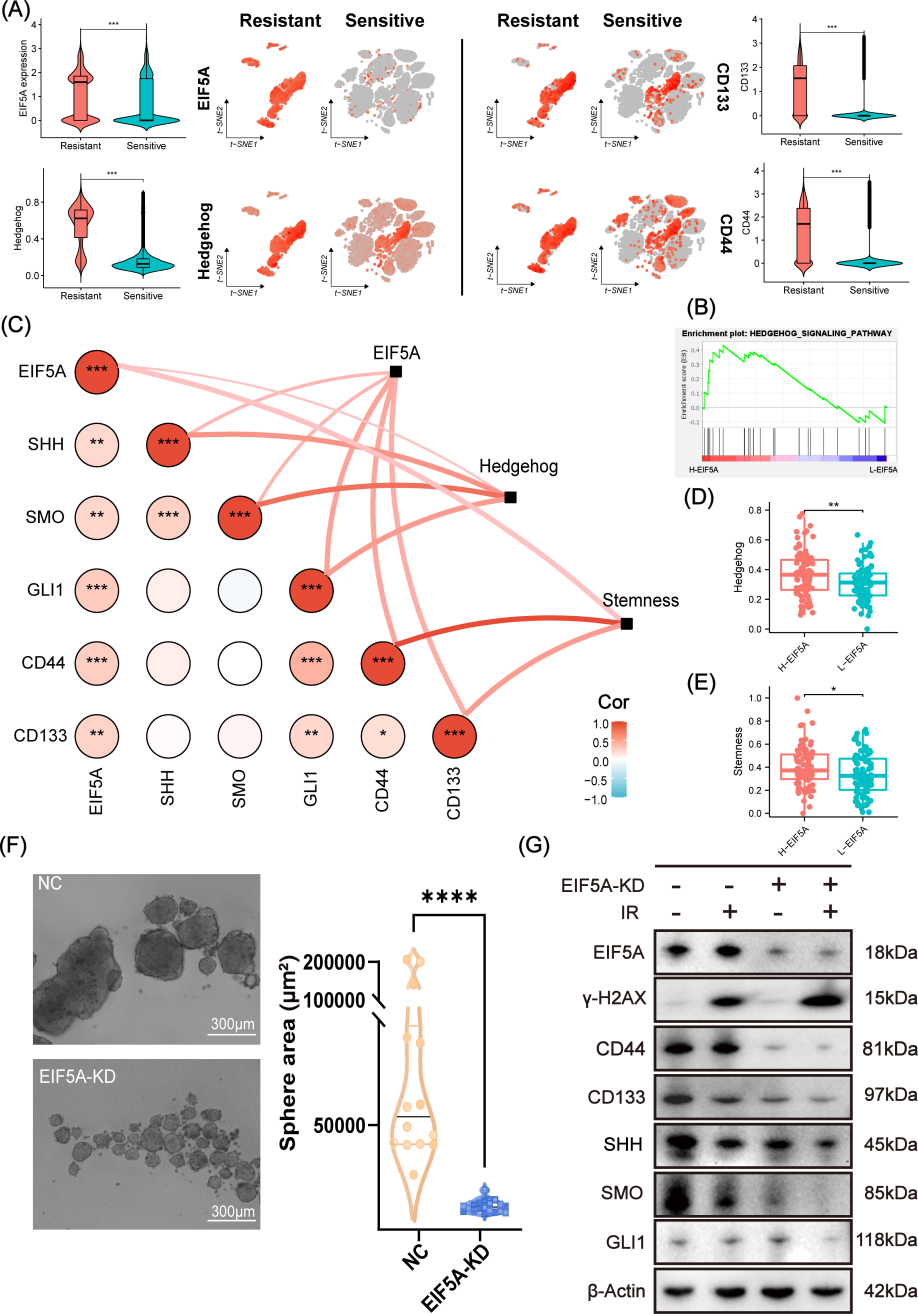
Analysis and validation of EIF5A mediating radiation resistance via Hedgehog signaling pathway. **(A)** t-SNE plots and violin plots illustrating the distribution of the expression of EIF5A, CD44, CD133, and the ssGSEA score of Hedgehog in radioresistant and radiosensitive cells. **(B)** GSEA result based on the DEGs between the high- and low-EIF5A group in meta-cohort. **(C)** The correlations between EIF5A expression and the Hedgehog signaling pathway & its key genes (e.g. SHH, SMO, and GLI1), as well as EIF5A expression and stemness & its key genes (e.g. CD133 and CD44), were being investigated by correlation network and heat map. **(D, E)** Boxplots indicating the distribution of Hedgehog signaling pathway and stemness between high- and low-EIF5A group. **(F)** Representative tumor sphere formation images in HCT116 cell line after knocking down EIF5A. **(G)** Representative western blot images showing the expression levels of EIF5A, γ-H2AX, and the key genes of stemness (e.g. CD133 and CD44) and Hedgehog signaling pathway (e.g. SHH, SMO, and GLI1) in the irradiated and non-irradiated groups after knocking down EIF5A. t-SNE, Stochastic neighbor embedding; GSEA, Gene set enrichment analysis; ssGSEA, Single sample gene set enrichment analysis; NC, Negative control; KD, Knock down; IR, Irradiation; ns, Non-statistics significance;*: P < 0.05; **: P < 0.01; ***: P < 0.001; ****: P < 0.0001.

As expected, EIF5A was highly expressed in radioresistant cells compared with radiosensitive cells, as well as up-regulated Hedgehog signaling pathway, CD133 and CD44 (all P<0.001, **Figure 6A**). GSEA revealed that Hedgehog signaling pathway was enriched in the high-EIF5A group compared with the low-EIF5A group in meta-cohort (**Figure 6B**). Further, correlation analysis revealed significantly positive correlation between EIF5A expression and the Hedgehog signaling pathway including its key genes of SHH, SMO, and GLI1 (all R>0 and P<0.05, **Supplementary Figures S5A–D**), and similar correlations were observed between EIF5A expression and stemness including its key genes of CD133 and CD44 (all R>0 and P<0.05, **Supplementary Figures S5E–G**), both of which were summarized in **Figure 6C**. As expected, Hedgehog signaling pathway score in the high-EIF5A group was significantly higher than that in the low-EIF5A group (P<0.01, **Figure 6D**), as well as elevated stemness score (P<0.05, **Figure 6E**). Furthermore, knock down of EIF5A was found to sharply weaken the sphere ability of HCT116 cells compared with the NC group (69,932.2μm2 vs. 6,907.2 μm2, P<0.001, **Figure 6F**). In addition, upon radiation, knock down of EIF5A decreased the expression of GLI1, SMO, SHH, CD133 and CD44, and ultimately resulted in increased γ-H2AX (A DNA double-strand damage marker used to reflect radiation response) (**Figure 6G**, **Supplementary Figure S6**), (23). Subsequent rescue experiments demonstrated a significant reduction in cell viability of irradiated EIF5A-KD CRC cells, decreasing from 37.89% to 55.76% following the application of SAG (P<0.05, **Supplementary Figure S7**). This finding was corroborated in the EdU assay (P<0.05, **Supplementary Figure S8**). Taken together, we concluded that EIF5A might promote the abundance of CSC via Hedgehog signaling pathway and result in radioresistance of CRC.

### 3.5 Landscape of the radiation-induced TiME modified by EIF5A

**Figure 7:**
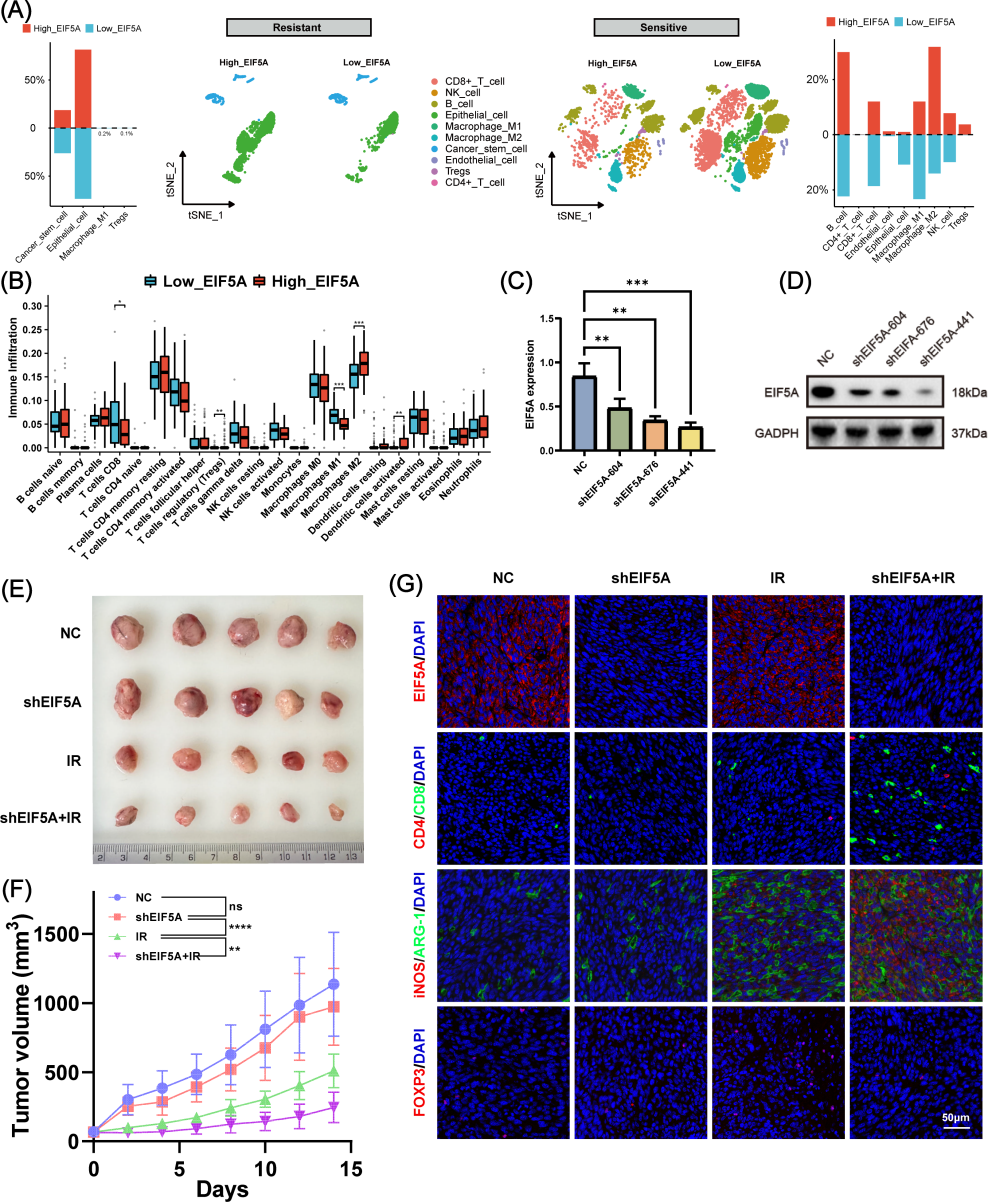
The role of EIF5A in the tumor immune microenvironment induced by radiation. **(A)** Disparities in cellular subpopulations between high- and low-EIF5A expression cells within the radioresistant and radiosensitive groups. **(B)** Differences in immune cell infiltration between high- and low-EIF5A groups in CRC patients who received radiation. **(C, D)** qRT-PCR and WB was used to screen knockdown efficiency of EIF5A in the shEIF5A-604, shEIF5A-676, and shEIF5A-441. **(E, F)** The tumor size and tumor volume curve of C57BL/6 mice in different groups (NC, shEIF5A, IR, and shEIF5A+IR group) at 14 day. **(G)** Typical immunofluorescence images of EIF5A (red), Cd4 (red), Cd8 (green), iNos (red), Arg-1 (green), and Foxp3 in different groups. Scale bar, 50μm. CRC, colorectal cancer; qRT-PCR, Quantitative reverse transcription polymerase chain reaction; WB, Western blot; NC, Negative control; IR, Irradiation; ns, Non-statistics significance;*: P < 0.05; **: P < 0.01; ***: P < 0.001.

Radiation resistance is often associated with an immune-desert TiME (30). To investigate this, we analyzed the TiME profile of the radiation-resistant group and explored the relationship between immune infiltration and EIF5A expression. **Figure 7A** shows that the radiation-resistant group had an immune-desert TiME, compared with the radiation-sensitive cohort. But among the radiation-sensitive cohort, there was an immune-infiltrating TiME characterized by a notable increase in CD8+ T cells, M1 macrophages, and NK cells in the low-EIF5A subgroup; while the proportion of M2 macrophages and Tregs was significantly reduced in the high-EIF5A subgroup (**Figure 7A**). Similar results were observed in the bulk RNA-seq cohort that received radiation (all P<0.05, **Figure 7B**).

To further investigate the combined effects of EIF5A and radiation on TiME, we used shRNA to mediate stable EIF5A knockdown in MC38 cells, verified by RT-qPCR and Western blot (**Figures 7C, D**, **Supplementary Figure S9**). These cells were then implanted into the right hind limb of mice to establish a subcutaneous tumor model. **Figures 7E, F** shows that knockdown of EIF5A alone did not yield statistically significant changes in tumor volume. However, by day 14, the mean tumor volume in the shEIF5A+IR group exhibited a significant reduction compared to the other experimental groups (both P<0.01, **Figures 7E, F**). Immunofluorescence analysis indicated an increase in CD8+ T cells and M1 macrophages, and a decrease in M2 macrophages and Tregs in the shEIF5A+IR group compared to the NC, shEIF5A, and IR groups (**Figure 7G**). These results suggest that EIF5A knockdown may modulate the radiation-induced suppressive TiME and enhance positive immune responses to radiation, warranting further comprehensive investigations.

### 3.6 Clinical application of EIF5A in an in-house cohort

**Figure 8:**
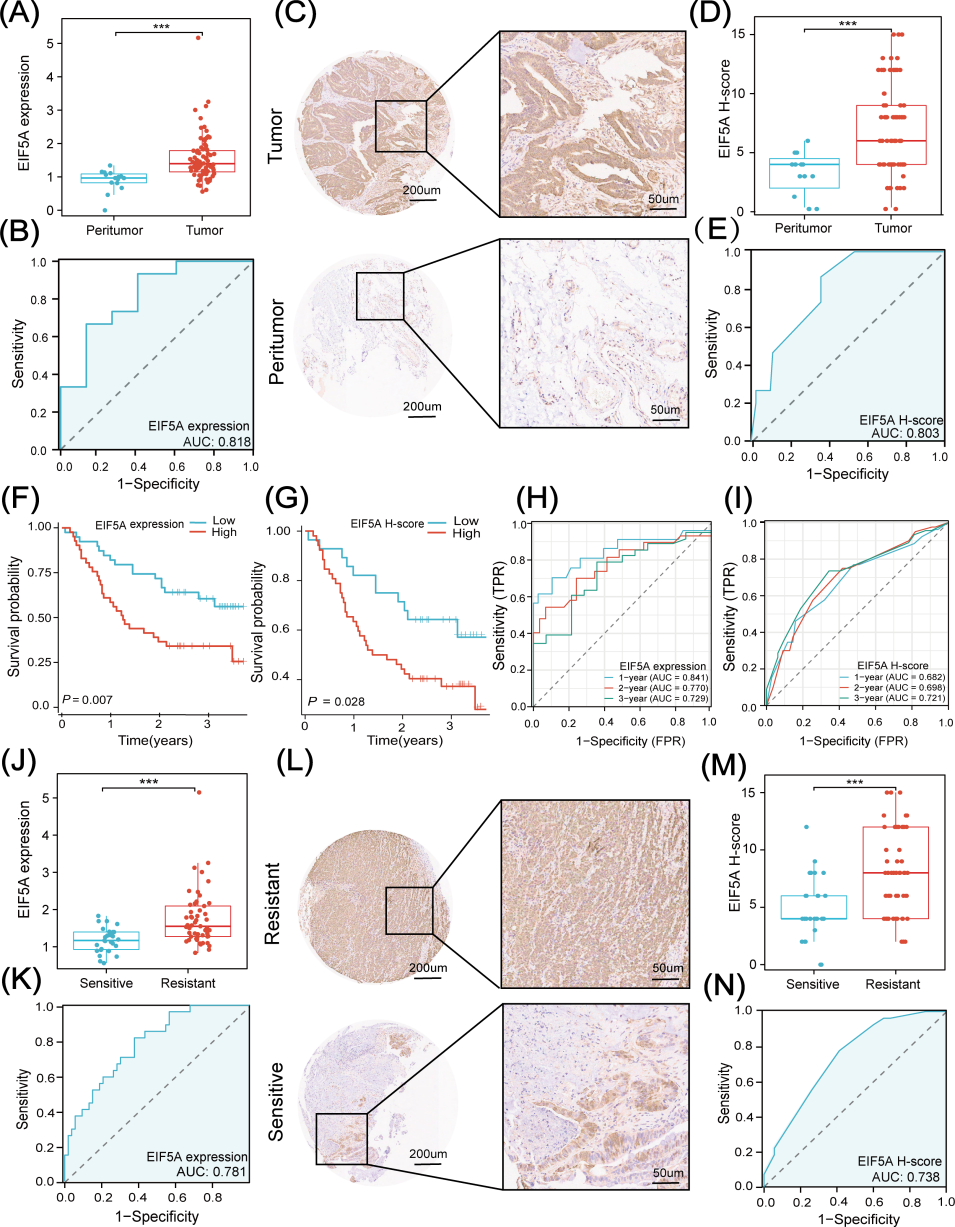
Clinical application of EIF5A in in-house cohort. **(A)** Boxplot shows the distribution of EIF5A expression between tumor and peritumor samples. **(B)** Diagnostic ROC curve analysis for predicting CRC. **(C, D)** IHC images and EIF5A H-score based on IHC images shows the distribution of EIF5A expression between tumor and peritumor samples. **(E)** Diagnostic ROC curve of EIF5A H-score analysis for predicting CRC. **(F, G)** Kaplan-Meier curves of OS with high- and low-EIF5A expression & H-score groups. **(H, I)** Time-dependent ROC curve of EIF5A expression & H-score analysis for predicting OS at 1-, 2-, and 3-years. **(J)** Boxplot shows the distribution of EIF5A expression between radiation resistant and sensitive samples. **(K)** Diagnostic ROC curve of EIF5A expression analysis for predicting radiotherapy sensitivity. **(L, M)** IHC images and EIF5A H-score based on IHC images showing the distribution of EIF5A expression between radioresistant and radiosensitive samples. **(N)** Diagnostic ROC curve of EIF5A H-score analysis for predicting radiotherapy sensitivity. ROC, Receiver operating characteristic; IHC, Immunohistochemistry; OS, Overall survival; CRC, Colorectal cancer; ***, P < 0.001.

In the subsequent analysis, we explored the clinical application of EIF5A in an in-house cohort of 80 CRC patients treated with preRT using qRT-PCR and IHC. First, we analyzed clinicopathological data in the high- and low-EIF5A expression/H-score groups to see if there was any clinicopathological difference. As was depicted in **Supplementary Figures S10A–D**, EIF5A expression in the subgroups of female and Grade 3-4 were significantly higher than their counterparts (both P<0.05), and similar findings were observed in terms of H-score (**Supplementary Figures S10E–H**). As expected, the EIF5A expression in the tumor was significantly higher in the tumors than that in the peritumor tissues via qRT-PCR (P<0.001, **Figure 8A**), which could also predict CRC with the AUC of 0.818 (**Figure 8B**). Similar findings were observed using H-score (P<0.001, AUC of 0.803, **Figures 8C–E**).

Besides, EIF5A expression was significantly associated with CRC in logistic regression analysis (both P<0.001, **Supplementary Figure S11A**). In addition, patients with high-expression of EIF5A had worse OS compared with those with low-expression of EIF5A, regardless of qRT-PCR (P<0.001, **Figure 8F**) or IHC (P=0.028, **Figure 8G**). And both of EIF5A expression and H-score were the prognostic factors of OS using univariate and multivariate Cox regression analysis (both P<0.001, **Supplementary Figure S11B**). The 1-, 2-, 3-year AUC of EIF5A expression in predicting the OS of CRC patients were 0.841, 0.770 and 0.729, respectively (**Figure 8H**); while, the corresponding AUC of H-score in predicting the OS of CRC patients were 0.682, 0.698 and 0.772, respectively (**Figure 8I**).

Similarly, tumors were divided into radioresistant and radiosensitive ones according to TRG as mentioned above. The EIF5A expression in the radioresistant samples was significantly higher than that in the radiosensitive samples using qRT-PCR (P<0.001, **Figure 8J**), which could predict the response to nCRT with the AUC of 0.781 (**Figure 8K**). Besides, the H-score in the resistant samples was significantly higher than that in the sensitive samples via IHC (P<0.001, **Figures 8L, M**) with the AUC of 0.738 (**Figure 8N**). Logistic regression analysis revealed that both EIF5A expression and H-score were significantly associated with the preRT response in CRC (both P<0.001, **Supplementary Figure S11C**).

## 4 Discussion

Radiotherapy stands as a cornerstone of local treatment for CRC, yet identifying patients who stand to gain the most from it remains a formidable challenge (31). While factors such as tumor stage (32), location (33), and genetic profile (34) traditionally influence treatment decisions, they often fall short as definitive predictors of treatment response. In this study, we pioneered the construction of preRT response prediction models using machine learning, achieving an impressive ROC of 0.988. Notably, among the top four predicting models, EIF5A emerged as the key gene associated with radioresistance, boasting the highest Shapley value. Subsequent investigations unveiled EIF5A’s potential mechanism in bolstering CRC abundance via the Hedgehog signaling pathway, ultimately driving radioresistance, as confirmed by both bulk RNA-seq and scRNA-seq analyses.

While recent strides in predicting preRT response in CRC show promise, hurdles persist (35, 36). On the molecular front, previous studies have attempted to predict preRT response using gene sets such as transient receptor potential channels (37), DNA Damage Response (38), and fatty acid metabolism (39). However, the limitations of single gene sets underscore the need for more comprehensive approaches. Enter machine learning algorithms, adept at uncovering patterns and correlations within vast datasets (40, 41). In our study, we harnessed machine learning to construct 17 models predicting preRT response across four public cohorts. The top four models, namely glmBoost, random forest, ridge, and LASSO, yielded mean AUC value as high as 0.842. Thus, the fusion of machine learning and transcriptomics holds promise in predicting preRT response more accurately.

EIF5A’s importance in cancer progression has garnered attention, but its full significance remains to be uncovered. The biological role and significance of EIF5A lies in its pivotal regulation of essential cellular processes like protein synthesis (42), tumor proliferation (43), and apoptosis (44), which are crucial for cancer development (45). While its significance has been noted in various cancers, including neuroblastoma (46), lung adenocarcinoma (47), and hepatocellular carcinoma (43), its link to radioresistance remained uncharted territory. In this study, EIF5A was pinpointed as a key player in CRC radioresistance through meticulous analysis of Shapley values and comprehensive expression profiling, which was verified by bulk RNA-seq, scRNA-seq and in-house cohort. Of note, knock down of EIF5A was found to reverse the radioresistance of CRC *in vitro*. Therefore, EIF5A might be a potential target to overcome radioresistance in CRC, but the underlying mechanism is still to be explored.

EIF5A has been implicated in several signaling pathways that are also important in the regulation of CSCs, including pathways of NF-kB, Wnt, and PI3K/AKT (48, 49). It is well known that increased CSCs is an important cause of radioresistance (12, 50). As is expected, the proportion of CSC in the radioresistant cells was significantly higher than that in the radiosensitive cells using scRNA-seq in this study. Further, pseudotime analysis revealed an apparent transformation of epithelial cells into CSCs in the high-EIF5A cells, compared with the low-EIF5A ones. In addition, EIF5A was found to be positively correlated with stemness, and knock down of EIF5A significantly decreased the ability of tumor cells to form sphere *in vitro*. Together, EIF5A might induce the radioresistance via increasing the abundance of CSC within tumors, but it has yet to be known how EIF5A regulated the of epithelial cells into stem cells.

Hedgehog signaling pathway plays a critical role in the regulation of cell growth and differentiation during embryonic development, and it is also implicated in the maintenance of CSCs and therapeutic resistance (51, 52). Hedgehog signaling pathway is often regulated by ligand processing and secretion of dispatched protein and SCUBE2, GPRK2, GRK2, interacted with extracellular matrix of heparan sulfate proteoglycans, and affected by the hypoxia environment (53–55). In this study, hedgehog signaling pathway was upregulated in the radioresistant samples via scRNA-seq, compared with that in the radiosensitive ones. Further, knock down of EIF5A could decreased the expression of SHH, SMO and GLI1, as well as decreased CD44 and CD133, which ultimately resulted in an reversion of radioresistance. Therefore, we concluded that EIF5A could increase the abundance of CSCs via Hedgehog signaling pathway and leading to radioresistance in CRC, but it needs further validation.

TiME is generally categorized into three types: immune-inflamed, immune-rejected, and immune-desert (30). In our research, we observed the lowest distribution of infiltrated immune cells in the radiation-resistant subgroup, conforming to the characteristics of the “immune desert” immunophenotype. To understand the impact of EIF5A on the TiME, we utilized scRNA-seq and bulk RNA-seq analyses, and our findings revealed a notable increase in the infiltration of CD8+ T cells and M1 macrophages in the low-EIF5A subgroup. But on the contrary, the infiltration of M2 macrophages and Tregs was significantly reduced. *In vivo* experiments further demonstrated that EIF5A knockdown led to increased infiltration of CD8+ T cells and M1 macrophages and decreased infiltration of M2 macrophages and Tregs following radiation therapy. This modulation ultimately improved the efficacy of radiotherapy. We propose that EIF5A enhances the positive effects and inhibits the negative effects of radiation on the TiME, making EIF5A a crucial gene for optimizing the radiation-induced TiME. However, these findings deserve further validation.

Whereas, there are several limitations in this study. First, the study’s sample size, particularly in the *in vivo* experiments and the specific patient cohorts, may not fully represent the heterogeneity of colorectal cancer across different populations. Larger and more diverse cohorts are needed to generalize the findings. Second, the use of specific cell lines and animal models may not accurately reflect the complexity and variability of human colorectal cancer and it’s TiME, potentially limiting the applicability of the results to clinical settings. Third, while the study focuses on EIF5A as a key gene associated with radioresistance, this singular focus may overlook the contributions of other genes and pathways that also play significant roles in modulating radioresistance and the TiME. A more comprehensive analysis of multiple genes and pathways might provide a fuller understanding. Last, the study primarily assesses the impact of EIF5A at specific time points, without fully exploring the dynamic changes over time in gene expression and immune cell infiltration in response to radiation and EIF5A modulation. Additionally, while the study suggests that EIF5A influences the TiME and CSC abundance via the Hedgehog signaling pathway, the exact molecular mechanisms and interactions remain to be fully elucidated and require further detailed investigation.

## 5 Conclusions

In conclusion, this study highlights EIF5A as a critical gene influencing radiation sensitivity and the TiME in colorectal cancer. Knockdown of EIF5A significantly enhances radiation sensitivity, reducing tumor volume and increasing apoptosis. Additionally, EIF5A modulation positively impacts TiME by increasing the infiltration of CD8+ T cells and M1 macrophages while reducing M2 macrophages and Tregs. These findings suggest that targeting EIF5A could optimize radiotherapy outcomes and improve immune responses in colorectal cancer patients. Nevertheless, further validation and mechanistic studies are warranted to fully exploit EIF5A’s therapeutic potential in CRC.

## Data Availability

The datasets presented in this study can be found in online repositories. The names of the repository/repositories and accession number(s) can be found in the article/supplementary material.
