# Supplementary material for: Deciphering colorectal cancer radioresistance and immune microrenvironment: unraveling the role of EIF5A through single-cell RNA sequencing and machine learning
Source: Front Immunol. 2024 Sep 3;15:1466226. doi: 10.3389/fimmu.2024.1466226 (PMC11405197; doi:10.3389/fimmu.2024.1466226)
Supplement: Supplementary file 1 [file Table1.docx]

Supplementary Material

# Supplementary tables S1-5:

**Table S1.** Clinical characteristics of the CRC patients receiving preRT in this study.

|  | GSE87211 | GSE35452 | GSE68204 | GSE145037 | GSE150082 |
| --- | --- | --- | --- | --- | --- |
| **No. of patients** | 363 | 46 | 58 | 31 | 39 |
| **Age** |  |  |  |  |  |
| ≤65(%) | 207(57.0%) | NA | 30(50.0%) | 24(77.4%) | 26(66.7%) |
| ＞65(%) | 154(42.4%) | NA | 30(50.0%) | 7(22.6%) | 13(33.3%) |
| unknown | 2(0.6%) | NA | 0(0.0%) | 0(0.0%) | 0(0.0%) |
| **Gender (%)** |  |  |  |  |  |
| Female | 115(31.6%) | NA | 19(32.7%) | 15(48.3%) | 10(25.6%) |
| Male | 248(68.4%) | NA | 39(67.3%) | 16(51.7%) | 29(74.4%) |
| **Stage (%)** |  |  |  |  |  |
| Ⅰ | NA | NA | NA | 0(0.0%) | NA |
| Ⅱ | NA | NA | NA | 1(3.2%) | NA |
| Ⅲ | NA | NA | NA | 16(51.6%) | NA |
| Ⅳ | NA | NA | NA | 14(45.1) | NA |
| **Radiotherapy Response(%)** |  |  |  |  |  |
| Sensitivity | NA | 24(52.1%) | 26(44.8%) | 20(64.5%) | 16(41.1%) |
| Resistance | NA | 22(47.8%) | 32(55.1%) | 11(35.4%) | 23(58.9%) |

*Note:* **CRC:** Colorectal cancer; **preRT:** Preoperative radiotherapy.

**Table S2.** The Primers sequence used in this study.

| **Name** | **Forward-primer** | **Reverse-primer** |
| --- | --- | --- |
| EIF5A | 5'-TTAGATGGGGAGGGAAGAGG-3' | 5'-AATGTGTCGGGGAGAGAGC-3' |
| β-actin | 5'- GAAGAGCTACGAGCTGCCTGA-3' | 5'-CAGACAGCACTGTGTTGGCG-3' |

**Table S3.** List of antibodies western blot.

| **Primary antibodies for western blot** | **Supplier** | **Catalogue number** | **Dilution** |
| --- | --- | --- | --- |
| HRP Anti-beta Actin antibody | abcam | ab49900 | 1:25000 |
| Anti-gamma H2A.X (phospho S139) Rabbit mAb antibody | abcam | ab22551 | 1:5000 |
| Anti-Patched/ PTCH1 Rabbit mAb antibody | abcam | ab53715 | 1:1000 |
| Anti-Gli1antibody | abcam | ab289368 | 1:100 |
| Anti-CD44antibody | abcam | ab157107 | 1:2000 |
| Anti-CD133antibody | abcam | ab68481 | 1:1000 |
| Anti-SHH antibody | Santa cruz | sc-365112 | 1:1000 |
| Anti-SMO/Smoothened antibody | Santa cruz | sc-166685 | 1:1000 |
| Anti-CD4 antibody | Bioss | bs-0766R | 1:200 |
| Anti-CD8 antibody | Bioss | bs-10699R | 1:200 |
| Anti-FOXP3 antibody | Proteintech | 65089-1-Ig | 1:200 |
| Anti-iNOS antibody | Proteintech | 80517-1-RR | 1:200 |
| Anti-ARG-1 antibody | Proteintech | 66129-1-Ig | 1:800 |

**Table S4.** 508 DEGs between radiosensitive- and radioresistant samples.

| **Number** | **Gene** | **logFC** | **AveExpr** | **t** | **P.Value** |
| --- | --- | --- | --- | --- | --- |
| 1 | EIF5A | 0.403020608 | 2.785986943 | 4.006333795 | 9.05528E-05 |
| 2 | NOL3 | 0.278620533 | 4.432403812 | 3.994736972 | 9.47151E-05 |
| 3 | S100A5 | 0.653496587 | 2.744389624 | 3.994687747 | 9.47332E-05 |
| 4 | SYT17 | 0.516067026 | 2.49188423 | 3.876766401 | 0.000148792 |
| 5 | CYP2E1 | 0.399401743 | 1.290708886 | 3.810932318 | 0.00019061 |
| 6 | BEGAIN | 0.460069662 | 1.857968148 | 3.738684013 | 0.000249236 |
| 7 | ELMO3 | 0.436990622 | 4.68496375 | 3.696626874 | 0.00029083 |
| 8 | AP1G2 | 0.279751078 | 3.213158838 | 3.503312492 | 0.0005813 |
| 9 | TYK2 | 0.240946026 | 4.399621387 | 3.480654301 | 0.000629298 |
| 10 | CENPT | 0.247015227 | 3.138667268 | 3.45785946 | 0.000681319 |
| 11 | HSPB3 | -1.058604889 | 1.634274906 | -3.421166045 | 0.000773602 |
| 12 | B3GNT9 | 0.233923663 | 3.385221532 | 3.40239406 | 0.000825219 |
| 13 | ACO2 | -0.252291509 | 5.219944344 | -3.391255445 | 0.000857352 |
| 14 | NOTCH4 | 0.336868667 | 3.516612852 | 3.387941526 | 0.000867135 |
| 15 | CDK10 | 0.232104694 | 3.664671117 | 3.378837626 | 0.000894552 |
| 16 | GIGYF1 | 0.341786261 | 3.390758859 | 3.375496943 | 0.000904814 |
| 17 | RPS6KA4 | 0.234050204 | 3.505044897 | 3.366942923 | 0.000931596 |
| 18 | CEP44 | -0.291061407 | 2.359930133 | -3.365861972 | 0.000935032 |
| 19 | IFITM10 | 0.295186638 | 1.403955414 | 3.356738607 | 0.000964512 |
| 20 | TSLP | 0.400182851 | 1.359968245 | 3.347161223 | 0.000996391 |
| 21 | TMEM63A | 0.246714015 | 4.670161066 | 3.341276078 | 0.001016466 |
| 22 | STK19 | 0.207901921 | 3.660195171 | 3.333247083 | 0.001044462 |
| 23 | DMKN | 0.62869658 | 3.942627294 | 3.316426846 | 0.001105461 |
| 24 | CDK5RAP3 | 0.242734116 | 5.154198076 | 3.30437647 | 0.001151188 |
| 25 | CCDC24 | 0.377234743 | 4.175405788 | 3.277902416 | 0.001257906 |
| 26 | MAGEA11 | -0.812716536 | 1.584874307 | -3.27732147 | 0.001260348 |
| 27 | CXCL11 | -0.918280143 | 4.350947709 | -3.26219203 | 0.001325518 |
| 28 | CREBL2 | -0.242647356 | 3.573870223 | -3.250218525 | 0.001379303 |
| 29 | ATG4C | -0.210816161 | 2.740561846 | -3.248164835 | 0.001388729 |
| 30 | ANKZF1 | 0.47550775 | 3.518223698 | 3.243367959 | 0.001410981 |
| 31 | SIGIRR | 0.202851056 | 5.019571269 | 3.234966076 | 0.001450756 |
| 32 | TMPRSS5 | 0.537157248 | 3.07677803 | 3.205904835 | 0.001596513 |
| 33 | SGSM2 | 0.255159518 | 3.255469248 | 3.202023796 | 0.001616976 |
| 34 | S100P | 0.430958789 | 7.70062535 | 3.179689176 | 0.001739547 |
| 35 | KLC1 | 0.227490424 | 3.983699501 | 3.178923072 | 0.0017439 |
| 36 | XPNPEP3 | -0.239499551 | 2.497776019 | -3.171211307 | 0.001788287 |
| 37 | CXorf58 | 0.390818962 | 1.159624756 | 3.149117128 | 0.001921321 |
| 38 | CRKL | -0.245666035 | 2.845199605 | -3.126752785 | 0.002065267 |
| 39 | HPCAL1 | 0.230628623 | 4.134212842 | 3.119148315 | 0.002116443 |
| 40 | MUC5B | 0.697471377 | 4.095260241 | 3.115974102 | 0.002138149 |
| 41 | PLEKHN1 | 0.407759298 | 2.628713566 | 3.113369903 | 0.002156111 |
| 42 | USP35 | 0.21566034 | 3.354875566 | 3.101273427 | 0.002241387 |
| 43 | IDH3A | -0.234050397 | 4.07524491 | -3.082387365 | 0.002380779 |
| 44 | GRK5 | 0.226285746 | 4.449612394 | 3.077417096 | 0.00241877 |
| 45 | GNA13 | -0.212978079 | 4.225909042 | -3.057812041 | 0.002574147 |
| 46 | MC4R | -0.286212936 | 1.081304567 | -3.034199175 | 0.002773488 |
| 47 | NPHP4 | 0.249792803 | 3.173414326 | 3.028491315 | 0.002823763 |
| 48 | ZNF692 | 0.400173768 | 4.127886731 | 3.02640826 | 0.002842318 |
| 49 | TSTA3 | 0.305226444 | 5.200457866 | 3.01424207 | 0.002952953 |
| 50 | COL27A1 | 0.290901782 | 3.674437935 | 2.984872695 | 0.003236551 |
| 51 | GIPC2 | -0.378889593 | 3.951732707 | -2.981352783 | 0.00327217 |
| 52 | NKAIN4 | 0.299366769 | 0.891012551 | 2.979858407 | 0.003287401 |
| 53 | PYROXD2 | 0.323507227 | 2.145261401 | 2.978743519 | 0.003298806 |
| 54 | OSBPL11 | -0.20911953 | 3.538186079 | -2.978240655 | 0.003303962 |
| 55 | RARG | 0.258230876 | 2.856875351 | 2.975978858 | 0.003327246 |
| 56 | HMGN5 | -0.369620148 | 2.524306283 | -2.961970354 | 0.00347484 |
| 57 | ZFHX3 | 0.26920853 | 3.713075674 | 2.958500739 | 0.003512313 |
| 58 | ZNF513 | 0.208290048 | 4.04129422 | 2.958382179 | 0.0035136 |
| 59 | IL1RAPL2 | 0.307376798 | 1.446360994 | 2.952103086 | 0.003582383 |
| 60 | ALS2CL | 0.250063152 | 3.620032948 | 2.951915967 | 0.003584452 |
| 61 | TCIRG1 | 0.272537455 | 5.063423915 | 2.949726127 | 0.003608742 |
| 62 | SNRNP70 | 0.211235706 | 5.652909208 | 2.949447309 | 0.003611845 |
| 63 | FAM8A1 | -0.256894076 | 3.356952268 | -2.949259906 | 0.003613933 |
| 64 | TIGIT | -0.410806101 | 3.089738312 | -2.947724724 | 0.003631074 |
| 65 | SYT8 | 0.451979321 | 1.543469563 | 2.93140892 | 0.00381791 |
| 66 | ANKRD13D | 0.210893973 | 2.896031831 | 2.930452864 | 0.003829127 |
| 67 | MYO3B | 0.311161275 | 1.24571399 | 2.926198592 | 0.003879406 |
| 68 | AIF1L | 0.279654983 | 2.647276281 | 2.912012312 | 0.004051468 |
| 69 | LIME1 | 0.328658106 | 3.619158835 | 2.911625388 | 0.004056257 |
| 70 | KNCN | -0.272483064 | 1.268927264 | -2.90538928 | 0.004134165 |
| 71 | KIAA0895L | 0.214089472 | 3.012711371 | 2.902007087 | 0.004176989 |
| 72 | GPR65 | -0.373833231 | 2.92649041 | -2.899234089 | 0.004212403 |
| 73 | ZNF232 | 0.243289293 | 3.475047083 | 2.881142769 | 0.004450264 |
| 74 | SLFNL1 | 0.329711663 | 1.494577485 | 2.876806016 | 0.004509077 |
| 75 | C12orf75 | -0.261346399 | 5.012211339 | -2.86801087 | 0.004630539 |
| 76 | CLK2 | 0.268360927 | 3.453208935 | 2.867364424 | 0.004639583 |
| 77 | MC1R | 0.261058796 | 3.272811253 | 2.86514571 | 0.004670748 |
| 78 | SPATA2 | -0.252346173 | 2.701331769 | -2.863232151 | 0.004697779 |
| 79 | CCDC155 | -0.236866195 | 1.098255681 | -2.851424821 | 0.004867757 |
| 80 | ACAP3 | 0.208168457 | 2.572839839 | 2.85099258 | 0.004874085 |
| 81 | USP38 | -0.311646511 | 3.217352368 | -2.846026443 | 0.004947325 |
| 82 | PABPC1L | 0.444866773 | 3.747791557 | 2.837335377 | 0.005077921 |
| 83 | CD2AP | -0.205957868 | 4.597450703 | -2.827140201 | 0.005235118 |
| 84 | NXT2 | -0.253142212 | 3.416505669 | -2.824905851 | 0.005270156 |
| 85 | PAQR6 | 0.308697896 | 2.814832383 | 2.820582295 | 0.005338563 |
| 86 | HSD17B4 | -0.224134319 | 5.06689606 | -2.818044843 | 0.005379086 |
| 87 | VAX2 | 0.411154273 | 1.197576251 | 2.816584153 | 0.00540254 |
| 88 | TPMT | -0.243892134 | 4.77982658 | -2.805431362 | 0.005584711 |
| 89 | CCDC78 | 0.382199548 | 1.923670221 | 2.804978566 | 0.005592224 |
| 90 | SAMHD1 | -0.320095794 | 2.302201153 | -2.804974283 | 0.005592295 |
| 91 | LAMA5 | 0.318731732 | 3.200258723 | 2.804262897 | 0.005604117 |
| 92 | ADCK5 | 0.304480319 | 4.172671628 | 2.800925553 | 0.005659881 |
| 93 | TRIM45 | 0.226000097 | 2.297434271 | 2.795008741 | 0.005759989 |
| 94 | NEUROG3 | 0.41769907 | 4.144315267 | 2.794687769 | 0.005765465 |
| 95 | CHM | -0.223354582 | 3.293774681 | -2.792750783 | 0.005798614 |
| 96 | GPSM1 | 0.24941365 | 3.657448105 | 2.790805313 | 0.005832083 |
| 97 | MAMLD1 | 0.278735994 | 1.969404345 | 2.782260976 | 0.005981163 |
| 98 | USP12 | -0.289042762 | 3.994295671 | -2.775845838 | 0.00609536 |
| 99 | HTR2C | -0.327900399 | 1.10460897 | -2.769785262 | 0.006205061 |
| 100 | CAPN12 | 0.456563549 | 3.978749221 | 2.762253435 | 0.006343889 |
| 101 | FAM20C | 0.24593797 | 4.256273468 | 2.759939402 | 0.006387105 |
| 102 | AQP5 | 0.35315971 | 3.000438131 | 2.754695064 | 0.006486035 |
| 103 | PXMP2 | -0.244070173 | 4.639285694 | -2.749026478 | 0.006594531 |
| 104 | PRKX | -0.277601805 | 3.749108233 | -2.745340133 | 0.006665967 |
| 105 | GLIS1 | 0.243540164 | 2.030670252 | 2.739442323 | 0.006781718 |
| 106 | KCNA10 | -0.309446167 | 1.418961097 | -2.735150953 | 0.006867082 |
| 107 | MXD3 | 0.249105169 | 2.803964791 | 2.725384427 | 0.007065 |
| 108 | NOXA1 | 0.25244081 | 4.50333659 | 2.719606167 | 0.007184514 |
| 109 | MAN2C1 | 0.28944942 | 3.595636513 | 2.719132601 | 0.007194389 |
| 110 | RNF144B | -0.32479843 | 2.864752841 | -2.716450014 | 0.007250563 |
| 111 | KRT2 | 0.272752316 | 1.18069794 | 2.714142273 | 0.007299205 |
| 112 | ING2 | -0.232467229 | 4.05624669 | -2.713525296 | 0.00731226 |
| 113 | KLRD1 | -0.389903046 | 2.144509359 | -2.70590904 | 0.007475159 |
| 114 | TNKS | -0.331360652 | 2.414104567 | -2.700275396 | 0.007597757 |
| 115 | CYP2A7 | -0.289652379 | 1.199335972 | -2.695709812 | 0.007698442 |
| 116 | SNCAIP | 0.441996535 | 2.136789683 | 2.694543482 | 0.007724356 |
| 117 | MEGF6 | 0.395967022 | 2.41066342 | 2.694109251 | 0.007734024 |
| 118 | IFNK | 0.335970262 | 1.462581864 | 2.690220876 | 0.007821086 |
| 119 | ATG16L2 | 0.250903412 | 3.923891149 | 2.68838016 | 0.007862609 |
| 120 | NMU | 0.661244777 | 3.845344102 | 2.685930795 | 0.007918169 |
| 121 | ZNF708 | -0.22661262 | 1.932892831 | -2.683557599 | 0.00797234 |
| 122 | TACO1 | -0.210781279 | 3.99090413 | -2.681526402 | 0.00801897 |
| 123 | PLEC | 0.277907904 | 4.499318695 | 2.681420481 | 0.008021408 |
| 124 | ADARB1 | 0.245864952 | 2.411519315 | 2.681381273 | 0.008022311 |
| 125 | SATB1 | 0.345910229 | 3.489406168 | 2.67859897 | 0.008086608 |
| 126 | SNX25 | -0.234799416 | 3.159272624 | -2.676298049 | 0.008140132 |
| 127 | NPW | 0.528380096 | 2.315378554 | 2.675906737 | 0.008149266 |
| 128 | EVI5 | -0.200810173 | 3.484113876 | -2.671743707 | 0.008247018 |
| 129 | FGF18 | 0.422232805 | 2.540653286 | 2.671727862 | 0.008247392 |
| 130 | TTYH3 | 0.216417992 | 3.47661869 | 2.668170103 | 0.008331772 |
| 131 | AQP3 | 0.498093906 | 3.284989447 | 2.66786765 | 0.008338981 |
| 132 | PNPLA4 | -0.287984973 | 3.862766511 | -2.666558246 | 0.008370256 |
| 133 | TNFRSF9 | -0.416751195 | 2.274944227 | -2.665830064 | 0.008387694 |
| 134 | ZNF114 | 0.377169211 | 0.639320993 | 2.663482368 | 0.00844414 |
| 135 | LTK | 0.44714465 | 3.289923136 | 2.657970493 | 0.008578011 |
| 136 | SCPEP1 | -0.285874251 | 3.419870892 | -2.65227919 | 0.008718245 |
| 137 | ARL5B | -0.231808583 | 3.919372944 | -2.649264923 | 0.008793351 |
| 138 | MYO5A | -0.301221906 | 2.105600777 | -2.648136643 | 0.008821613 |
| 139 | CREB3L4 | 0.250040787 | 4.460422488 | 2.647835888 | 0.008829161 |
| 140 | ANXA1 | 0.430426603 | 4.747024109 | 2.647043759 | 0.008849067 |
| 141 | FSD1 | 0.356158415 | 1.757947477 | 2.644185273 | 0.008921239 |
| 142 | BATF2 | -0.307402336 | 4.998554736 | -2.643772837 | 0.008931696 |
| 143 | ATP13A2 | 0.206453284 | 3.729608474 | 2.638646905 | 0.009062582 |
| 144 | EMID1 | 0.303292737 | 3.126340198 | 2.636946464 | 0.009106382 |
| 145 | RSAD2 | -0.51329075 | 2.973992119 | -2.631701005 | 0.009242696 |
| 146 | UNC93B1 | 0.202569217 | 3.981074826 | 2.630266123 | 0.009280303 |
| 147 | ZNF554 | -0.240181895 | 2.218205867 | -2.629194047 | 0.009308491 |
| 148 | SLC4A11 | 0.421217588 | 2.912327424 | 2.627978402 | 0.009340547 |
| 149 | CELF3 | 0.356847316 | 2.269229787 | 2.625439049 | 0.009407829 |
| 150 | CBX8 | 0.302708945 | 2.936562012 | 2.622847811 | 0.009476935 |
| 151 | CHRNA1 | -0.303978297 | 1.661019124 | -2.621700279 | 0.009507684 |
| 152 | TMEM88 | 0.32198203 | 2.81675847 | 2.619006383 | 0.009580222 |
| 153 | CDNF | -0.343830198 | 0.875927579 | -2.618532254 | 0.00959304 |
| 154 | SHANK3 | 0.405892004 | 2.943283226 | 2.617897037 | 0.009610238 |
| 155 | ZNF335 | 0.203107174 | 3.345817704 | 2.616992187 | 0.009634783 |
| 156 | MST1R | 0.23115573 | 5.071974225 | 2.604576198 | 0.009977321 |
| 157 | PTPN7 | -0.319080426 | 2.534223187 | -2.603867625 | 0.009997195 |
| 158 | STAG3 | 0.258318057 | 3.094994256 | 2.602699775 | 0.010030029 |
| 159 | FSCN2 | 0.345575669 | 2.518906785 | 2.602619315 | 0.010032295 |
| 160 | GTSF1 | -0.439799214 | 1.44512711 | -2.596398523 | 0.010208866 |
| 161 | TFPI | 0.332913935 | 3.388751171 | 2.59343015 | 0.010294099 |
| 162 | MUC5AC | 0.415493527 | 2.579836521 | 2.593279669 | 0.010298437 |
| 163 | KLRC4 | -0.410736008 | 1.712120562 | -2.591214289 | 0.010358141 |
| 164 | BTBD11 | 0.522212137 | 1.374753463 | 2.591093674 | 0.010361637 |
| 165 | HERC5 | -0.426122274 | 1.702419859 | -2.590826603 | 0.010369382 |
| 166 | SLC2A5 | -0.260777861 | 2.660652669 | -2.589548428 | 0.010406522 |
| 167 | PRKAG3 | -0.242601562 | 1.103891058 | -2.587923757 | 0.010453901 |
| 168 | KLHL6 | -0.310133901 | 2.275423266 | -2.585881466 | 0.010513735 |
| 169 | CHKB | 0.212376424 | 4.101484022 | 2.583071036 | 0.010596575 |
| 170 | UMODL1 | -0.378843579 | 1.721367111 | -2.582220369 | 0.010621764 |
| 171 | CYTH3 | -0.266668399 | 1.978604114 | -2.581055237 | 0.010656352 |
| 172 | C4BPB | 0.451937538 | 3.804389037 | 2.579398244 | 0.010705716 |
| 173 | NTRK2 | -0.458977094 | 2.325651364 | -2.578159702 | 0.010742747 |
| 174 | SLC4A3 | 0.315673867 | 1.691158464 | 2.577231415 | 0.010770577 |
| 175 | PES1 | 0.230252601 | 3.707248074 | 2.57404538 | 0.010866588 |
| 176 | TMPRSS3 | 0.468673573 | 2.624982943 | 2.566717563 | 0.011090326 |
| 177 | MALAT1 | 0.291922032 | 5.7035521 | 2.565635448 | 0.011123713 |
| 178 | MATR3 | -0.223728874 | 4.760275126 | -2.559852047 | 0.011303684 |
| 179 | LRRC8E | 0.272083341 | 3.384157031 | 2.556890099 | 0.011396862 |
| 180 | THAP2 | -0.292315322 | 2.133854426 | -2.552489685 | 0.01153656 |
| 181 | EIF1B | -0.204212462 | 4.634653719 | -2.552282154 | 0.011543186 |
| 182 | SLC17A9 | 0.388292134 | 2.952663986 | 2.544486249 | 0.011794576 |
| 183 | MPP2 | 0.237214762 | 1.08463585 | 2.54436844 | 0.011798412 |
| 184 | SMAD9 | 0.407772202 | 2.57949202 | 2.531516949 | 0.012223652 |
| 185 | ERN2 | 0.273440328 | 4.427783608 | 2.522470982 | 0.012531131 |
| 186 | WT1 | -0.523256576 | 1.233176165 | -2.521252623 | 0.012573068 |
| 187 | LRIF1 | -0.267765789 | 2.972304996 | -2.516174515 | 0.012749211 |
| 188 | LAG3 | -0.316124553 | 3.134304119 | -2.510326436 | 0.012954788 |
| 189 | TCF7L1 | 0.301295278 | 1.681061782 | 2.50662784 | 0.013086326 |
| 190 | FOXJ1 | -0.542523122 | 2.589863694 | -2.505655673 | 0.013121097 |
| 191 | TNNI3K | -0.31723561 | 1.396852457 | -2.504223106 | 0.013172486 |
| 192 | CDKN1C | 0.362422118 | 3.508560543 | 2.499808074 | 0.013331992 |
| 193 | ZBBX | 0.353150888 | 1.185561252 | 2.49842994 | 0.013382133 |
| 194 | OSBP2 | 0.301115473 | 2.445898452 | 2.497456737 | 0.013417642 |
| 195 | CORIN | -0.378628597 | 1.29403245 | -2.4971627 | 0.013428387 |
| 196 | LINC00466 | -0.249252086 | 1.040773665 | -2.494958913 | 0.013509164 |
| 197 | FAM47B | -0.275519887 | 0.844487544 | -2.493082764 | 0.013578273 |
| 198 | CDC14A | -0.276497412 | 1.484813783 | -2.491091284 | 0.013651975 |
| 199 | MAP3K6 | 0.213454648 | 3.637917328 | 2.486584741 | 0.013820073 |
| 200 | KCNV1 | -0.552521627 | 1.724245523 | -2.484338078 | 0.013904562 |
| 201 | DYDC2 | 0.5208371 | 1.159088461 | 2.484094866 | 0.013913736 |
| 202 | ADAM15 | 0.224026198 | 4.179576123 | 2.483583701 | 0.013933035 |
| 203 | FOLR1 | 0.499822786 | 2.625532651 | 2.483545728 | 0.013934469 |
| 204 | KBTBD8 | -0.246939842 | 1.97164307 | -2.482483914 | 0.013974637 |
| 205 | IFNG | -0.427553039 | 2.45972953 | -2.481116268 | 0.014026526 |
| 206 | PKN3 | 0.227318988 | 2.302818949 | 2.478229945 | 0.014136597 |
| 207 | GABRB1 | -0.34376606 | 0.996024156 | -2.468609567 | 0.014509044 |
| 208 | BRDT | -0.341196395 | 1.551269745 | -2.468323677 | 0.014520245 |
| 209 | CD96 | -0.371718655 | 2.111710663 | -2.460679521 | 0.014822586 |
| 210 | RORB | 0.226531912 | 0.89979712 | 2.459943963 | 0.014851972 |
| 211 | RPRM | -0.334873 | 0.727056901 | -2.458503764 | 0.014909658 |
| 212 | WNT4 | 0.325171005 | 3.368173599 | 2.456933422 | 0.014972784 |
| 213 | EGF | -0.556318914 | 1.315118877 | -2.45394253 | 0.015093672 |
| 214 | SLC2A9 | 0.244465486 | 2.845197675 | 2.453478783 | 0.015112493 |
| 215 | PTPRU | 0.316980233 | 1.480490452 | 2.449231868 | 0.015285828 |
| 216 | PDE11A | -0.261019119 | 1.403131213 | -2.448046633 | 0.015334516 |
| 217 | STARD9 | 0.216091879 | 2.107954301 | 2.447938443 | 0.015338968 |
| 218 | BEST4 | -0.43518462 | 4.319238756 | -2.445151415 | 0.015454029 |
| 219 | ARHGEF19 | 0.254612866 | 3.925541464 | 2.443359914 | 0.015528395 |
| 220 | WDR90 | 0.202724281 | 4.419521519 | 2.441376736 | 0.015611087 |
| 221 | SHOX2 | 0.247864596 | 1.436831205 | 2.437866387 | 0.015758415 |
| 222 | C10orf91 | 0.284914268 | 2.157853021 | 2.436114617 | 0.015832397 |
| 223 | PAX4 | 0.270226093 | 2.98572309 | 2.436023735 | 0.015836243 |
| 224 | SLC6A17 | 0.272487737 | 2.257222766 | 2.435422369 | 0.015861717 |
| 225 | ECEL1 | 0.278577069 | 1.121295829 | 2.435183036 | 0.015871865 |
| 226 | KIF24 | 0.27839011 | 2.603189254 | 2.435005142 | 0.015879412 |
| 227 | SYT2 | 0.257651061 | 2.25099459 | 2.429118745 | 0.01613093 |
| 228 | KBTBD11 | -0.267286269 | 5.068302736 | -2.423710007 | 0.016365146 |
| 229 | ZNF154 | 0.299057445 | 1.239821351 | 2.420975229 | 0.016484713 |
| 230 | PRSS22 | 0.332464116 | 4.646514099 | 2.420889207 | 0.016488487 |
| 231 | APOL6 | -0.221102835 | 3.635495382 | -2.420507327 | 0.016505248 |
| 232 | FAM200A | -0.234737479 | 2.780521236 | -2.41884072 | 0.016578573 |
| 233 | SLC22A16 | -0.334782618 | 0.78223359 | -2.417085541 | 0.016656107 |
| 234 | DPYS | -0.262388758 | 1.286720859 | -2.409734311 | 0.016984344 |
| 235 | CMPK1 | -0.202013705 | 5.243653466 | -2.409653706 | 0.016987974 |
| 236 | SLPI | 0.384046861 | 6.00354946 | 2.398981693 | 0.017474764 |
| 237 | TDRD9 | 0.380548672 | 0.895240865 | 2.397606546 | 0.017538378 |
| 238 | FANCA | 0.209157089 | 2.969081719 | 2.397433953 | 0.017546377 |
| 239 | CTTNBP2 | -0.400756334 | 3.70171003 | -2.396227873 | 0.017602361 |
| 240 | GPC2 | 0.25027454 | 2.555661807 | 2.394282601 | 0.01769299 |
| 241 | BRD4 | 0.210492624 | 2.868109067 | 2.391982361 | 0.017800688 |
| 242 | LPCAT1 | 0.255567489 | 2.950761122 | 2.39147211 | 0.017824657 |
| 243 | NDST3 | 0.296840253 | 0.382517777 | 2.389360456 | 0.017924153 |
| 244 | PACSIN2 | -0.211329494 | 3.78353875 | -2.384509518 | 0.018154579 |
| 245 | CD55 | 0.342403911 | 4.759978425 | 2.382208464 | 0.018264794 |
| 246 | LRRC56 | 0.231950681 | 3.579075128 | 2.381370386 | 0.018305082 |
| 247 | TMEM198 | 0.29781614 | 2.79461912 | 2.380383156 | 0.018352641 |
| 248 | ARHGAP32 | 0.214863291 | 4.433592078 | 2.380002398 | 0.018371013 |
| 249 | TEF | -0.313529654 | 3.067861145 | -2.376722623 | 0.018529939 |
| 250 | CXCL13 | -0.639644342 | 3.446550242 | -2.37236934 | 0.018742755 |
| 251 | LINC00189 | -0.311722087 | 0.575662237 | -2.368824716 | 0.018917629 |
| 252 | PTGR2 | -0.259541686 | 2.601166231 | -2.367946758 | 0.018961165 |
| 253 | GNG2 | -0.298243903 | 2.140106921 | -2.362146781 | 0.019250997 |
| 254 | LCA5L | -0.265059509 | 1.202392417 | -2.360821311 | 0.019317778 |
| 255 | IL17RC | 0.324093804 | 3.513355893 | 2.359297176 | 0.01939482 |
| 256 | CGREF1 | 0.388187114 | 4.370178266 | 2.357412593 | 0.019490457 |
| 257 | FLT3 | -0.301471132 | 1.569321914 | -2.353235329 | 0.019703923 |
| 258 | ABLIM3 | 0.221027217 | 2.09763608 | 2.351331479 | 0.019801894 |
| 259 | HSF4 | 0.270822926 | 2.695171186 | 2.350245326 | 0.019857979 |
| 260 | RNF207 | 0.213000196 | 2.716245234 | 2.346084914 | 0.020074104 |
| 261 | KIFC2 | 0.298688778 | 3.475531581 | 2.344635197 | 0.020149898 |
| 262 | CCDC168 | -0.260447759 | 1.451642041 | -2.34448387 | 0.020157825 |
| 263 | MAP3K12 | 0.255156731 | 1.425642577 | 2.343298871 | 0.020219988 |
| 264 | RGL3 | 0.412153434 | 2.59451586 | 2.342474512 | 0.020263331 |
| 265 | AGR2 | 0.35369812 | 7.62915603 | 2.341947496 | 0.020291084 |
| 266 | SLC6A4 | -0.427626166 | 2.071291625 | -2.341628749 | 0.020307886 |
| 267 | MSLN | 0.616798375 | 2.815404076 | 2.338213776 | 0.020488662 |
| 268 | CCR6 | -0.229497135 | 2.724271225 | -2.338070869 | 0.020496257 |
| 269 | ERI1 | -0.225190219 | 3.143322961 | -2.337284663 | 0.02053809 |
| 270 | SNX7 | -0.216164456 | 5.3955421 | -2.334937524 | 0.020663423 |
| 271 | HOXB3 | 0.340552866 | 4.065369893 | 2.3320576 | 0.020818124 |
| 272 | KIF7 | 0.265071506 | 1.512079199 | 2.330468538 | 0.020903919 |
| 273 | TMEM200A | -0.412264186 | 4.210725106 | -2.329340018 | 0.020965038 |
| 274 | SCOC | -0.201819788 | 3.652468798 | -2.328413196 | 0.02101535 |
| 275 | IL22 | -0.316479969 | 1.347577707 | -2.327143253 | 0.021084461 |
| 276 | STK36 | 0.224009491 | 2.903002421 | 2.326508563 | 0.021119076 |
| 277 | SRD5A2 | 0.217384372 | 1.265616835 | 2.325359108 | 0.021181892 |
| 278 | MDK | 0.326256064 | 4.768354665 | 2.324994329 | 0.021201861 |
| 279 | FBXL22 | -0.304034228 | 1.448730319 | -2.3236531 | 0.021275426 |
| 280 | PIF1 | 0.212524972 | 3.20526303 | 2.320352472 | 0.021457417 |
| 281 | CLIC3 | 0.391181679 | 3.13739817 | 2.31881043 | 0.02154291 |
| 282 | NEURL3 | -0.357059336 | 3.325431804 | -2.317806632 | 0.021598723 |
| 283 | MCPH1 | -0.231786346 | 2.300192965 | -2.317390431 | 0.021621902 |
| 284 | WDR7 | -0.256720911 | 3.382881946 | -2.315620964 | 0.021720689 |
| 285 | CPEB3 | -0.246679324 | 2.726376996 | -2.311118389 | 0.02197385 |
| 286 | WDR78 | -0.329742385 | 2.443351075 | -2.307312081 | 0.022189879 |
| 287 | COL7A1 | 0.370884352 | 3.574509189 | 2.305406545 | 0.022298727 |
| 288 | LALBA | -0.315439539 | 1.500008879 | -2.30394235 | 0.022382682 |
| 289 | ETNK2 | 0.259627234 | 1.137612157 | 2.303745133 | 0.022394011 |
| 290 | SLC27A3 | 0.200115246 | 3.629818706 | 2.299470971 | 0.022640783 |
| 291 | MTA2 | 0.276206048 | 3.633059144 | 2.29706832 | 0.022780546 |
| 292 | ODF4 | -0.28535088 | 1.770306846 | -2.295909154 | 0.022848244 |
| 293 | GLRA3 | 0.213078937 | 0.901704158 | 2.295762968 | 0.022856795 |
| 294 | COLQ | 0.297632328 | 1.242609884 | 2.29503197 | 0.022899592 |
| 295 | PRDM9 | -0.214322151 | 0.495146772 | -2.293505653 | 0.022989178 |
| 296 | SLC12A8 | 0.266250901 | 2.816257518 | 2.292843444 | 0.023028141 |
| 297 | SOCS2 | -0.255019642 | 3.465766123 | -2.28955626 | 0.023222412 |
| 298 | CXCL10 | -0.540921637 | 4.226786199 | -2.287412761 | 0.023349862 |
| 299 | CCDC136 | 0.244936323 | 1.330358373 | 2.284914417 | 0.023499185 |
| 300 | CLCF1 | 0.228075855 | 2.574252222 | 2.283844379 | 0.023563394 |
| 301 | RBPMS | 0.32687613 | 3.131598063 | 2.283058423 | 0.023610655 |
| 302 | CTNNBIP1 | 0.22775491 | 4.735006426 | 2.27930649 | 0.023837408 |
| 303 | FSIP1 | -0.555075183 | 2.190308218 | -2.277341044 | 0.023956951 |
| 304 | KLHL17 | 0.276834518 | 2.086830602 | 2.275121292 | 0.024092592 |
| 305 | RUNDC3B | -0.395931462 | 2.90596194 | -2.273721249 | 0.024178488 |
| 306 | ME3 | 0.220332806 | 4.618135588 | 2.273109538 | 0.024216102 |
| 307 | IFIH1 | -0.227237249 | 4.360083457 | -2.271532134 | 0.024313333 |
| 308 | IRX3 | 0.458531253 | 2.252413734 | 2.271319891 | 0.024326441 |
| 309 | CPNE7 | 0.300320508 | 3.4304735 | 2.270073708 | 0.024403533 |
| 310 | SLC31A2 | -0.222501808 | 4.004369475 | -2.269522129 | 0.024437723 |
| 311 | TGFBR3 | 0.336535609 | 1.929838591 | 2.265501913 | 0.024688186 |
| 312 | DUS3L | 0.201054827 | 3.744269335 | 2.263592072 | 0.024807954 |
| 313 | APOA5 | 0.247175366 | 0.777798838 | 2.262341638 | 0.024886645 |
| 314 | SLC22A4 | -0.335986009 | 2.239677846 | -2.258999689 | 0.025098027 |
| 315 | CAMKK1 | 0.234142232 | 2.979734469 | 2.258171029 | 0.025150683 |
| 316 | GABRR2 | -0.213799004 | 1.112113627 | -2.256715587 | 0.025243399 |
| 317 | CD1D | 0.255651631 | 2.114881251 | 2.255028878 | 0.025351222 |
| 318 | HOXD1 | -0.438510021 | 2.091263619 | -2.249173923 | 0.025728623 |
| 319 | TST | -0.208997712 | 6.968017045 | -2.248898417 | 0.025746502 |
| 320 | IL2RB | -0.265798635 | 3.668729595 | -2.245625292 | 0.025959739 |
| 321 | GTF3C1 | 0.285817454 | 2.835592855 | 2.244741269 | 0.026017595 |
| 322 | TTYH1 | 0.224471671 | 2.172091097 | 2.244042544 | 0.026063404 |
| 323 | EIF2AK3 | -0.209691972 | 3.477257543 | -2.24253973 | 0.026162166 |
| 324 | TSNAXIP1 | 0.284152194 | 1.338826508 | 2.241223041 | 0.026248965 |
| 325 | SERPINB5 | 0.525108167 | 3.916045314 | 2.239812834 | 0.026342206 |
| 326 | LILRB5 | -0.330270763 | 3.413998465 | -2.239258852 | 0.026378914 |
| 327 | LRP5 | 0.205053351 | 3.49852987 | 2.238127114 | 0.026454043 |
| 328 | MLPH | 0.383701536 | 4.642505312 | 2.23167553 | 0.026885896 |
| 329 | TCL1A | -0.517627752 | 3.025769183 | -2.230086252 | 0.026993216 |
| 330 | B3GNT2 | -0.205829571 | 3.583163418 | -2.228916232 | 0.027072462 |
| 331 | DNAH1 | 0.258125397 | 2.249787033 | 2.227264458 | 0.027184683 |
| 332 | SP5 | 0.427601665 | 3.837594263 | 2.225834484 | 0.027282162 |
| 333 | ZIC4 | -0.306659607 | 1.266187435 | -2.22567996 | 0.027292714 |
| 334 | OPCML | 0.241427297 | 1.328501802 | 2.224066821 | 0.027403081 |
| 335 | NAT1 | -0.217080074 | 4.649281157 | -2.223992948 | 0.027408144 |
| 336 | CX3CL1 | 0.274779569 | 3.446418999 | 2.22390621 | 0.02741409 |
| 337 | KLHDC7B | -0.225383578 | 2.151295034 | -2.223091519 | 0.027469996 |
| 338 | PTGR1 | -0.288007742 | 4.172593641 | -2.222816611 | 0.027488884 |
| 339 | LMCD1 | 0.239244392 | 4.44381224 | 2.220881461 | 0.027622156 |
| 340 | ADAMTSL2 | 0.28234302 | 2.821255567 | 2.217037827 | 0.02788853 |
| 341 | TRAT1 | -0.37346345 | 1.450358048 | -2.215788297 | 0.027975606 |
| 342 | HDAC10 | 0.233146549 | 5.088651941 | 2.215534267 | 0.027993337 |
| 343 | FUT2 | 0.203747235 | 5.132950589 | 2.212814749 | 0.028183774 |
| 344 | EPM2AIP1 | 0.21811164 | 3.292569485 | 2.210451271 | 0.028350192 |
| 345 | SLAMF6 | -0.251447887 | 1.437855856 | -2.20939794 | 0.028424634 |
| 346 | RBP2 | -0.620298644 | 4.294142053 | -2.208750174 | 0.028470498 |
| 347 | CTSW | -0.290090327 | 2.062150151 | -2.204460961 | 0.028775812 |
| 348 | TRAF5 | 0.257539517 | 5.143103124 | 2.204378943 | 0.028781678 |
| 349 | LCAT | 0.285238813 | 2.036059239 | 2.204039023 | 0.028806 |
| 350 | EDN2 | -0.456791525 | 2.818990044 | -2.203020565 | 0.028878979 |
| 351 | PCP4 | 0.707765858 | 2.829296802 | 2.20129289 | 0.029003144 |
| 352 | OR5L2 | -0.329446813 | 2.730811779 | -2.201117908 | 0.029015745 |
| 353 | CHUK | -0.205083453 | 3.549480303 | -2.200658655 | 0.029048841 |
| 354 | FAM81A | -0.293726058 | 2.811038315 | -2.198222378 | 0.029224961 |
| 355 | BST1 | -0.353986658 | 2.77786974 | -2.197115527 | 0.029305281 |
| 356 | FAM155B | 0.417916204 | 2.122471032 | 2.196559462 | 0.029345705 |
| 357 | SERPINF2 | 0.2701343 | 1.581827262 | 2.196221366 | 0.029370307 |
| 358 | TNFAIP8L3 | -0.283879107 | 1.945429091 | -2.193499402 | 0.029569027 |
| 359 | GALNT9 | 0.26225496 | 1.494398516 | 2.193136116 | 0.029595637 |
| 360 | BEND7 | -0.218953745 | 3.786385517 | -2.191501424 | 0.029715632 |
| 361 | NGF | 0.233673436 | 1.492773805 | 2.190383695 | 0.029797922 |
| 362 | LINC00477 | -0.214717151 | 1.030699612 | -2.188226087 | 0.02995733 |
| 363 | LPAR2 | 0.234069523 | 3.573295002 | 2.187773443 | 0.029990866 |
| 364 | CD1A | 0.236043961 | 1.628691409 | 2.187264396 | 0.03002862 |
| 365 | KRT16 | 0.470890247 | 1.381482949 | 2.186422214 | 0.030091171 |
| 366 | GLRA2 | -0.466237337 | 2.09769219 | -2.183706885 | 0.030293614 |
| 367 | TPM2 | 0.21050129 | 4.317592871 | 2.182985842 | 0.03034757 |
| 368 | LONRF1 | -0.20844097 | 2.904410799 | -2.179474104 | 0.030611547 |
| 369 | REEP6 | 0.285576903 | 4.164363864 | 2.178607288 | 0.03067701 |
| 370 | DTWD2 | -0.209220545 | 2.124387223 | -2.177293415 | 0.030776467 |
| 371 | SRD5A3 | 0.310291909 | 4.904696533 | 2.177280144 | 0.030777473 |
| 372 | IL1A | -0.403887491 | 2.115114342 | -2.177073588 | 0.030793135 |
| 373 | B3GAT2 | -0.237109579 | 2.370366246 | -2.176680332 | 0.030822972 |
| 374 | SLC50A1 | 0.247200615 | 3.836899453 | 2.174775053 | 0.030967882 |
| 375 | FCRL3 | -0.251611421 | 1.738794436 | -2.171101321 | 0.031248962 |
| 376 | KLHL23 | 0.201630298 | 3.613055169 | 2.170437918 | 0.031299954 |
| 377 | DSC1 | 0.272996184 | 1.052375656 | 2.166636069 | 0.031593571 |
| 378 | PHOX2B | -0.285468497 | 1.558668032 | -2.164654209 | 0.031747573 |
| 379 | SERPINA5 | 0.254832547 | 0.938792182 | 2.163584666 | 0.031830952 |
| 380 | SPTSSB | 0.386658263 | 0.589441257 | 2.159763653 | 0.032130377 |
| 381 | GNGT2 | -0.250254712 | 2.217814141 | -2.159516022 | 0.032149866 |
| 382 | WDR27 | 0.235686516 | 2.022065604 | 2.15478204 | 0.032524404 |
| 383 | CALHM3 | 0.340429769 | 0.892345629 | 2.151136923 | 0.032815356 |
| 384 | H1FOO | -0.241835069 | 1.533333411 | -2.149498742 | 0.032946846 |
| 385 | OR2L13 | 0.246509469 | 1.164121344 | 2.149313328 | 0.032961757 |
| 386 | NUP188 | 0.210380466 | 2.982337764 | 2.147546189 | 0.033104163 |
| 387 | CACNA1H | 0.222684559 | 2.692197625 | 2.146777448 | 0.033166278 |
| 388 | ENKUR | -0.374131814 | 1.793960422 | -2.146108133 | 0.033220441 |
| 389 | CD300LF | -0.237577038 | 2.084155956 | -2.142667785 | 0.033500051 |
| 390 | CNBD1 | 0.225652237 | 0.970591431 | 2.142051466 | 0.033550355 |
| 391 | BAG4 | -0.216456323 | 2.71406721 | -2.141880538 | 0.033564318 |
| 392 | CACNA1I | 0.244509589 | 4.79081256 | 2.141610348 | 0.0335864 |
| 393 | CD101 | -0.233758268 | 1.322895793 | -2.138416442 | 0.033848377 |
| 394 | ADAMTS16 | -0.238007113 | 1.054170081 | -2.137440586 | 0.033928771 |
| 395 | KRI1 | 0.228793897 | 4.545531522 | 2.136946189 | 0.033969563 |
| 396 | MILR1 | -0.274594654 | 1.524131043 | -2.136412718 | 0.034013628 |
| 397 | LRRC43 | -0.222646088 | 1.179892698 | -2.134311039 | 0.034187704 |
| 398 | HIST3H2A | 0.236207828 | 4.289305408 | 2.130274187 | 0.034524221 |
| 399 | CKMT2 | -0.87771234 | 3.692014978 | -2.130083696 | 0.03454017 |
| 400 | DAPK1 | 0.294149021 | 1.948988418 | 2.127439587 | 0.034762218 |
| 401 | GSDMC | 0.205799689 | 1.588281133 | 2.127010968 | 0.034798328 |
| 402 | DRD3 | -0.219517346 | 1.841702039 | -2.125797798 | 0.03490071 |
| 403 | FNDC9 | -0.261380204 | 1.410936923 | -2.123592456 | 0.035087488 |
| 404 | MYBPC3 | 0.300281095 | 2.799822824 | 2.119939736 | 0.035398743 |
| 405 | AFM | -0.377620544 | 0.950519517 | -2.117167621 | 0.035636543 |
| 406 | RAC3 | 0.253924319 | 2.605897103 | 2.116722834 | 0.035674825 |
| 407 | EOMES | -0.280739639 | 1.818692038 | -2.113081551 | 0.035989562 |
| 408 | P2RY1 | -0.372998923 | 2.768166286 | -2.112985481 | 0.035997898 |
| 409 | SRCIN1 | 0.245658725 | 2.271972546 | 2.108631956 | 0.036377405 |
| 410 | HLF | -0.28979352 | 1.117851287 | -2.10817088 | 0.036417798 |
| 411 | CATSPERB | 0.346562877 | 3.472140663 | 2.10600228 | 0.036608301 |
| 412 | DEPDC1B | -0.277441301 | 3.084300397 | -2.105889698 | 0.036618215 |
| 413 | SLC6A5 | -0.224461385 | 2.039049629 | -2.10422792 | 0.036764808 |
| 414 | CLEC1B | -0.255427869 | 1.360858367 | -2.104085096 | 0.036777431 |
| 415 | ZNF429 | -0.253421346 | 3.671679238 | -2.103816196 | 0.036801206 |
| 416 | FAM83E | 0.294044787 | 4.077176495 | 2.103452754 | 0.036833361 |
| 417 | PCF11 | 0.207309584 | 3.503183819 | 2.103074024 | 0.036866895 |
| 418 | ZNF408 | 0.248604268 | 2.422423466 | 2.102822825 | 0.036889151 |
| 419 | ADAD2 | 0.255903711 | 2.024234135 | 2.102633748 | 0.036905911 |
| 420 | FSD1L | -0.270272004 | 2.140477159 | -2.099596358 | 0.037176044 |
| 421 | CCDC54 | -0.213549878 | 1.490823698 | -2.09613803 | 0.037485678 |
| 422 | SAMD11 | 0.202683381 | 2.93761922 | 2.095831971 | 0.037513186 |
| 423 | NOX5 | -0.247300071 | 0.627013778 | -2.095733895 | 0.037522005 |
| 424 | MCOLN2 | -0.380444498 | 3.548642846 | -2.094603833 | 0.037623746 |
| 425 | CMA1 | -0.234317278 | 1.095454968 | -2.094259541 | 0.03765479 |
| 426 | CRTC1 | 0.208028526 | 5.734215377 | 2.087447482 | 0.038273552 |
| 427 | KCNH8 | 0.449862768 | 3.863973427 | 2.086082989 | 0.038398536 |
| 428 | KISS1R | 0.345987363 | 3.414554493 | 2.085267333 | 0.038473415 |
| 429 | GNLY | -0.392029643 | 3.3526277 | -2.084560902 | 0.038538368 |
| 430 | MX1 | -0.378394055 | 4.811064114 | -2.083622668 | 0.038624779 |
| 431 | CCDC140 | 0.207704863 | 0.793681324 | 2.083292086 | 0.038655265 |
| 432 | MBOAT2 | 0.293101916 | 4.26557035 | 2.082955062 | 0.038686367 |
| 433 | GADD45GIP1 | 0.257725503 | 5.345948137 | 2.082331749 | 0.038743944 |
| 434 | MUC1 | 0.343631234 | 5.379164359 | 2.080822525 | 0.03888366 |
| 435 | RBP4 | 0.446872513 | 3.645645988 | 2.078021549 | 0.039144104 |
| 436 | MDFI | 0.239184922 | 3.152391043 | 2.077159504 | 0.03922456 |
| 437 | KCNG4 | -0.284837659 | 1.733518291 | -2.076312804 | 0.039303721 |
| 438 | CCL5 | -0.297387953 | 3.894933265 | -2.075581731 | 0.039372183 |
| 439 | BCL2A1 | -0.281829377 | 3.81844846 | -2.07382228 | 0.039537364 |
| 440 | TCN1 | 0.764706588 | 4.305565792 | 2.071500317 | 0.039756264 |
| 441 | MUC16 | 0.201024812 | 0.884932033 | 2.069329287 | 0.039961871 |
| 442 | SYTL1 | 0.241365673 | 3.556381008 | 2.067900634 | 0.040097668 |
| 443 | REG1A | 0.916274745 | 6.782602439 | 2.064264519 | 0.040445071 |
| 444 | AMT | 0.276491789 | 3.627394689 | 2.063986296 | 0.040471759 |
| 445 | DNAH2 | 0.28089657 | 2.797787217 | 2.062573075 | 0.04060755 |
| 446 | TMEM106A | 0.211316004 | 1.719037829 | 2.061320879 | 0.040728195 |
| 447 | SLC6A19 | -0.468766095 | 3.411659031 | -2.058304074 | 0.041020112 |
| 448 | PIWIL1 | 0.373862038 | 1.657836772 | 2.054160229 | 0.041423999 |
| 449 | SLFN11 | -0.264935209 | 3.126193614 | -2.053464692 | 0.041492122 |
| 450 | CD79B | -0.329844487 | 2.127504932 | -2.052910666 | 0.041546454 |
| 451 | DSCR4 | -0.20172332 | 1.555427841 | -2.050358959 | 0.041797478 |
| 452 | ISM1 | -0.360926176 | 1.575238848 | -2.050017866 | 0.04183113 |
| 453 | CAMK2N2 | 0.240566096 | 3.122750567 | 2.049550812 | 0.041877248 |
| 454 | CHRND | 0.252030052 | 1.686864546 | 2.048709231 | 0.041960457 |
| 455 | ZNF594 | 0.217922725 | 2.194449174 | 2.047508418 | 0.042079429 |
| 456 | GPRIN2 | 0.225642188 | 4.885459237 | 2.045103225 | 0.042318591 |
| 457 | SGTB | -0.202613009 | 1.524320902 | -2.04468737 | 0.042360059 |
| 458 | SERPINB7 | 0.504537414 | 1.714311358 | 2.043395817 | 0.042489071 |
| 459 | CD300C | -0.279244794 | 1.729105905 | -2.04269868 | 0.042558847 |
| 460 | CR2 | -0.373102871 | 3.112126691 | -2.039412878 | 0.042889036 |
| 461 | ADORA3 | -0.256058846 | 2.294533598 | -2.036783207 | 0.043154861 |
| 462 | PPP1R14D | -0.264104971 | 5.220630976 | -2.036373662 | 0.043196387 |
| 463 | FABP1 | -0.538242258 | 8.020964687 | -2.034056196 | 0.043432008 |
| 464 | IDI2 | -0.210845735 | 1.593200321 | -2.033880306 | 0.043449936 |
| 465 | CCND2 | -0.293542188 | 4.497059298 | -2.03217713 | 0.04362386 |
| 466 | RADIL | -0.25187895 | 1.174564609 | -2.026799144 | 0.044176942 |
| 467 | ITGAM | -0.243411047 | 2.361455326 | -2.026398199 | 0.044218414 |
| 468 | GPC4 | -0.238545204 | 4.447828611 | -2.025975157 | 0.044262208 |
| 469 | PRRX1 | -0.353422562 | 1.771918031 | -2.025926086 | 0.04426729 |
| 470 | KIF12 | 0.323654495 | 3.436827205 | 2.02467034 | 0.044397516 |
| 471 | CAPN8 | 0.245305738 | 4.877233626 | 2.023536824 | 0.044515345 |
| 472 | KDM6B | 0.225636012 | 1.744696816 | 2.021591515 | 0.044718182 |
| 473 | HELZ | -0.207865524 | 3.183506205 | -2.02123616 | 0.044755319 |
| 474 | CEP19 | -0.220769108 | 1.83645735 | -2.019153653 | 0.044973486 |
| 475 | VWCE | 0.286988961 | 2.56206951 | 2.015721613 | 0.045335005 |
| 476 | OVGP1 | 0.258508296 | 3.321230794 | 2.013216548 | 0.045600436 |
| 477 | CFTR | -0.288905657 | 6.780628847 | -2.010990541 | 0.045837407 |
| 478 | SCNN1D | 0.216681418 | 2.654068402 | 2.010764161 | 0.045861564 |
| 479 | SYCE1L | 0.281202771 | 4.794894523 | 2.008493791 | 0.046104443 |
| 480 | YPEL3 | 0.232231394 | 3.1400508 | 2.007612845 | 0.046198978 |
| 481 | ZNF319 | -0.200107169 | 2.515749521 | -2.006888491 | 0.046276832 |
| 482 | CASQ1 | 0.250292727 | 1.289045246 | 2.006859784 | 0.04627992 |
| 483 | ANKRD36B | 0.211221643 | 3.600900614 | 2.004427249 | 0.046542202 |
| 484 | MANEA | -0.210828317 | 2.853572326 | -2.004340977 | 0.046551527 |
| 485 | SLC16A4 | 0.331260325 | 2.43252686 | 2.004164475 | 0.04657061 |
| 486 | MYB | -0.280432389 | 5.457735633 | -2.001336764 | 0.046877244 |
| 487 | ZFPM1 | 0.234227529 | 5.106812827 | 1.99858884 | 0.047176866 |
| 488 | LEMD1 | 0.602320361 | 2.84193886 | 1.998050309 | 0.047235775 |
| 489 | SSX2IP | -0.22803048 | 2.684868255 | -1.99793653 | 0.047248229 |
| 490 | CNKSR1 | 0.213090442 | 2.821520739 | 1.996491978 | 0.04740659 |
| 491 | THRSP | 0.227206112 | 1.373481644 | 1.995229174 | 0.047545395 |
| 492 | COL16A1 | 0.230346663 | 5.106666264 | 1.994689357 | 0.047604836 |
| 493 | PARVB | -0.289091392 | 2.506553462 | -1.993876834 | 0.047694423 |
| 494 | CCDC60 | 0.291762234 | 1.85205766 | 1.993470474 | 0.047739282 |
| 495 | ZNF480 | -0.252611112 | 3.905645392 | -1.991811238 | 0.047922817 |
| 496 | TUBG2 | 0.211045991 | 3.295815485 | 1.991185393 | 0.0479922 |
| 497 | ITGA4 | -0.252025588 | 2.042355705 | -1.989505848 | 0.048178819 |
| 498 | SHF | 0.322364664 | 3.028163217 | 1.98743281 | 0.048410008 |
| 499 | MAGEB18 | 0.30207941 | 1.099496923 | 1.986083109 | 0.048561034 |
| 500 | NETO2 | -0.380292436 | 3.126544619 | -1.984378105 | 0.048752386 |
| 501 | HS3ST3B1 | -0.23588084 | 1.894228954 | -1.98307875 | 0.048898641 |
| 502 | HOXB4 | 0.230242644 | 2.611042471 | 1.982626716 | 0.048949609 |
| 503 | HOMER1 | -0.252570809 | 3.453922477 | -1.977425317 | 0.04953932 |
| 504 | PADI1 | 0.272695711 | 2.127944145 | 1.975988173 | 0.049703312 |
| 505 | GCNT4 | -0.210987968 | 1.402934274 | -1.975812239 | 0.04972342 |
| 506 | LRRIQ3 | -0.27008429 | 2.020124576 | -1.975372588 | 0.049773697 |
| 507 | LCE2B | 0.238334285 | 1.596805781 | 1.974673753 | 0.049853703 |
| 508 | BTLA | -0.335750821 | 1.992000062 | -1.974167872 | 0.049911686 |

**Table S5.** 1327 genes were associated with DFS based on univariate Cox regression analysis.

| **Number** | **Gene** | **HR** | **HR.95L** | **HR.95H** | **pvalue** |
| --- | --- | --- | --- | --- | --- |
| 1 | A1BG-AS1 | 1.909233226 | 1.086018278 | 3.356455032 | 0.024663669 |
| 2 | A2M | 2.124474292 | 1.143598541 | 3.946656852 | 0.017098315 |
| 3 | AASDHPPT | 0.316296026 | 0.137255026 | 0.728885338 | 0.006884013 |
| 4 | ABCA10 | 0.557007821 | 0.339483465 | 0.913911119 | 0.020542051 |
| 5 | ABCB10 | 0.269690085 | 0.109336011 | 0.665222201 | 0.004442755 |
| 6 | ABCC8 | 2.003837963 | 1.209283039 | 3.320452244 | 0.006987698 |
| 7 | ABCE1 | 0.371356887 | 0.160176856 | 0.86096045 | 0.020948821 |
| 8 | ABHD13 | 0.509746907 | 0.301785508 | 0.861015197 | 0.011752955 |
| 9 | ABLIM2 | 0.640133888 | 0.416949514 | 0.9827842 | 0.041414546 |
| 10 | ABTB2 | 2.208594885 | 1.152122236 | 4.233831458 | 0.017011504 |
| 11 | ACBD3 | 0.398531062 | 0.189526432 | 0.83802035 | 0.015268172 |
| 12 | ACBD6 | 0.34998639 | 0.137086068 | 0.893529701 | 0.028136935 |
| 13 | ACIN1 | 2.47878495 | 1.142941367 | 5.375931788 | 0.021549585 |
| 14 | ACN9 | 0.492130588 | 0.292637719 | 0.82761893 | 0.007509538 |
| 15 | ACP1 | 0.190792293 | 0.048561411 | 0.749601352 | 0.017654454 |
| 16 | ACSS2 | 2.068757886 | 1.047006097 | 4.087616304 | 0.036423788 |
| 17 | ACTBL2 | 3.115891014 | 1.331059746 | 7.294020302 | 0.008819852 |
| 18 | ACTN3 | 2.425205565 | 1.035792329 | 5.678379598 | 0.041252691 |
| 19 | ACTN4 | 4.163476401 | 1.42850224 | 12.13476274 | 0.008965146 |
| 20 | ACTR3B | 0.188822765 | 0.060641548 | 0.587947334 | 0.004021748 |
| 21 | ACTR6 | 0.329634746 | 0.16609062 | 0.654215546 | 0.001507428 |
| 22 | ADAM32 | 0.644083663 | 0.483074344 | 0.8587576 | 0.002722494 |
| 23 | ADAMTS14 | 1.747626903 | 1.100454089 | 2.775399557 | 0.018001699 |
| 24 | ADAP2 | 1.997133967 | 1.014929824 | 3.929871788 | 0.045190797 |
| 25 | ADH7 | 0.479586529 | 0.264055457 | 0.871041414 | 0.015803804 |
| 26 | ADK | 0.309867087 | 0.141727431 | 0.677480785 | 0.003329309 |
| 27 | ADM | 1.781689458 | 1.162299726 | 2.73115209 | 0.008047901 |
| 28 | ADO | 0.33666661 | 0.115390835 | 0.982265239 | 0.046292391 |
| 29 | ADSS | 0.393650315 | 0.170304105 | 0.909905083 | 0.029196756 |
| 30 | AGAP1 | 3.922899206 | 1.073064346 | 14.34130044 | 0.038773317 |
| 31 | AGGF1 | 0.367953926 | 0.165973516 | 0.815733102 | 0.013840854 |
| 32 | AGK | 0.297582599 | 0.128510732 | 0.689089555 | 0.004666805 |
| 33 | AGPAT5 | 0.448348759 | 0.245179566 | 0.819875054 | 0.009190795 |
| 34 | AGRN | 2.594321076 | 1.249836465 | 5.385105998 | 0.010513361 |
| 35 | AGTPBP1 | 0.282526934 | 0.104754966 | 0.761982663 | 0.012526332 |
| 36 | AGXT | 1.901822046 | 1.025796766 | 3.52596851 | 0.041268125 |
| 37 | AHNAK | 2.179230731 | 1.030067204 | 4.610424019 | 0.041605224 |
| 38 | AHSA2 | 5.505120517 | 1.16414063 | 26.03323956 | 0.031421897 |
| 39 | AIDA | 0.220380728 | 0.0709835 | 0.68421063 | 0.008883866 |
| 40 | AIPL1 | 0.687517537 | 0.505971366 | 0.934203782 | 0.016618697 |
| 41 | AKAP13 | 3.679029181 | 1.414864855 | 9.566465422 | 0.007546134 |
| 42 | AKNAD1 | 0.45014208 | 0.254882671 | 0.794984967 | 0.005948735 |
| 43 | ALAS1 | 4.60381897 | 1.3955307 | 15.18787735 | 0.012168624 |
| 44 | ALDH16A1 | 2.473977948 | 1.006974223 | 6.078176332 | 0.048254736 |
| 45 | ALDOC | 2.117144204 | 1.261466019 | 3.55324639 | 0.004523036 |
| 46 | ALG2 | 0.307187274 | 0.11155154 | 0.845923066 | 0.022388037 |
| 47 | ALKBH5 | 3.777135897 | 1.350247626 | 10.56602901 | 0.011337853 |
| 48 | ALMS1P | 0.534964755 | 0.290387216 | 0.985536804 | 0.044781737 |
| 49 | AMBRA1 | 4.35234867 | 1.433732697 | 13.21232262 | 0.009435013 |
| 50 | AMN1 | 0.537713441 | 0.298925035 | 0.967251688 | 0.038348115 |
| 51 | ANAPC7 | 0.339500072 | 0.133522225 | 0.863229314 | 0.023276982 |
| 52 | ANGPTL4 | 1.492213208 | 1.106215746 | 2.012898718 | 0.008768069 |
| 53 | ANKIB1 | 0.443182742 | 0.227536303 | 0.863207057 | 0.016737296 |
| 54 | ANKRD11 | 3.02553791 | 1.110941693 | 8.239748044 | 0.03032794 |
| 55 | ANKRD22 | 0.581754965 | 0.425969738 | 0.79451381 | 0.000658149 |
| 56 | ANKRD26 | 0.653060005 | 0.427309659 | 0.998075661 | 0.048968646 |
| 57 | ANKRD32 | 0.40987186 | 0.16979114 | 0.989421132 | 0.047298574 |
| 58 | ANKRD34A | 1.576739856 | 1.075654386 | 2.311252207 | 0.019609969 |
| 59 | ANKRD50 | 0.747447848 | 0.564243354 | 0.990137112 | 0.042452714 |
| 60 | ANKS6 | 0.397758973 | 0.17433025 | 0.907543013 | 0.028490048 |
| 61 | ANLN | 0.456761698 | 0.284693797 | 0.732826816 | 0.001159338 |
| 62 | ANPEP | 1.585006641 | 1.219235845 | 2.060508688 | 0.000580046 |
| 63 | ANXA3 | 0.386370343 | 0.207411548 | 0.719738336 | 0.002734607 |
| 64 | AOAH | 0.516690429 | 0.315308749 | 0.846690745 | 0.008783036 |
| 65 | AOC3 | 1.842525288 | 1.1477142 | 2.957965874 | 0.011392949 |
| 66 | AP1AR | 0.506542182 | 0.26242221 | 0.977756349 | 0.042662022 |
| 67 | AP1M1 | 2.412748162 | 1.034504943 | 5.627187899 | 0.041502297 |
| 68 | APP | 2.745223963 | 1.242481907 | 6.065484386 | 0.012534106 |
| 69 | AQR | 0.495217405 | 0.256743765 | 0.955194681 | 0.036017368 |
| 70 | ARCN1 | 5.011716908 | 1.316075527 | 19.08500375 | 0.018149516 |
| 71 | ARHGAP1 | 4.296015996 | 1.64870069 | 11.19412005 | 0.002852478 |
| 72 | ARHGAP4 | 2.044579134 | 1.20672279 | 3.464179073 | 0.007850405 |
| 73 | ARHGDIA | 2.270981168 | 1.013214318 | 5.090093354 | 0.046388163 |
| 74 | ARHGEF17 | 1.55533573 | 1.084185803 | 2.231231238 | 0.016441031 |
| 75 | ARID3B | 3.591432423 | 1.347682614 | 9.570789676 | 0.010569282 |
| 76 | ARL5A | 0.241706022 | 0.109366163 | 0.534185343 | 0.000448721 |
| 77 | ARMC10 | 0.292589541 | 0.109940829 | 0.778679228 | 0.01386022 |
| 78 | ARMCX3-AS1 | 1.366884303 | 1.004841448 | 1.859370651 | 0.046510816 |
| 79 | ARSA | 1.964380481 | 1.129702338 | 3.415758775 | 0.016755716 |
| 80 | ASF1A | 0.440005814 | 0.243773654 | 0.794200331 | 0.006435966 |
| 81 | ASNSD1 | 0.149840361 | 0.036460075 | 0.615800542 | 0.008480793 |
| 82 | ASPHD1 | 2.391629497 | 1.45237444 | 3.938303712 | 0.000611475 |
| 83 | ASPM | 0.565521954 | 0.344506994 | 0.928326816 | 0.024192249 |
| 84 | ATG14 | 0.498944837 | 0.261670427 | 0.951372125 | 0.034742481 |
| 85 | ATG2A | 2.486077775 | 1.245973079 | 4.960446425 | 0.009768166 |
| 86 | ATG3 | 0.308476416 | 0.101683147 | 0.935825665 | 0.037792242 |
| 87 | ATG5 | 0.353877255 | 0.139825047 | 0.895612871 | 0.028331231 |
| 88 | ATP6V1E2 | 0.511025736 | 0.278783011 | 0.936740378 | 0.029906608 |
| 89 | ATP6V1G1 | 0.317113285 | 0.105360725 | 0.954443281 | 0.041062094 |
| 90 | ATP6V1G3 | 0.61607332 | 0.403997459 | 0.939477039 | 0.024451739 |
| 91 | ATR | 0.311389507 | 0.115813603 | 0.837236928 | 0.020777772 |
| 92 | AURKA | 0.456042296 | 0.254223575 | 0.818077458 | 0.008452597 |
| 93 | B3GALTL | 2.33099368 | 1.043605367 | 5.206500186 | 0.039011898 |
| 94 | B3GNT8 | 2.429094933 | 1.113234264 | 5.300323916 | 0.025785839 |
| 95 | B3GNTL1 | 0.517167216 | 0.279482246 | 0.956990769 | 0.035731556 |
| 96 | B4GALNT1 | 1.540515579 | 1.003593646 | 2.364690389 | 0.048112802 |
| 97 | BAG6 | 4.211737447 | 1.561082262 | 11.36309902 | 0.00451855 |
| 98 | BAHD1 | 4.626812908 | 1.408336813 | 15.20048151 | 0.011596896 |
| 99 | BBIP1 | 0.233344571 | 0.074135965 | 0.734457145 | 0.012864122 |
| 100 | BCAN | 0.674984184 | 0.500116302 | 0.910995397 | 0.010190981 |
| 101 | BCCIP | 0.461339142 | 0.257066646 | 0.827932396 | 0.009519503 |
| 102 | BCKDK | 3.717079643 | 1.268900093 | 10.8887068 | 0.016654351 |
| 103 | BCL2L11 | 3.359691294 | 1.174381344 | 9.611465349 | 0.023840306 |
| 104 | BCL2L2 | 6.394493122 | 1.625835772 | 25.14986015 | 0.007917241 |
| 105 | BDKRB2 | 2.526452822 | 1.14717504 | 5.564071428 | 0.021402002 |
| 106 | BET1L | 10.84516022 | 2.597642193 | 45.27856092 | 0.001078677 |
| 107 | BFSP1 | 0.667039373 | 0.453259638 | 0.981648238 | 0.039983477 |
| 108 | BMP2 | 2.000879107 | 1.143398012 | 3.501420466 | 0.015127111 |
| 109 | BRCA1 | 0.52008452 | 0.293972995 | 0.920111414 | 0.024703662 |
| 110 | BRCA2 | 0.605776179 | 0.378289641 | 0.970062985 | 0.036934864 |
| 111 | BRIP1 | 0.579089297 | 0.342349922 | 0.979536996 | 0.041644158 |
| 112 | BRMS1L | 0.577838956 | 0.339468612 | 0.983589784 | 0.0432867 |
| 113 | BSN | 1.612184045 | 1.123930474 | 2.312542862 | 0.009467332 |
| 114 | BTG4 | 0.717931322 | 0.517051111 | 0.996855768 | 0.047842062 |
| 115 | BUB1 | 0.369584827 | 0.179341376 | 0.761636538 | 0.006975612 |
| 116 | C10orf88 | 0.276498784 | 0.077487743 | 0.986628011 | 0.047624273 |
| 117 | C10orf90 | 0.668273137 | 0.452513612 | 0.986907297 | 0.042742545 |
| 118 | C11orf35 | 2.664960898 | 1.270465158 | 5.590091581 | 0.009505871 |
| 119 | C11orf75 | 1.994391763 | 1.060636464 | 3.75019966 | 0.032138577 |
| 120 | C12orf11 | 0.245822056 | 0.086825473 | 0.695976439 | 0.008228533 |
| 121 | C12orf29 | 0.2036711 | 0.089796517 | 0.461954634 | 0.000139964 |
| 122 | C12orf71 | 0.68118969 | 0.505106158 | 0.918657171 | 0.011870319 |
| 123 | C12orf73 | 0.489664513 | 0.250387242 | 0.957602044 | 0.036927649 |
| 124 | C14orf101 | 0.344653684 | 0.159035856 | 0.746914341 | 0.006945401 |
| 125 | C14orf109 | 0.274692835 | 0.098517066 | 0.765919616 | 0.013523231 |
| 126 | C14orf126 | 0.361037486 | 0.143623343 | 0.907568805 | 0.03029731 |
| 127 | C14orf182 | 0.597164246 | 0.357990441 | 0.99613033 | 0.04828913 |
| 128 | C14orf23 | 0.575289808 | 0.354342163 | 0.934007852 | 0.0253467 |
| 129 | C14orf81 | 0.738991111 | 0.552446659 | 0.988525956 | 0.04157889 |
| 130 | C14orf82 | 1.565971808 | 1.047720652 | 2.340573985 | 0.028719967 |
| 131 | C15orf43 | 0.727758079 | 0.549120777 | 0.964508799 | 0.027005971 |
| 132 | C15orf57 | 3.143290849 | 1.106884705 | 8.926202806 | 0.031503049 |
| 133 | C16orf58 | 3.739199433 | 1.293032314 | 10.81304175 | 0.014920518 |
| 134 | C16orf72 | 0.515252954 | 0.272442632 | 0.974464258 | 0.041397566 |
| 135 | C17orf42 | 0.265071951 | 0.09818884 | 0.715591909 | 0.008782465 |
| 136 | C17orf59 | 2.882239436 | 1.037629064 | 8.006044213 | 0.042272335 |
| 137 | C17orf78 | 1.211011439 | 1.009369112 | 1.452935985 | 0.03936809 |
| 138 | C17orf97 | 0.615658537 | 0.424347222 | 0.89322002 | 0.010628066 |
| 139 | C19orf23 | 0.43260838 | 0.205077073 | 0.912583781 | 0.027796034 |
| 140 | C19orf29-AS1 | 0.629260498 | 0.402620011 | 0.983480114 | 0.042044736 |
| 141 | C19orf67 | 0.667729465 | 0.447809233 | 0.995653073 | 0.047553624 |
| 142 | C19orf69 | 1.611891909 | 1.012750723 | 2.565483753 | 0.044072746 |
| 143 | C1orf124 | 0.234577078 | 0.087599022 | 0.628162326 | 0.003912565 |
| 144 | C1orf131 | 0.356184618 | 0.196129725 | 0.646854942 | 0.00069653 |
| 145 | C1orf180 | 0.742391549 | 0.602552761 | 0.914683739 | 0.005150858 |
| 146 | C1orf31 | 0.230184958 | 0.096228099 | 0.550619989 | 0.000963682 |
| 147 | C1orf55 | 0.383327619 | 0.166245111 | 0.883875996 | 0.024476984 |
| 148 | C22orf23 | 0.68585379 | 0.473362043 | 0.99373287 | 0.046240155 |
| 149 | C2orf15 | 0.28213241 | 0.127779373 | 0.622938547 | 0.001741213 |
| 150 | C2orf57 | 0.739968455 | 0.560792772 | 0.976391534 | 0.033266071 |
| 151 | C2orf69 | 0.355008084 | 0.186893447 | 0.674345417 | 0.001558343 |
| 152 | C2orf70 | 0.65030636 | 0.442482524 | 0.95574026 | 0.028495595 |
| 153 | C2orf76 | 0.209538123 | 0.056930432 | 0.771225925 | 0.018738562 |
| 154 | C3orf26 | 0.365992581 | 0.1651344 | 0.811160903 | 0.013309306 |
| 155 | C3orf55 | 0.83136104 | 0.7003441 | 0.986887987 | 0.034788595 |
| 156 | C4orf21 | 0.513730921 | 0.269553637 | 0.979098121 | 0.042953843 |
| 157 | C5orf44 | 0.244216271 | 0.091228028 | 0.653763852 | 0.005017372 |
| 158 | C5orf52 | 0.582372018 | 0.393118247 | 0.862735753 | 0.00701128 |
| 159 | C6orf97 | 0.712161158 | 0.523149131 | 0.969462597 | 0.031002686 |
| 160 | C7orf11 | 0.388912125 | 0.162230629 | 0.932330976 | 0.03425717 |
| 161 | C7orf25 | 0.261802337 | 0.110693034 | 0.619194008 | 0.002278272 |
| 162 | C7orf29 | 0.449774291 | 0.221090301 | 0.914996779 | 0.027444757 |
| 163 | C7orf44 | 0.273889941 | 0.101148488 | 0.741639361 | 0.010832575 |
| 164 | C7orf63 | 0.534031035 | 0.285515688 | 0.998856311 | 0.049582771 |
| 165 | C8orf29 | 0.717992334 | 0.526544349 | 0.979049519 | 0.036279882 |
| 166 | C8orf40 | 0.395293235 | 0.213935433 | 0.730392058 | 0.003047343 |
| 167 | C8orf42 | 1.609069443 | 1.013553441 | 2.554482443 | 0.043690057 |
| 168 | C8orf45 | 0.721796027 | 0.584330038 | 0.891601443 | 0.002491732 |
| 169 | C8orf56 | 2.116885796 | 1.013838517 | 4.420038693 | 0.045873746 |
| 170 | C9orf114 | 0.238065393 | 0.083678598 | 0.677295421 | 0.007137066 |
| 171 | C9orf116 | 0.382036364 | 0.227182674 | 0.642442406 | 0.000285072 |
| 172 | C9orf156 | 0.258558108 | 0.089791845 | 0.744525246 | 0.012187593 |
| 173 | C9orf169 | 0.644428056 | 0.418963024 | 0.991227138 | 0.045492332 |
| 174 | C9orf4 | 0.75965628 | 0.596676252 | 0.967153732 | 0.025679578 |
| 175 | C9orf57 | 0.776294279 | 0.621955768 | 0.968931937 | 0.025154046 |
| 176 | C9orf78 | 0.204185438 | 0.064225108 | 0.649149445 | 0.007099045 |
| 177 | C9orf82 | 0.302628904 | 0.135493203 | 0.675932455 | 0.003554188 |
| 178 | CA11 | 2.317680451 | 1.042113278 | 5.154566964 | 0.039292292 |
| 179 | CAMK2N1 | 1.852405195 | 1.043775979 | 3.287491833 | 0.035173725 |
| 180 | CAMK2N2 | 1.729503658 | 1.108422247 | 2.698595152 | 0.015802345 |
| 181 | CAMKK2 | 2.950992352 | 1.039611651 | 8.376547 | 0.042058225 |
| 182 | CAP1 | 3.767827635 | 1.072560687 | 13.23610427 | 0.038524047 |
| 183 | CAPS2 | 0.715500934 | 0.51767748 | 0.988919948 | 0.042617304 |
| 184 | CAPZA2 | 0.246685451 | 0.087454015 | 0.695836684 | 0.008160274 |
| 185 | CARM1 | 2.569937012 | 1.042329451 | 6.336361539 | 0.040363813 |
| 186 | CASC1 | 0.686856367 | 0.515428209 | 0.915300446 | 0.010344513 |
| 187 | CASC2 | 0.569260331 | 0.336750387 | 0.96230721 | 0.035430568 |
| 188 | CBFB | 0.404716363 | 0.176367551 | 0.928715817 | 0.032804654 |
| 189 | CBLL1 | 0.246882567 | 0.072397938 | 0.841888644 | 0.025421189 |
| 190 | CBLN1 | 1.474953017 | 1.188433309 | 1.83054984 | 0.000421064 |
| 191 | CBX3 | 0.299657545 | 0.11118182 | 0.807637839 | 0.017205121 |
| 192 | CBX3P2 | 0.570735302 | 0.340326491 | 0.957136144 | 0.033499821 |
| 193 | CCDC107 | 2.441138481 | 1.097357637 | 5.4304603 | 0.028691256 |
| 194 | CCDC112 | 0.533520965 | 0.316135835 | 0.900387077 | 0.01862513 |
| 195 | CCDC113 | 0.538758237 | 0.368652832 | 0.787354423 | 0.001398373 |
| 196 | CCDC138 | 0.39049617 | 0.208497136 | 0.731363803 | 0.003312673 |
| 197 | CCDC144A | 1.693545896 | 1.082479904 | 2.649562075 | 0.02105316 |
| 198 | CCDC151 | 1.735402256 | 1.050485257 | 2.866885536 | 0.031376499 |
| 199 | CCDC160 | 0.741887294 | 0.567560075 | 0.969759469 | 0.028913777 |
| 200 | CCDC25 | 0.431030823 | 0.233420965 | 0.79593352 | 0.007159737 |
| 201 | CCDC43 | 0.381688389 | 0.157297567 | 0.92618105 | 0.033211691 |
| 202 | CCDC53 | 0.334991771 | 0.145056882 | 0.773624014 | 0.010436869 |
| 203 | CCDC59 | 0.30588079 | 0.12645364 | 0.739900074 | 0.008579411 |
| 204 | CCDC96 | 3.235445931 | 1.051658164 | 9.953909671 | 0.040578753 |
| 205 | CCDC99 | 0.380797793 | 0.156716236 | 0.925283577 | 0.033056694 |
| 206 | CCNB1 | 0.336853458 | 0.14027585 | 0.808907962 | 0.014915266 |
| 207 | CCNH | 0.354686406 | 0.143691532 | 0.875503548 | 0.024552925 |
| 208 | CCR10 | 1.591957319 | 1.047189101 | 2.420124601 | 0.029575742 |
| 209 | CCT6B | 0.512715678 | 0.279675768 | 0.939936157 | 0.030751377 |
| 210 | CD14 | 1.876216835 | 1.031430776 | 3.412918919 | 0.039270526 |
| 211 | CD160 | 0.691674369 | 0.478949252 | 0.998881259 | 0.049306293 |
| 212 | CD1B | 0.623817168 | 0.449322195 | 0.866077537 | 0.004820058 |
| 213 | CD2 | 0.466936194 | 0.234108832 | 0.931316464 | 0.030620801 |
| 214 | CD82 | 2.495367694 | 1.261167426 | 4.937377701 | 0.008628926 |
| 215 | CD99 | 2.02585524 | 1.153000084 | 3.559487559 | 0.014087046 |
| 216 | CDC123 | 0.07114483 | 0.017412467 | 0.290687527 | 0.000232881 |
| 217 | CDC25C | 0.490664003 | 0.248110227 | 0.970339542 | 0.040706444 |
| 218 | CDC26 | 0.223435754 | 0.072027625 | 0.693116516 | 0.009470501 |
| 219 | CDCA2 | 0.510579095 | 0.308031859 | 0.846311851 | 0.009129749 |
| 220 | CDCA7 | 0.390217517 | 0.182095931 | 0.836206004 | 0.015522022 |
| 221 | CDCP1 | 2.518446391 | 1.060946729 | 5.978219314 | 0.03625141 |
| 222 | CDHR5 | 1.469283609 | 1.048667011 | 2.058608025 | 0.025343492 |
| 223 | CDK1 | 0.395305834 | 0.185016621 | 0.844608995 | 0.016577654 |
| 224 | CDK11A | 3.002239779 | 1.067410736 | 8.444213077 | 0.037196119 |
| 225 | CDK11B | 3.02486247 | 1.092562928 | 8.374614151 | 0.033142934 |
| 226 | CDK5 | 0.319609494 | 0.122475423 | 0.834046753 | 0.019765864 |
| 227 | CDKN2A | 0.577578566 | 0.33520366 | 0.995206914 | 0.048011004 |
| 228 | CDKN3 | 0.387927275 | 0.205806964 | 0.73120738 | 0.00341211 |
| 229 | CDY2A | 1.410401635 | 1.017926356 | 1.95420107 | 0.038757676 |
| 230 | CEACAM6 | 0.374302618 | 0.217405021 | 0.64443061 | 0.000392529 |
| 231 | CEBPZ | 0.430274435 | 0.224266267 | 0.825519112 | 0.011189586 |
| 232 | CENPA | 0.429492391 | 0.230152238 | 0.801485642 | 0.007926709 |
| 233 | CENPE | 0.591809145 | 0.357263046 | 0.980336669 | 0.041641629 |
| 234 | CENPF | 0.550200525 | 0.336340019 | 0.900043409 | 0.017342896 |
| 235 | CENPH | 0.377280061 | 0.201617053 | 0.705993079 | 0.002296631 |
| 236 | CENPN | 0.303606376 | 0.123609075 | 0.745712495 | 0.009324142 |
| 237 | CENPP | 0.318410894 | 0.110419826 | 0.918182008 | 0.034180401 |
| 238 | CENPQ | 0.253112031 | 0.100416541 | 0.637999473 | 0.003582773 |
| 239 | CEP55 | 0.333940817 | 0.154502157 | 0.721779366 | 0.005286384 |
| 240 | CEP78 | 0.185275377 | 0.062890466 | 0.545821451 | 0.00222603 |
| 241 | CETN2 | 0.364561796 | 0.15684854 | 0.847348038 | 0.019032496 |
| 242 | CFTR | 0.537362215 | 0.318885057 | 0.905524243 | 0.019663891 |
| 243 | CHCHD3 | 0.325473813 | 0.12390551 | 0.854951505 | 0.022726598 |
| 244 | CHST1 | 1.613620275 | 1.066820919 | 2.44068179 | 0.023430712 |
| 245 | CITED2 | 4.000487013 | 1.242824343 | 12.87703804 | 0.020102546 |
| 246 | CKAP2 | 0.437787405 | 0.208053992 | 0.921192672 | 0.029538298 |
| 247 | CKS2 | 0.450088222 | 0.231357775 | 0.875610978 | 0.018713762 |
| 248 | CLDN20 | 0.720806405 | 0.556329015 | 0.933911156 | 0.013235919 |
| 249 | CLDN5 | 1.601878206 | 1.000363468 | 2.56508146 | 0.049823428 |
| 250 | CLDND2 | 0.655325057 | 0.441942783 | 0.971734232 | 0.035499345 |
| 251 | CLK3 | 2.786906009 | 1.208129687 | 6.428817358 | 0.01624727 |
| 252 | CLPTM1L | 2.476763038 | 1.148805691 | 5.339767373 | 0.020674086 |
| 253 | CLSTN1 | 3.076255787 | 1.313413687 | 7.205155358 | 0.009658996 |
| 254 | CMIP | 3.162515287 | 1.217902677 | 8.212070741 | 0.018036822 |
| 255 | CNN1 | 1.431974453 | 1.008542793 | 2.033181783 | 0.044693777 |
| 256 | CNOT7 | 0.38069866 | 0.182327329 | 0.794897126 | 0.010138637 |
| 257 | COA5 | 0.151425677 | 0.033016958 | 0.694483597 | 0.015135036 |
| 258 | COG2 | 0.31821953 | 0.112803206 | 0.897702052 | 0.030471124 |
| 259 | COL28A1 | 0.534399512 | 0.305895067 | 0.933597399 | 0.027711325 |
| 260 | COL4A2 | 1.804662522 | 1.1403087 | 2.856074691 | 0.011717845 |
| 261 | COLEC10 | 0.665744556 | 0.461775971 | 0.959807011 | 0.029275955 |
| 262 | COMMD8 | 0.475101134 | 0.23160957 | 0.974575827 | 0.042334508 |
| 263 | COPG2 | 0.325403353 | 0.128682577 | 0.822856871 | 0.017697906 |
| 264 | COQ3 | 0.421209392 | 0.212253651 | 0.835874206 | 0.013411082 |
| 265 | COX18 | 0.513345175 | 0.295442221 | 0.891962115 | 0.018002463 |
| 266 | CPNE9 | 0.602652912 | 0.412938639 | 0.879526635 | 0.00865201 |
| 267 | CPSF3 | 0.234152553 | 0.07878958 | 0.695871441 | 0.008990033 |
| 268 | CPT1A | 3.125305887 | 1.53320497 | 6.370666075 | 0.00171211 |
| 269 | CPXM2 | 1.459741959 | 1.046469906 | 2.036223474 | 0.025917778 |
| 270 | CREB3 | 2.984538813 | 1.173712803 | 7.58914098 | 0.021656402 |
| 271 | CRELD1 | 3.195682064 | 1.15999917 | 8.803785486 | 0.024638795 |
| 272 | CROT | 0.386036641 | 0.161231377 | 0.92428838 | 0.032622078 |
| 273 | CRY2 | 18.42207847 | 4.54007052 | 74.75059555 | 4.56E-05 |
| 274 | CRYAB | 1.749906294 | 1.039046162 | 2.947099131 | 0.03537967 |
| 275 | CRYBA2 | 1.735156423 | 1.066444211 | 2.823183606 | 0.026487558 |
| 276 | CSNK1D | 3.892657183 | 1.372715678 | 11.0385422 | 0.010598532 |
| 277 | CSPG4 | 1.591398937 | 1.069029922 | 2.369017485 | 0.022091053 |
| 278 | CTC1 | 3.335119647 | 1.006870678 | 11.04712184 | 0.048704509 |
| 279 | CTDSP1 | 3.172452775 | 1.072666802 | 9.382649484 | 0.036910238 |
| 280 | CTNND1 | 3.0815624 | 1.024656114 | 9.267525657 | 0.045142901 |
| 281 | CTSB | 2.208858344 | 1.011114176 | 4.825424566 | 0.046846343 |
| 282 | CTSD | 2.424356404 | 1.405505206 | 4.181773182 | 0.001453852 |
| 283 | CTTNBP2 | 0.646560441 | 0.419375168 | 0.996817256 | 0.048336632 |
| 284 | CUL1 | 0.484950603 | 0.248748246 | 0.945442194 | 0.033614126 |
| 285 | CUL2 | 0.308904629 | 0.140371251 | 0.679783569 | 0.003510468 |
| 286 | CUL3 | 0.429282197 | 0.199241162 | 0.924925366 | 0.030831958 |
| 287 | CUL5 | 0.31145845 | 0.101790696 | 0.95299836 | 0.040920404 |
| 288 | CUZD1 | 0.618881009 | 0.418218775 | 0.915821398 | 0.016407245 |
| 289 | CXCL11 | 0.781967921 | 0.621312167 | 0.984165227 | 0.036082825 |
| 290 | CXXC1 | 5.952363007 | 1.958620047 | 18.08958579 | 0.001659123 |
| 291 | CYB5B | 0.546795757 | 0.300820012 | 0.993901959 | 0.047700568 |
| 292 | CYB5R3 | 2.587732266 | 1.229403946 | 5.446833239 | 0.012284927 |
| 293 | CYBRD1 | 1.689516954 | 1.002995219 | 2.845943314 | 0.048700411 |
| 294 | CYP1A1 | 0.717602933 | 0.53569821 | 0.961276257 | 0.026098774 |
| 295 | CYP3A43 | 0.684787885 | 0.473094858 | 0.99120597 | 0.044773371 |
| 296 | CYTL1 | 1.478904674 | 1.063223778 | 2.057101319 | 0.020121227 |
| 297 | DACT3 | 1.482681612 | 1.011777342 | 2.172755478 | 0.043381785 |
| 298 | DBF4 | 0.338412484 | 0.190657936 | 0.600672657 | 0.000214716 |
| 299 | DCAF13 | 0.416186476 | 0.178737208 | 0.969082959 | 0.042073862 |
| 300 | DCK | 0.428104126 | 0.185144154 | 0.989894303 | 0.047287792 |
| 301 | DCLRE1A | 0.352713749 | 0.125364139 | 0.992365043 | 0.048326553 |
| 302 | DDIT4L | 1.262347026 | 1.011456553 | 1.575470551 | 0.039329272 |
| 303 | DDX21 | 0.326465663 | 0.143069384 | 0.744952035 | 0.007826543 |
| 304 | DDX31 | 0.265983572 | 0.105266391 | 0.672078333 | 0.005107052 |
| 305 | DDX5 | 0.235693159 | 0.07096482 | 0.782800059 | 0.01828437 |
| 306 | DEDD | 0.385932208 | 0.168055612 | 0.886276079 | 0.024795121 |
| 307 | DEFA8P | 0.566163205 | 0.337855476 | 0.948751159 | 0.030796701 |
| 308 | DEFB103B | 0.699825559 | 0.534714877 | 0.915919557 | 0.009331952 |
| 309 | DEK | 0.38042965 | 0.167703413 | 0.862992088 | 0.020747695 |
| 310 | DENND5A | 1.848789986 | 1.025603423 | 3.332695988 | 0.0409482 |
| 311 | DEPDC1 | 0.544028703 | 0.325978801 | 0.907933979 | 0.019828949 |
| 312 | DEPDC1B | 0.484381147 | 0.251157394 | 0.934175544 | 0.030529091 |
| 313 | DGCR2 | 2.664707028 | 1.189466513 | 5.96962039 | 0.017238986 |
| 314 | DGKD | 2.783561088 | 1.044682323 | 7.416811947 | 0.040620157 |
| 315 | DHX29 | 0.42592304 | 0.197466527 | 0.918689558 | 0.029540277 |
| 316 | DIAPH1 | 3.629868129 | 1.164946978 | 11.31033677 | 0.026198437 |
| 317 | DIEXF | 0.256262287 | 0.111351077 | 0.589759539 | 0.001366553 |
| 318 | DIRAS2 | 1.301545823 | 1.003096468 | 1.688792239 | 0.047341873 |
| 319 | DKC1 | 0.337259927 | 0.130871541 | 0.869129052 | 0.024425304 |
| 320 | DKFZP564C196 | 0.499140917 | 0.255046517 | 0.976847904 | 0.042525521 |
| 321 | DLD | 0.268330681 | 0.10173279 | 0.707749729 | 0.007848911 |
| 322 | DLGAP5 | 0.414446844 | 0.232253033 | 0.739564878 | 0.002872953 |
| 323 | DNAAF2 | 0.21953966 | 0.056878975 | 0.847372209 | 0.027785598 |
| 324 | DNAH12 | 0.578555034 | 0.339779215 | 0.985127735 | 0.04388973 |
| 325 | DNAH14 | 0.647117957 | 0.47983983 | 0.872711319 | 0.004341622 |
| 326 | DNAH7 | 0.704828587 | 0.497704598 | 0.998148981 | 0.048792512 |
| 327 | DNAJB2 | 3.00581538 | 1.120656816 | 8.062170299 | 0.028796745 |
| 328 | DNAJB6 | 0.165770395 | 0.045205103 | 0.607892069 | 0.006712795 |
| 329 | DNAJC13 | 0.318871859 | 0.114250705 | 0.889966167 | 0.029067844 |
| 330 | DNAJC2 | 0.469471611 | 0.26600605 | 0.828566094 | 0.009086418 |
| 331 | DNAJC25 | 0.451431534 | 0.224663676 | 0.907091137 | 0.025493154 |
| 332 | DNER | 1.265582261 | 1.016131047 | 1.576271548 | 0.035480391 |
| 333 | DNM2 | 3.240052961 | 1.502119173 | 6.988755207 | 0.002723237 |
| 334 | DNTTIP2 | 0.464754936 | 0.222423441 | 0.971107852 | 0.04155606 |
| 335 | DOCK11 | 0.425576447 | 0.235987921 | 0.767477042 | 0.004516813 |
| 336 | DOLK | 0.282459614 | 0.092518533 | 0.862350833 | 0.026417307 |
| 337 | DPM1 | 0.478857 | 0.230453904 | 0.995010378 | 0.048453508 |
| 338 | DPY30 | 0.33721686 | 0.116291141 | 0.977849299 | 0.045370684 |
| 339 | DTL | 0.477204324 | 0.23503369 | 0.968899257 | 0.040618719 |
| 340 | DTNB | 0.618028808 | 0.389101589 | 0.981644945 | 0.041506149 |
| 341 | DUS4L | 0.396411131 | 0.20356083 | 0.771964748 | 0.006506993 |
| 342 | E2F5 | 0.449958922 | 0.226395053 | 0.894290882 | 0.022681125 |
| 343 | ECT2 | 0.559602988 | 0.338137459 | 0.926118935 | 0.023909888 |
| 344 | EDEM1 | 3.100520553 | 1.143626188 | 8.40591777 | 0.026169051 |
| 345 | EDIL3 | 1.892231756 | 1.077859532 | 3.32189948 | 0.026345337 |
| 346 | EDN2 | 1.418356531 | 1.066099464 | 1.887005215 | 0.01642273 |
| 347 | EEF1E1 | 0.399181139 | 0.165957325 | 0.960159981 | 0.040291126 |
| 348 | EEPD1 | 0.592130285 | 0.35687432 | 0.982469891 | 0.042517224 |
| 349 | EFCAB10 | 0.582028202 | 0.366125501 | 0.925247839 | 0.022110144 |
| 350 | EFCAB11 | 0.506388543 | 0.281006368 | 0.912539306 | 0.023539779 |
| 351 | EFCAB2 | 0.566249065 | 0.361184238 | 0.887740852 | 0.013175038 |
| 352 | EFHD2 | 2.223881335 | 1.065093721 | 4.643392498 | 0.033349004 |
| 353 | EHBP1L1 | 2.646003687 | 1.115219388 | 6.277989414 | 0.027290023 |
| 354 | EHD1 | 2.077203155 | 1.184150574 | 3.643770515 | 0.010789567 |
| 355 | EHD2 | 1.549576899 | 1.004221409 | 2.391094777 | 0.047816226 |
| 356 | EIF2S1 | 0.23075434 | 0.086446867 | 0.615957145 | 0.003419169 |
| 357 | EIF2S2 | 0.369124526 | 0.1703799 | 0.799700642 | 0.011516469 |
| 358 | EIF5A | 2.436063848 | 1.141843373 | 5.197216368 | 0.021275473 |
| 359 | EMILIN1 | 1.957497798 | 1.065790928 | 3.595262006 | 0.030358503 |
| 360 | EML3 | 2.285572756 | 1.001523777 | 5.215894964 | 0.049578717 |
| 361 | ENC1 | 0.505788466 | 0.300762041 | 0.85057932 | 0.010164223 |
| 362 | ENOPH1 | 0.373822005 | 0.149857607 | 0.932504486 | 0.034875527 |
| 363 | ENOX2 | 0.262023906 | 0.107475131 | 0.638813154 | 0.003223649 |
| 364 | ENPP5 | 0.662888333 | 0.496658007 | 0.884755578 | 0.005251096 |
| 365 | EPAS1 | 3.886383629 | 1.61202696 | 9.369556518 | 0.002498969 |
| 366 | EPN2 | 6.504639204 | 1.555352252 | 27.20305392 | 0.010317033 |
| 367 | EPO | 0.685523616 | 0.522946877 | 0.89864315 | 0.006262202 |
| 368 | EPS8L2 | 2.715329639 | 1.226346978 | 6.012176962 | 0.013775059 |
| 369 | ERCC8 | 0.222657289 | 0.080161805 | 0.618452497 | 0.00395293 |
| 370 | ERGIC2 | 0.313127808 | 0.108091956 | 0.907088995 | 0.032382603 |
| 371 | ERI1 | 0.468969927 | 0.270537036 | 0.812948927 | 0.006980791 |
| 372 | ERICH1 | 0.541541621 | 0.313554646 | 0.935298936 | 0.027815542 |
| 373 | ERP27 | 0.774850245 | 0.609268835 | 0.985431828 | 0.037561702 |
| 374 | ESCO2 | 0.555842152 | 0.341975808 | 0.903457175 | 0.017806344 |
| 375 | ESF1 | 0.553423233 | 0.311635778 | 0.98280524 | 0.043470835 |
| 376 | ETAA1 | 0.405485719 | 0.19383534 | 0.848238861 | 0.016528072 |
| 377 | EXO1 | 0.559774575 | 0.322917433 | 0.970364379 | 0.038720744 |
| 378 | EXOC3 | 5.104162408 | 2.028913386 | 12.84060427 | 0.000534102 |
| 379 | EXOSC2 | 0.328582185 | 0.141049724 | 0.765448166 | 0.009895689 |
| 380 | EXT1 | 5.972886386 | 2.22005619 | 16.06958055 | 0.000401083 |
| 381 | EXT2 | 5.396873803 | 1.592957775 | 18.28438098 | 0.006772838 |
| 382 | EZH2 | 0.429309538 | 0.217996361 | 0.845457596 | 0.01446599 |
| 383 | FAAH2 | 0.546388303 | 0.318198392 | 0.938220259 | 0.028441458 |
| 384 | FAHD1 | 0.548932201 | 0.301800251 | 0.99843045 | 0.049401453 |
| 385 | FAM101B | 1.675800658 | 1.010884352 | 2.778070349 | 0.045291132 |
| 386 | FAM104B | 0.576380763 | 0.354295229 | 0.937677835 | 0.026477685 |
| 387 | FAM114A1 | 2.801893562 | 1.116880771 | 7.029047092 | 0.028126319 |
| 388 | FAM136A | 0.220609831 | 0.066648036 | 0.730234539 | 0.013332677 |
| 389 | FAM160A2 | 3.356275912 | 1.539224223 | 7.318354165 | 0.002332299 |
| 390 | FAM175B | 0.330844754 | 0.131420477 | 0.832885821 | 0.018866658 |
| 391 | FAM179B | 0.492097392 | 0.281671506 | 0.859724318 | 0.012741486 |
| 392 | FAM185A | 0.179995808 | 0.059630197 | 0.54332356 | 0.002348183 |
| 393 | FAM188A | 0.265538456 | 0.089549003 | 0.787397618 | 0.016804677 |
| 394 | FAM188B | 0.249488249 | 0.101098399 | 0.615681228 | 0.002592386 |
| 395 | FAM196B | 0.684070913 | 0.475165697 | 0.9848207 | 0.041128336 |
| 396 | FAM200A | 0.461765519 | 0.221509564 | 0.962610328 | 0.039242384 |
| 397 | FAM20B | 0.206368408 | 0.049897647 | 0.853505582 | 0.029357762 |
| 398 | FAM20C | 2.182176936 | 1.241477733 | 3.83566781 | 0.006695694 |
| 399 | FAM35A | 0.246603411 | 0.075740446 | 0.802916353 | 0.020103343 |
| 400 | FAM54A | 0.47039114 | 0.238854318 | 0.926371465 | 0.029172558 |
| 401 | FAM63A | 2.431704632 | 1.1237537 | 5.261995952 | 0.02405745 |
| 402 | FAM65A | 1.866039777 | 1.003748655 | 3.469099991 | 0.048633501 |
| 403 | FAM92A1 | 0.668887832 | 0.481712975 | 0.92879153 | 0.016349458 |
| 404 | FAM92A3 | 0.660697752 | 0.462590248 | 0.943646177 | 0.022673099 |
| 405 | FAM98A | 0.373476953 | 0.154756374 | 0.901320126 | 0.028444702 |
| 406 | FANCL | 0.175228061 | 0.045595997 | 0.673411599 | 0.011225336 |
| 407 | FANCM | 0.504450288 | 0.283634679 | 0.897175528 | 0.019842596 |
| 408 | FBLIM1 | 2.560669183 | 1.020424147 | 6.425785479 | 0.045173557 |
| 409 | FBXL13 | 0.305990961 | 0.163423992 | 0.572929761 | 0.000215165 |
| 410 | FBXO28 | 0.33910006 | 0.126267253 | 0.910678324 | 0.031905204 |
| 411 | FBXO32 | 1.734643229 | 1.01468971 | 2.965425885 | 0.044086587 |
| 412 | FBXO42 | 15.50011098 | 3.121487898 | 76.96760272 | 0.000801837 |
| 413 | FBXO5 | 0.461294784 | 0.213260631 | 0.997806661 | 0.049351536 |
| 414 | FBXW11 | 5.445394656 | 1.206185081 | 24.58355971 | 0.02754383 |
| 415 | FCGR2B | 1.745837308 | 1.01216674 | 3.01131008 | 0.045129705 |
| 416 | FHL3 | 1.885691987 | 1.001414615 | 3.550811236 | 0.049490468 |
| 417 | FIGNL1 | 0.479617804 | 0.235644688 | 0.976186815 | 0.042720357 |
| 418 | FKBP3 | 0.390499735 | 0.161364139 | 0.945005773 | 0.037032178 |
| 419 | FKRP | 5.173351831 | 1.598110656 | 16.74700626 | 0.006103189 |
| 420 | FKTN | 0.345442909 | 0.158470086 | 0.75301785 | 0.007508018 |
| 421 | FLII | 2.640385918 | 1.037880467 | 6.717187592 | 0.041549185 |
| 422 | FLJ32742 | 0.70911453 | 0.505441654 | 0.994859471 | 0.046613223 |
| 423 | FLJ39095 | 0.835437295 | 0.701682557 | 0.994688363 | 0.043405016 |
| 424 | FLJ40288 | 0.558823784 | 0.348662069 | 0.895663878 | 0.015615282 |
| 425 | FLJ42842 | 0.486707408 | 0.274042022 | 0.864407946 | 0.014003557 |
| 426 | FLJ44313 | 0.69690276 | 0.489954876 | 0.991261606 | 0.044559647 |
| 427 | FLNB | 2.596443879 | 1.135380501 | 5.937675354 | 0.023771017 |
| 428 | FNTA | 0.291635393 | 0.104782072 | 0.811696129 | 0.018302855 |
| 429 | FOXC2 | 1.497180355 | 1.125635311 | 1.991363449 | 0.005551227 |
| 430 | FOXD1 | 1.190581061 | 1.002327857 | 1.414191229 | 0.046984322 |
| 431 | FOXG1 | 0.781971928 | 0.632391758 | 0.966932424 | 0.023183764 |
| 432 | FOXL1 | 2.184893838 | 1.035182299 | 4.611517304 | 0.040297231 |
| 433 | FOXR1 | 0.754951081 | 0.583798101 | 0.97628124 | 0.032116234 |
| 434 | FRMPD4 | 0.627108888 | 0.4226001 | 0.930585575 | 0.020492373 |
| 435 | FUBP3 | 0.210858429 | 0.047546893 | 0.935103741 | 0.040534861 |
| 436 | FUT10 | 0.466733948 | 0.220901769 | 0.986142301 | 0.045876605 |
| 437 | FYTTD1 | 0.218897891 | 0.062305164 | 0.769058028 | 0.017809944 |
| 438 | FZD3 | 0.672033791 | 0.46696562 | 0.967157747 | 0.032375254 |
| 439 | FZD6 | 0.507007272 | 0.28065816 | 0.915905573 | 0.024379971 |
| 440 | GADD45A | 2.202959466 | 1.059827826 | 4.579074346 | 0.034377858 |
| 441 | GAGE7 | 0.763861122 | 0.594145537 | 0.982055367 | 0.035622357 |
| 442 | GALNS | 3.221641225 | 1.197323811 | 8.668475546 | 0.020527254 |
| 443 | GALNT11 | 0.179525005 | 0.054586406 | 0.590425895 | 0.004692538 |
| 444 | GALNTL5 | 1.378394585 | 1.009430004 | 1.88222227 | 0.043485831 |
| 445 | GANAB | 4.035027965 | 1.552436739 | 10.48767416 | 0.004203804 |
| 446 | GARNL3 | 0.53068663 | 0.337633615 | 0.834123992 | 0.006031402 |
| 447 | GAS2L1 | 2.103219352 | 1.046923825 | 4.225266004 | 0.03672589 |
| 448 | GATA1 | 0.641775615 | 0.439560317 | 0.937018026 | 0.02162704 |
| 449 | GATS | 0.402641872 | 0.170487289 | 0.950924129 | 0.038011678 |
| 450 | GBE1 | 2.456798382 | 1.010726344 | 5.971802683 | 0.047310747 |
| 451 | GBF1 | 6.228137553 | 1.66716727 | 23.26682996 | 0.006526725 |
| 452 | GDI1 | 3.064745489 | 1.262272934 | 7.441072894 | 0.013338761 |
| 453 | GDPD1 | 0.657815513 | 0.460764369 | 0.93913783 | 0.021131013 |
| 454 | GFM2 | 0.307631486 | 0.113363447 | 0.834811697 | 0.020643871 |
| 455 | GGCT | 0.385483042 | 0.15465726 | 0.960816041 | 0.040781281 |
| 456 | GGH | 0.594562671 | 0.388113706 | 0.910827844 | 0.016887021 |
| 457 | GHR | 1.358319769 | 1.010369621 | 1.826096666 | 0.042530811 |
| 458 | GIF | 0.795413919 | 0.642354656 | 0.984943903 | 0.035809169 |
| 459 | GKAP1 | 0.278885106 | 0.108358361 | 0.71777481 | 0.008110038 |
| 460 | GLO1 | 0.454614997 | 0.212931169 | 0.970617861 | 0.041647336 |
| 461 | GLRX3 | 0.325320396 | 0.116371989 | 0.909440161 | 0.032278487 |
| 462 | GNA15 | 0.581498909 | 0.344122897 | 0.982616922 | 0.042818211 |
| 463 | GNAI2 | 1.790108731 | 1.046041811 | 3.063442814 | 0.033655511 |
| 464 | GNB1 | 3.257310186 | 1.294857839 | 8.194003489 | 0.012108452 |
| 465 | GNPAT | 0.22155965 | 0.07054893 | 0.695810392 | 0.00984818 |
| 466 | GNPDA1 | 0.535591147 | 0.288441626 | 0.994509287 | 0.047996346 |
| 467 | GOLGA5 | 0.401215701 | 0.162080748 | 0.993171868 | 0.048293184 |
| 468 | GOLGA7 | 0.284175159 | 0.098981087 | 0.815868196 | 0.019379514 |
| 469 | GPN1 | 0.237423257 | 0.070731613 | 0.796953447 | 0.019949072 |
| 470 | GPN3 | 0.252644167 | 0.093358558 | 0.683698167 | 0.006757567 |
| 471 | GPR161 | 3.236703225 | 1.255632081 | 8.343405621 | 0.015051387 |
| 472 | GPR20 | 1.766695103 | 1.009035029 | 3.093263858 | 0.046432813 |
| 473 | GPR52 | 0.633981682 | 0.412917292 | 0.973397774 | 0.037231888 |
| 474 | GPSM2 | 0.48981736 | 0.265998527 | 0.901963813 | 0.021952098 |
| 475 | GPX2 | 0.410499021 | 0.243007463 | 0.693433215 | 0.000872878 |
| 476 | GPX3 | 1.745805909 | 1.037830473 | 2.936740008 | 0.035737672 |
| 477 | GRAMD1B | 1.518695809 | 1.133232621 | 2.035272297 | 0.005153831 |
| 478 | GRK7 | 0.669925362 | 0.472097142 | 0.950651786 | 0.024872617 |
| 479 | GSTM2 | 2.974541068 | 1.403805037 | 6.302794432 | 0.00443712 |
| 480 | GTDC1 | 0.218205842 | 0.087863906 | 0.541903856 | 0.001037933 |
| 481 | GTF2F1 | 4.850533634 | 1.754278231 | 13.41159921 | 0.002341321 |
| 482 | GTPBP1 | 3.454450807 | 1.181746776 | 10.0979589 | 0.023507037 |
| 483 | GTPBP4 | 0.144387041 | 0.044013185 | 0.473667556 | 0.001409157 |
| 484 | GUCY2E | 0.588916065 | 0.357669227 | 0.969672829 | 0.037433448 |
| 485 | GYS1 | 3.834745606 | 1.211253825 | 12.14053864 | 0.022259351 |
| 486 | GZMB | 0.739005056 | 0.548213685 | 0.99619635 | 0.047147271 |
| 487 | H2AFV | 0.223971968 | 0.063213444 | 0.793556547 | 0.020437017 |
| 488 | HAR1B | 0.586753957 | 0.411945644 | 0.83574183 | 0.003134469 |
| 489 | HAT1 | 0.233782719 | 0.078392257 | 0.697190788 | 0.009134995 |
| 490 | HCG4 | 1.695225389 | 1.112086467 | 2.584141796 | 0.014132548 |
| 491 | HCN3 | 2.327233975 | 1.065861973 | 5.081350225 | 0.034001462 |
| 492 | HERPUD1 | 2.273909208 | 1.265665533 | 4.085331356 | 0.00599439 |
| 493 | HES4 | 1.799753646 | 1.081712091 | 2.994431896 | 0.023675869 |
| 494 | HIPK3 | 4.422868845 | 1.2708657 | 15.39247525 | 0.019455778 |
| 495 | HMGB3 | 0.543084831 | 0.296075766 | 0.996167765 | 0.048567504 |
| 496 | HMGN5 | 0.651409625 | 0.447117342 | 0.949045047 | 0.025591746 |
| 497 | HMMR | 0.386253572 | 0.167467188 | 0.890871961 | 0.025683016 |
| 498 | HMOX1 | 1.408839895 | 1.044565853 | 1.900148128 | 0.024728633 |
| 499 | HNRNPH2 | 0.327807738 | 0.121995999 | 0.880831456 | 0.026996346 |
| 500 | HNRNPUL2 | 3.535780846 | 1.286252694 | 9.719510213 | 0.014369689 |
| 501 | HNRPDL | 0.310782284 | 0.112639159 | 0.857478238 | 0.024014217 |
| 502 | HOOK2 | 2.651628944 | 1.073628802 | 6.548945076 | 0.034517559 |
| 503 | HOXC4 | 1.411488878 | 1.018570231 | 1.955977893 | 0.038405168 |
| 504 | HPCAL4 | 1.468485824 | 1.106743802 | 1.948464144 | 0.00774812 |
| 505 | HPS3 | 0.467012552 | 0.232653221 | 0.937449839 | 0.032221895 |
| 506 | HRAS | 2.938210125 | 1.118053475 | 7.721525788 | 0.02879197 |
| 507 | HRSP12 | 0.478913341 | 0.276525544 | 0.829427853 | 0.008604752 |
| 508 | HS3ST5 | 1.38067353 | 1.081571026 | 1.762491182 | 0.00961333 |
| 509 | HSD11B1L | 2.048104488 | 1.181395514 | 3.550658476 | 0.010656588 |
| 510 | HSDL2 | 0.372824505 | 0.165864001 | 0.83802459 | 0.016959834 |
| 511 | HSF2 | 0.243455457 | 0.064571712 | 0.917902867 | 0.036935773 |
| 512 | HSF2BP | 0.65933292 | 0.475780925 | 0.91369762 | 0.012344365 |
| 513 | HSPA14 | 0.192892633 | 0.060883307 | 0.611129213 | 0.005158931 |
| 514 | HSPA1A | 1.483754846 | 1.039887535 | 2.117083213 | 0.029583389 |
| 515 | HTN3 | 0.57195222 | 0.365972052 | 0.893864272 | 0.014187268 |
| 516 | HYI | 2.993304027 | 1.431690706 | 6.258243458 | 0.0035726 |
| 517 | IARS | 0.24498661 | 0.084148947 | 0.713240526 | 0.009886362 |
| 518 | IARS2 | 0.239448441 | 0.07584362 | 0.75597072 | 0.014814179 |
| 519 | ICK | 0.466712649 | 0.223535738 | 0.974433432 | 0.042466467 |
| 520 | IER5L | 0.375616401 | 0.161940758 | 0.871230208 | 0.022542863 |
| 521 | IFI16 | 0.567921469 | 0.332471972 | 0.970111234 | 0.038354876 |
| 522 | IFNG | 0.697096059 | 0.513293451 | 0.946715596 | 0.020855 |
| 523 | IFNK | 0.669013322 | 0.452728464 | 0.988625324 | 0.043655844 |
| 524 | IFT74 | 0.383625817 | 0.241370827 | 0.609720609 | 5.06E-05 |
| 525 | IGFL4 | 0.782133367 | 0.640603538 | 0.954931667 | 0.015832229 |
| 526 | IKBKAP | 0.418729579 | 0.225966196 | 0.775932257 | 0.005674058 |
| 527 | IL17RC | 2.239228028 | 1.038252357 | 4.829406 | 0.039812336 |
| 528 | IL20RA | 0.632021176 | 0.400661615 | 0.996977879 | 0.04849798 |
| 529 | ILK | 4.841745863 | 1.785147243 | 13.13197166 | 0.001946354 |
| 530 | IMMP2L | 0.468946041 | 0.270439596 | 0.813158992 | 0.007008824 |
| 531 | INSM1 | 1.282542516 | 1.075065807 | 1.530060109 | 0.00571138 |
| 532 | INTS10 | 0.375844522 | 0.177623955 | 0.795270576 | 0.010497657 |
| 533 | INTS7 | 0.426200183 | 0.219560376 | 0.82731957 | 0.011731597 |
| 534 | INTS8 | 0.435673759 | 0.220396568 | 0.861227678 | 0.016864742 |
| 535 | IPO11 | 0.332736505 | 0.125322772 | 0.88342749 | 0.027191989 |
| 536 | IQCB1 | 0.430661092 | 0.202766119 | 0.914694116 | 0.028381813 |
| 537 | IQCG | 0.49027522 | 0.260450848 | 0.922898865 | 0.027204287 |
| 538 | IQCH | 0.642489463 | 0.453216476 | 0.910806937 | 0.012967561 |
| 539 | IQCK | 0.220604228 | 0.087202997 | 0.558079732 | 0.001414684 |
| 540 | IRF7 | 1.716323988 | 1.086379955 | 2.711544904 | 0.020611129 |
| 541 | IRGC | 0.630765983 | 0.423974912 | 0.938418087 | 0.022993154 |
| 542 | ISCA1 | 0.1621323 | 0.055670176 | 0.472189683 | 0.000850594 |
| 543 | ITFG2 | 2.448808607 | 1.085812328 | 5.522744068 | 0.030898848 |
| 544 | ITGA5 | 1.677642331 | 1.025815879 | 2.743653952 | 0.039253345 |
| 545 | ITGA7 | 2.028725021 | 1.117080332 | 3.684359209 | 0.02014456 |
| 546 | ITGB3 | 2.285517644 | 1.36408958 | 3.829360607 | 0.001694839 |
| 547 | ITGB3BP | 0.263626659 | 0.101436062 | 0.685150963 | 0.006221125 |
| 548 | JUP | 2.941574772 | 1.087031983 | 7.960080545 | 0.033647963 |
| 549 | KARS | 0.337580054 | 0.126614242 | 0.900059037 | 0.029975896 |
| 550 | KATNA1 | 0.243713539 | 0.063779927 | 0.931269318 | 0.039010878 |
| 551 | KCNC4 | 3.560096339 | 1.140136673 | 11.11646196 | 0.02883668 |
| 552 | KCNJ12 | 1.193802863 | 1.014062213 | 1.40540221 | 0.03336281 |
| 553 | KCNK5 | 2.14987234 | 1.078650678 | 4.284937812 | 0.029621339 |
| 554 | KCNU1 | 0.737665626 | 0.551981824 | 0.985812491 | 0.039730007 |
| 555 | KCTD12 | 1.988759863 | 1.135239868 | 3.483991276 | 0.016244238 |
| 556 | KDELR1 | 2.627369804 | 1.106730534 | 6.237355778 | 0.028534522 |
| 557 | KDM4B | 9.187787892 | 2.536820157 | 33.27608625 | 0.000730994 |
| 558 | KDM5C | 3.098419937 | 1.315350752 | 7.298590199 | 0.009681716 |
| 559 | KHDRBS3 | 0.619413281 | 0.392025098 | 0.978694514 | 0.040146796 |
| 560 | KIAA0195 | 2.620313215 | 1.045117276 | 6.569637214 | 0.039969704 |
| 561 | KIAA0247 | 2.622206607 | 1.12528896 | 6.110401627 | 0.025519972 |
| 562 | KIAA0368 | 0.452660658 | 0.216165207 | 0.947893852 | 0.035564622 |
| 563 | KIAA0586 | 0.400538784 | 0.173570828 | 0.924298856 | 0.031995295 |
| 564 | KIAA1009 | 0.506004178 | 0.267282567 | 0.957938377 | 0.036445137 |
| 565 | KIAA1107 | 0.699323836 | 0.509231643 | 0.960375958 | 0.027120904 |
| 566 | KIAA1430 | 0.444062286 | 0.197474866 | 0.998564111 | 0.04959514 |
| 567 | KIF11 | 0.429623906 | 0.220685547 | 0.836378743 | 0.012931666 |
| 568 | KIF14 | 0.525935225 | 0.305171494 | 0.906401371 | 0.020676958 |
| 569 | KIF18A | 0.545587763 | 0.311704361 | 0.954962598 | 0.033895971 |
| 570 | KIF20B | 0.447119641 | 0.239483825 | 0.834778607 | 0.011508141 |
| 571 | KIF24 | 0.521809043 | 0.302532669 | 0.900017439 | 0.019349908 |
| 572 | KIF27 | 0.631774585 | 0.428128617 | 0.932287893 | 0.020715481 |
| 573 | KIN | 0.169282807 | 0.066913586 | 0.428263832 | 0.00017636 |
| 574 | KIR2DL5A | 0.5791511 | 0.388405794 | 0.863571044 | 0.00737201 |
| 575 | KIRREL | 1.776196853 | 1.051216804 | 3.001165171 | 0.031824911 |
| 576 | KLHDC8B | 2.162854995 | 1.010641616 | 4.628685043 | 0.046896651 |
| 577 | KPNA1 | 0.285388526 | 0.108914785 | 0.747801237 | 0.010732977 |
| 578 | KPNA3 | 0.306131839 | 0.111019103 | 0.844149347 | 0.022175605 |
| 579 | KPRP | 0.739167756 | 0.569453497 | 0.959461967 | 0.023152698 |
| 580 | KRIT1 | 0.652555322 | 0.447584892 | 0.951391468 | 0.026486711 |
| 581 | KRR1 | 0.46418974 | 0.301039891 | 0.715759342 | 0.000513733 |
| 582 | KRT6B | 0.840781075 | 0.716442342 | 0.986698822 | 0.033673541 |
| 583 | KRT6C | 0.813937081 | 0.683525045 | 0.969230868 | 0.02084683 |
| 584 | KRT72 | 0.770059643 | 0.594840672 | 0.99689191 | 0.047300874 |
| 585 | KRTAP4-1 | 0.707963311 | 0.528118018 | 0.949053115 | 0.020906751 |
| 586 | KTN1 | 0.553964568 | 0.310678302 | 0.987763679 | 0.045318316 |
| 587 | LAMB2 | 2.255744862 | 1.209427379 | 4.20726781 | 0.010532196 |
| 588 | LAMTOR3 | 0.531275352 | 0.283939006 | 0.994063844 | 0.047862235 |
| 589 | LAP3 | 0.402006045 | 0.185450967 | 0.871437138 | 0.02096707 |
| 590 | LARP7 | 0.483135033 | 0.245772459 | 0.949738066 | 0.034900989 |
| 591 | LASP1 | 2.627631486 | 1.327372714 | 5.201588935 | 0.005557766 |
| 592 | LBR | 0.308755421 | 0.115949611 | 0.822166712 | 0.018681643 |
| 593 | LCE3A | 0.59726594 | 0.383299584 | 0.930673077 | 0.022759578 |
| 594 | LDLR | 1.964397866 | 1.10748388 | 3.484347758 | 0.020937462 |
| 595 | LENG1 | 4.884138306 | 1.560421714 | 15.28741031 | 0.006444436 |
| 596 | LEPR | 0.494389796 | 0.279034862 | 0.875952447 | 0.015787437 |
| 597 | LIG4 | 0.716761848 | 0.52604802 | 0.976617203 | 0.034869512 |
| 598 | LILRP2 | 0.727735584 | 0.535478078 | 0.989021029 | 0.042306238 |
| 599 | LINC00087 | 2.18516492 | 1.029767511 | 4.636916273 | 0.041712276 |
| 600 | LINC00158 | 0.732725135 | 0.537945524 | 0.998030655 | 0.048556301 |
| 601 | LINC00161 | 0.687104726 | 0.494782314 | 0.954183064 | 0.025097508 |
| 602 | LINC00230A | 0.574075759 | 0.405479876 | 0.81277271 | 0.001756675 |
| 603 | LINC00266-1 | 0.372879168 | 0.169208912 | 0.821699471 | 0.014400948 |
| 604 | LINC00294 | 3.613787214 | 1.26485519 | 10.32486416 | 0.01645664 |
| 605 | LINC00319 | 0.673034563 | 0.46254559 | 0.979310001 | 0.038525347 |
| 606 | LINC00483 | 1.797131599 | 1.048117836 | 3.081411147 | 0.033106167 |
| 607 | LIX1L | 2.102573005 | 1.071305244 | 4.12656735 | 0.030759259 |
| 608 | LLPH | 0.361412403 | 0.135791541 | 0.961907671 | 0.041577587 |
| 609 | LMBR1 | 0.271631866 | 0.096562432 | 0.76410535 | 0.013517926 |
| 610 | LMO4 | 2.417521233 | 1.048644869 | 5.573296626 | 0.038319247 |
| 611 | LMOD1 | 1.905668987 | 1.163987538 | 3.119942584 | 0.01035672 |
| 612 | LOC100127991 | 0.637452878 | 0.440289483 | 0.92290683 | 0.017084352 |
| 613 | LOC100128657 | 0.745166811 | 0.59974548 | 0.925848706 | 0.007918925 |
| 614 | LOC100129171 | 0.679273713 | 0.470437908 | 0.980815468 | 0.03908289 |
| 615 | LOC100129449 | 0.663588358 | 0.458875615 | 0.959627172 | 0.029337239 |
| 616 | LOC100129894 | 1.479074918 | 1.028567405 | 2.126902528 | 0.034691367 |
| 617 | LOC100129924 | 0.748082773 | 0.568401658 | 0.984564045 | 0.038362238 |
| 618 | LOC100130691 | 0.494562274 | 0.272936562 | 0.896149058 | 0.020260058 |
| 619 | LOC100130856 | 3.17618137 | 1.133519961 | 8.899823949 | 0.027923033 |
| 620 | LOC100131691 | 0.748136659 | 0.563295994 | 0.993631175 | 0.045060237 |
| 621 | LOC100132356 | 3.272433213 | 1.18662588 | 9.024595966 | 0.021988083 |
| 622 | LOC100132724 | 0.408925361 | 0.183671485 | 0.910429569 | 0.028542054 |
| 623 | LOC100132987 | 0.595247074 | 0.366167603 | 0.967641804 | 0.036380505 |
| 624 | LOC100134368 | 0.70995004 | 0.539866769 | 0.933617495 | 0.014224942 |
| 625 | LOC100270746 | 1.575685002 | 1.009836678 | 2.45859878 | 0.045167991 |
| 626 | LOC100505890 | 0.751095309 | 0.581519008 | 0.970121621 | 0.028357566 |
| 627 | LOC100506128 | 0.763270982 | 0.597295735 | 0.975367072 | 0.030824721 |
| 628 | LOC100507410 | 0.724743134 | 0.550795151 | 0.953626061 | 0.021501557 |
| 629 | LOC100653245 | 1.482337842 | 1.112694955 | 1.974777964 | 0.007153086 |
| 630 | LOC158376 | 1.984739893 | 1.001492276 | 3.933322841 | 0.049502631 |
| 631 | LOC202781 | 0.430188287 | 0.211175608 | 0.87634156 | 0.020148829 |
| 632 | LOC254128 | 0.230619671 | 0.071717648 | 0.741594774 | 0.013831609 |
| 633 | LOC254896 | 0.472269323 | 0.272910389 | 0.817258422 | 0.007336246 |
| 634 | LOC257396 | 0.500722104 | 0.286802347 | 0.874200045 | 0.014981255 |
| 635 | LOC283352 | 1.560817325 | 1.121110623 | 2.172979786 | 0.00836142 |
| 636 | LOC285419 | 0.721535554 | 0.526328795 | 0.989141313 | 0.042580735 |
| 637 | LOC285740 | 0.551498061 | 0.309685703 | 0.982125132 | 0.043256755 |
| 638 | LOC285943 | 0.701387072 | 0.520669499 | 0.944829349 | 0.019633033 |
| 639 | LOC286299 | 0.767640852 | 0.590548906 | 0.997838573 | 0.048139601 |
| 640 | LOC339290 | 0.619149028 | 0.389442976 | 0.984343132 | 0.042695543 |
| 641 | LOC339505 | 0.696721354 | 0.516134874 | 0.940491855 | 0.018237121 |
| 642 | LOC339975 | 0.753847072 | 0.600586361 | 0.946217639 | 0.014822327 |
| 643 | LOC340340 | 0.584876322 | 0.420284355 | 0.813925875 | 0.001467451 |
| 644 | LOC388906 | 0.500574255 | 0.268078321 | 0.934706633 | 0.029864092 |
| 645 | LOC399815 | 0.625623218 | 0.426184531 | 0.918391877 | 0.016637607 |
| 646 | LOC399875 | 0.679945352 | 0.494245429 | 0.935417213 | 0.017779192 |
| 647 | LOC439949 | 0.649059335 | 0.444181807 | 0.94843601 | 0.02551376 |
| 648 | LOC441461 | 0.560528072 | 0.339114298 | 0.926506847 | 0.023966021 |
| 649 | LOC643339 | 0.522075884 | 0.304321101 | 0.895643542 | 0.018265644 |
| 650 | LOC644919 | 1.211013577 | 1.006632705 | 1.456890757 | 0.042350489 |
| 651 | LOC646241 | 0.801319674 | 0.653858399 | 0.982037122 | 0.032789252 |
| 652 | LOC728739 | 0.423868849 | 0.180413353 | 0.995850906 | 0.048895222 |
| 653 | LOC728743 | 0.416187765 | 0.204837023 | 0.845610101 | 0.015367631 |
| 654 | LOC729799 | 3.402048334 | 1.034005802 | 11.19329586 | 0.043904868 |
| 655 | LOC91450 | 2.091796219 | 1.027865631 | 4.25698777 | 0.041772689 |
| 656 | LONRF1 | 0.557393721 | 0.328201154 | 0.946638231 | 0.030549313 |
| 657 | LRP8 | 0.608368311 | 0.394603493 | 0.937933924 | 0.024444561 |
| 658 | LRPPRC | 0.374305029 | 0.154759053 | 0.905305713 | 0.029203277 |
| 659 | LRRC27 | 0.268794101 | 0.078493404 | 0.92046293 | 0.03644493 |
| 660 | LRRC34 | 0.669612455 | 0.496388016 | 0.903286997 | 0.008640702 |
| 661 | LRRC43 | 0.72888493 | 0.561933461 | 0.945437988 | 0.017186137 |
| 662 | LRRC6 | 0.679063824 | 0.471778046 | 0.977425044 | 0.037265577 |
| 663 | LRRC69 | 0.543135736 | 0.330214147 | 0.893348847 | 0.016209571 |
| 664 | LRRCC1 | 0.596601022 | 0.41604636 | 0.855512303 | 0.004977057 |
| 665 | LRRIQ4 | 0.775349358 | 0.625193676 | 0.961568632 | 0.020514644 |
| 666 | LRRN2 | 2.612159884 | 1.164573654 | 5.859122125 | 0.019826635 |
| 667 | LSM3 | 0.375189268 | 0.149694845 | 0.940359618 | 0.036515985 |
| 668 | LSM5 | 0.439728434 | 0.198641052 | 0.973419608 | 0.042722832 |
| 669 | LSR | 4.285276999 | 1.686571928 | 10.88812085 | 0.002223696 |
| 670 | LTV1 | 0.303942633 | 0.129024728 | 0.715995497 | 0.006446579 |
| 671 | LY6G6F | 0.715543881 | 0.547364981 | 0.935396059 | 0.014344554 |
| 672 | LYAR | 0.392721401 | 0.202741592 | 0.760722541 | 0.005593829 |
| 673 | LYPLA1 | 0.403635193 | 0.203492571 | 0.800625635 | 0.009423132 |
| 674 | LYRM5 | 0.359542835 | 0.175074857 | 0.738375871 | 0.005335606 |
| 675 | MAD2L1 | 0.535223275 | 0.286611412 | 0.999485511 | 0.049811525 |
| 676 | MADD | 5.130663923 | 1.514921547 | 17.37628746 | 0.008605844 |
| 677 | MAFF | 2.424402642 | 1.234344358 | 4.761822039 | 0.010132998 |
| 678 | MAFK | 2.385383309 | 1.057280857 | 5.381780529 | 0.036247821 |
| 679 | MAGEA11 | 0.748485627 | 0.58942032 | 0.950477468 | 0.01747131 |
| 680 | MAK16 | 0.399673256 | 0.215604518 | 0.74088759 | 0.003587362 |
| 681 | MALL | 1.557698839 | 1.065318912 | 2.277651928 | 0.022232322 |
| 682 | MAP1B | 1.675919452 | 1.085690147 | 2.587023579 | 0.019746522 |
| 683 | MAP3K11 | 2.624786068 | 1.239076815 | 5.560189506 | 0.011745729 |
| 684 | MAPK7 | 4.067201426 | 1.509909193 | 10.95571012 | 0.00552054 |
| 685 | MAPKAP1 | 0.088167966 | 0.026452381 | 0.293871096 | 7.70E-05 |
| 686 | MAPKAPK2 | 3.24190221 | 1.411003437 | 7.448550201 | 0.005585348 |
| 687 | MAPKBP1 | 2.516541676 | 1.041939254 | 6.078072195 | 0.040239707 |
| 688 | 5-Mar | 0.355983975 | 0.189153495 | 0.669956377 | 0.001367196 |
| 689 | MARCO | 1.338606288 | 1.014568884 | 1.766136161 | 0.039184504 |
| 690 | MARK2 | 8.225015369 | 2.15104036 | 31.45030614 | 0.002075125 |
| 691 | MAST2 | 1.855927087 | 1.055377459 | 3.263728366 | 0.031784835 |
| 692 | MATR3 | 0.275994586 | 0.082208709 | 0.926580809 | 0.037217839 |
| 693 | MBD1 | 14.9538961 | 2.709631517 | 82.52746071 | 0.001911043 |
| 694 | MBD4 | 0.271741342 | 0.106748796 | 0.691748851 | 0.006275898 |
| 695 | MBLAC2 | 0.372123622 | 0.165945388 | 0.834467238 | 0.016432831 |
| 696 | MCAM | 1.760610843 | 1.130269177 | 2.742488784 | 0.01236704 |
| 697 | MCOLN2 | 1.565914295 | 1.025972923 | 2.390011983 | 0.037633806 |
| 698 | MCOLN3 | 1.419322322 | 1.016472247 | 1.98183065 | 0.039793724 |
| 699 | MDK | 1.406564413 | 1.017707291 | 1.944000466 | 0.038802535 |
| 700 | ME2 | 0.465032271 | 0.230885967 | 0.936631254 | 0.032095721 |
| 701 | MED25 | 3.69445939 | 1.095647574 | 12.45750049 | 0.035095233 |
| 702 | MEF2D | 4.13597148 | 1.041711745 | 16.42129905 | 0.043585892 |
| 703 | MEIG1 | 0.634283752 | 0.405936701 | 0.991080324 | 0.045574681 |
| 704 | MEMO1 | 0.318227057 | 0.116224361 | 0.871318706 | 0.025880333 |
| 705 | MET | 0.407022802 | 0.20176602 | 0.821087522 | 0.012055322 |
| 706 | METAP2 | 0.298159527 | 0.141239028 | 0.629423075 | 0.001501658 |
| 707 | METTL5 | 0.206073458 | 0.057414268 | 0.739646632 | 0.015414189 |
| 708 | METTL9 | 0.447421767 | 0.2112011 | 0.947846569 | 0.035745593 |
| 709 | MFHAS1 | 0.490639995 | 0.277733667 | 0.866757019 | 0.014187325 |
| 710 | MGC16075 | 0.67595466 | 0.522859023 | 0.873877436 | 0.002800283 |
| 711 | MIB1 | 0.510125994 | 0.282503197 | 0.921152512 | 0.025591863 |
| 712 | MINA | 0.403002254 | 0.171045696 | 0.949517118 | 0.037669473 |
| 713 | MIOS | 0.303082126 | 0.110451412 | 0.831666826 | 0.0204571 |
| 714 | MITD1 | 0.16176765 | 0.049190095 | 0.531992718 | 0.00270835 |
| 715 | MKI67IP | 0.177128196 | 0.066778603 | 0.469827106 | 0.000505735 |
| 716 | MKS1 | 0.24230921 | 0.084044447 | 0.6986036 | 0.008693831 |
| 717 | MLXIP | 3.076668787 | 1.003139697 | 9.436263816 | 0.04936261 |
| 718 | MMP9 | 1.546689371 | 1.060251816 | 2.256301734 | 0.023596376 |
| 719 | MNAT1 | 0.337884441 | 0.130792779 | 0.872876137 | 0.025043042 |
| 720 | MND1 | 0.537543859 | 0.302049091 | 0.956643836 | 0.034800148 |
| 721 | MOB2 | 2.808409889 | 1.247787798 | 6.320919406 | 0.012602907 |
| 722 | MOB3A | 3.052866144 | 1.123808066 | 8.293223705 | 0.028605286 |
| 723 | MORN2 | 0.197780026 | 0.070430409 | 0.555398426 | 0.002096248 |
| 724 | MPP7 | 0.512932674 | 0.28996976 | 0.907335744 | 0.021783571 |
| 725 | MRGPRX3 | 0.782550267 | 0.623712693 | 0.981838155 | 0.03414879 |
| 726 | MRPL19 | 0.291303834 | 0.094279811 | 0.900064632 | 0.032121699 |
| 727 | MRPL3 | 0.364459312 | 0.138441903 | 0.959468103 | 0.040978862 |
| 728 | MRPL32 | 0.224883115 | 0.068766312 | 0.735424283 | 0.0135756 |
| 729 | MRPL42 | 0.335679701 | 0.121174598 | 0.929904973 | 0.035750427 |
| 730 | MRPL50 | 0.244202448 | 0.091961665 | 0.64847494 | 0.004666383 |
| 731 | MRPS18C | 0.253425477 | 0.093772022 | 0.684900156 | 0.006807841 |
| 732 | MRPS2 | 0.365852913 | 0.137161564 | 0.97584447 | 0.044556886 |
| 733 | MRPS23 | 0.328774794 | 0.126963666 | 0.851368492 | 0.021938701 |
| 734 | MRPS9 | 0.35620629 | 0.13416 | 0.945758208 | 0.038274673 |
| 735 | MRRF | 0.360672601 | 0.161924474 | 0.803366669 | 0.012567202 |
| 736 | MS4A6E | 0.695306221 | 0.511810588 | 0.944589176 | 0.020092582 |
| 737 | MSL3 | 0.297985073 | 0.127931862 | 0.694081228 | 0.005009616 |
| 738 | MSL3P1 | 0.287221346 | 0.121474051 | 0.679125298 | 0.00449347 |
| 739 | MST1 | 1.66873245 | 1.119033463 | 2.488458192 | 0.012018966 |
| 740 | MT1B | 1.470295404 | 1.008095423 | 2.144408678 | 0.045302276 |
| 741 | MT1L | 1.524624468 | 1.052788065 | 2.207927546 | 0.025599307 |
| 742 | MT2A | 1.586108532 | 1.045278523 | 2.406765489 | 0.030150883 |
| 743 | MTERFD1 | 0.338039575 | 0.137490408 | 0.831118007 | 0.018128419 |
| 744 | MTERFD3 | 0.377643069 | 0.179839791 | 0.793007417 | 0.010091584 |
| 745 | MTFR1 | 0.489265848 | 0.251632592 | 0.951311865 | 0.035109914 |
| 746 | MTHFD2L | 0.466129428 | 0.277007728 | 0.784370331 | 0.004044625 |
| 747 | MTRF1L | 0.342601528 | 0.119574202 | 0.981614806 | 0.04609578 |
| 748 | MTSS1 | 1.738116854 | 1.119908113 | 2.697587564 | 0.013704116 |
| 749 | MUS81 | 5.52653751 | 1.328823463 | 22.98470617 | 0.01872745 |
| 750 | MUTYH | 2.841265504 | 1.102558378 | 7.321870499 | 0.030609454 |
| 751 | MVP | 2.925440337 | 1.238100267 | 6.912365171 | 0.014413481 |
| 752 | MYC | 0.529268154 | 0.330007353 | 0.848844053 | 0.008292525 |
| 753 | MYEOV2 | 0.588459512 | 0.358711716 | 0.965356249 | 0.035766487 |
| 754 | MYH16 | 0.694634487 | 0.502223358 | 0.960761907 | 0.027675642 |
| 755 | MYH9 | 6.636071927 | 2.210353967 | 19.92325722 | 0.000740831 |
| 756 | MYO1D | 2.600767933 | 1.118506231 | 6.047345695 | 0.026412119 |
| 757 | MYO1E | 2.473394109 | 1.098595101 | 5.56863799 | 0.028738338 |
| 758 | MYO9B | 2.811817554 | 1.056312973 | 7.4848252 | 0.038486284 |
| 759 | MZT1 | 0.493873591 | 0.254630845 | 0.957900933 | 0.036868631 |
| 760 | NAA20 | 0.395862314 | 0.189942108 | 0.825024915 | 0.013386357 |
| 761 | NAA25 | 0.339343802 | 0.143109563 | 0.804657732 | 0.01415398 |
| 762 | NAA38 | 0.437726108 | 0.194928955 | 0.982943479 | 0.045322879 |
| 763 | NAA40 | 4.055992093 | 1.310288253 | 12.55530744 | 0.015152056 |
| 764 | NAE1 | 0.183896951 | 0.070539102 | 0.479423295 | 0.000532765 |
| 765 | NBN | 0.481875178 | 0.27791021 | 0.835534927 | 0.009327069 |
| 766 | NCAPG2 | 0.356870785 | 0.189751752 | 0.671175659 | 0.001387866 |
| 767 | NCBP1 | 0.465919384 | 0.249363282 | 0.870540646 | 0.016635656 |
| 768 | NCKIPSD | 3.816596011 | 1.024011643 | 14.22484325 | 0.046008789 |
| 769 | NDC80 | 0.514128034 | 0.280712183 | 0.941632218 | 0.03118141 |
| 770 | NDOR1 | 0.364481025 | 0.147147802 | 0.902809387 | 0.02919105 |
| 771 | NDRG1 | 2.45621322 | 1.476371544 | 4.086358483 | 0.00054014 |
| 772 | NDST1 | 2.675347156 | 1.107690804 | 6.461624834 | 0.028721051 |
| 773 | NEBL | 0.484266163 | 0.33437634 | 0.701346622 | 0.000124396 |
| 774 | NEDD1 | 0.319300714 | 0.119416936 | 0.853756165 | 0.022903038 |
| 775 | NEIL1 | 2.870787146 | 1.25182764 | 6.583509242 | 0.012761465 |
| 776 | NEIL2 | 0.489507485 | 0.243147826 | 0.985481061 | 0.045399928 |
| 777 | NEIL3 | 0.574155563 | 0.346202845 | 0.952200754 | 0.0315766 |
| 778 | NEK11 | 0.479239224 | 0.256627528 | 0.894955565 | 0.020986239 |
| 779 | NEK2 | 0.337582767 | 0.17113774 | 0.665908785 | 0.001730001 |
| 780 | NEUROD6 | 0.623509317 | 0.405874715 | 0.957842046 | 0.031036713 |
| 781 | NFE2L1 | 3.039533762 | 1.028731501 | 8.980735481 | 0.044303269 |
| 782 | NFE2L3 | 0.57255335 | 0.347670728 | 0.942895999 | 0.02845293 |
| 783 | NINJ2 | 0.568261609 | 0.325317593 | 0.992633855 | 0.047039605 |
| 784 | NISCH | 3.169356769 | 1.072001323 | 9.370158517 | 0.037008065 |
| 785 | NKX2-1 | 1.165259612 | 1.009676488 | 1.34481686 | 0.036468616 |
| 786 | NME7 | 0.273830879 | 0.114487538 | 0.654947703 | 0.003601278 |
| 787 | NMI | 0.240953955 | 0.087442864 | 0.663962799 | 0.005926132 |
| 788 | NOC3L | 0.299416791 | 0.112793363 | 0.794819951 | 0.015478502 |
| 789 | NOL10 | 0.229740255 | 0.081258932 | 0.649535788 | 0.005542335 |
| 790 | NOL11 | 0.206977737 | 0.068858662 | 0.622140804 | 0.005029272 |
| 791 | NOL8 | 0.437311128 | 0.197986742 | 0.965928429 | 0.04078563 |
| 792 | NOM1 | 0.544122227 | 0.321746869 | 0.920192318 | 0.023193771 |
| 793 | NPHP1 | 0.506000567 | 0.295716011 | 0.865819113 | 0.012929876 |
| 794 | NPTN | 4.048580493 | 1.47725273 | 11.09559906 | 0.006557782 |
| 795 | NQO1 | 0.657199529 | 0.439577833 | 0.982559147 | 0.040784831 |
| 796 | NRADDP | 0.590454111 | 0.374545232 | 0.930824979 | 0.023290878 |
| 797 | NRGN | 1.982383823 | 1.027724049 | 3.823833473 | 0.041196139 |
| 798 | NSL1 | 0.255372361 | 0.075332856 | 0.865691894 | 0.028414591 |
| 799 | NSMAF | 0.482811968 | 0.246410015 | 0.946014294 | 0.033864846 |
| 800 | NSUN6 | 0.205937977 | 0.071915619 | 0.589725168 | 0.003242307 |
| 801 | NUB1 | 0.227797339 | 0.062273193 | 0.833289977 | 0.025379687 |
| 802 | NUDCD1 | 0.561828214 | 0.34252271 | 0.921547483 | 0.022397779 |
| 803 | NUDT15 | 0.553117287 | 0.309424225 | 0.988735558 | 0.045696187 |
| 804 | NUDT5 | 0.201240157 | 0.059481575 | 0.680842775 | 0.00993328 |
| 805 | NUDT7 | 0.480064884 | 0.274553475 | 0.83940767 | 0.010052985 |
| 806 | NUF2 | 0.48978677 | 0.283985253 | 0.844730763 | 0.010266111 |
| 807 | NUMA1 | 2.768893443 | 1.107322211 | 6.923703709 | 0.029407887 |
| 808 | NUP133 | 0.259370698 | 0.075340869 | 0.892917216 | 0.032392613 |
| 809 | NUP35 | 0.331229565 | 0.125199912 | 0.876302728 | 0.026015943 |
| 810 | NUP37 | 0.353169022 | 0.13098992 | 0.952198143 | 0.039709475 |
| 811 | NUP50 | 0.202661869 | 0.058864442 | 0.697735874 | 0.011388299 |
| 812 | NUP54 | 0.305024111 | 0.115417383 | 0.806115211 | 0.016636944 |
| 813 | NXT2 | 0.291303635 | 0.095693257 | 0.886768941 | 0.029890364 |
| 814 | NYX | 1.474475539 | 1.000658907 | 2.172646543 | 0.049611978 |
| 815 | OAF | 2.312982644 | 1.250156352 | 4.279375696 | 0.007558177 |
| 816 | OCA2 | 0.724379778 | 0.586816486 | 0.89419107 | 0.002693123 |
| 817 | ODF2 | 0.347965445 | 0.15776793 | 0.767456041 | 0.008901875 |
| 818 | OPA1 | 0.183587388 | 0.049928438 | 0.675052741 | 0.010727046 |
| 819 | OPLAH | 1.649180065 | 1.028541494 | 2.644321988 | 0.037821133 |
| 820 | OR2B3 | 0.539429098 | 0.340595527 | 0.854338147 | 0.008513365 |
| 821 | OR4F4 | 0.771314062 | 0.605939802 | 0.981822583 | 0.034948073 |
| 822 | OR51E1 | 1.370361333 | 1.011812662 | 1.85596628 | 0.041766039 |
| 823 | OR51M1 | 0.738399948 | 0.553157885 | 0.985676058 | 0.039603267 |
| 824 | OR5AR1 | 1.831362486 | 1.129830197 | 2.968489039 | 0.014076393 |
| 825 | OR5M10 | 1.341788027 | 1.030416058 | 1.747250632 | 0.029081801 |
| 826 | OR7E91P | 0.535028073 | 0.295089745 | 0.97006095 | 0.039390444 |
| 827 | ORC5 | 0.266438191 | 0.096332348 | 0.73692078 | 0.010831307 |
| 828 | OSCP1 | 0.419728494 | 0.179026479 | 0.984055596 | 0.045832217 |
| 829 | OTC | 1.235898734 | 1.027496377 | 1.48657038 | 0.024585948 |
| 830 | OTUD6B | 0.469188319 | 0.222103251 | 0.991150187 | 0.047338347 |
| 831 | P4HA2 | 7.539900358 | 2.657416856 | 21.39299195 | 0.000146561 |
| 832 | PACRGL | 0.359519357 | 0.157645137 | 0.819905838 | 0.015014443 |
| 833 | PAF1 | 8.667019056 | 2.013440051 | 37.30789962 | 0.003735555 |
| 834 | PAG1 | 1.970264279 | 1.085337614 | 3.576713163 | 0.025804562 |
| 835 | PALB2 | 0.41479305 | 0.188774169 | 0.911423821 | 0.028460691 |
| 836 | PANK4 | 3.177690443 | 1.129802893 | 8.93759134 | 0.028431977 |
| 837 | PAQR9 | 0.585174517 | 0.356356696 | 0.960917021 | 0.034216764 |
| 838 | PARG | 0.298899701 | 0.104606089 | 0.854071035 | 0.024168844 |
| 839 | PAXIP1 | 0.319642804 | 0.112116418 | 0.911298487 | 0.03286473 |
| 840 | PBK | 0.503136791 | 0.306239239 | 0.826630287 | 0.006696388 |
| 841 | PBXIP1 | 3.225428125 | 1.074974228 | 9.677800939 | 0.036714431 |
| 842 | PCCA | 0.700750781 | 0.520526702 | 0.943374578 | 0.019065829 |
| 843 | PCDH12 | 1.534998413 | 1.01911994 | 2.312014548 | 0.040306619 |
| 844 | PCDHB2 | 1.404886733 | 1.064800775 | 1.853592502 | 0.016218589 |
| 845 | PCDHB5 | 1.496103182 | 1.04733917 | 2.137153651 | 0.026816855 |
| 846 | PCGF6 | 0.388606121 | 0.152148184 | 0.992550246 | 0.048200843 |
| 847 | PCM1 | 0.48267649 | 0.265839627 | 0.876380233 | 0.016685167 |
| 848 | PCYT1A | 3.231985952 | 1.083081702 | 9.644455423 | 0.035462076 |
| 849 | PDC | 0.680899528 | 0.482779 | 0.960323807 | 0.028471667 |
| 850 | PDCL | 0.173585964 | 0.051750958 | 0.582251766 | 0.004570065 |
| 851 | PDDC1 | 2.278897018 | 1.042416688 | 4.982049574 | 0.039011826 |
| 852 | PDE2A | 2.664635353 | 1.430017333 | 4.965171678 | 0.002026171 |
| 853 | PDE5A | 2.057511842 | 1.024502716 | 4.132107132 | 0.042559563 |
| 854 | PDE8B | 2.16921658 | 1.224367917 | 3.843207998 | 0.007962742 |
| 855 | PDGFA | 3.750399345 | 1.86407594 | 7.545559139 | 0.000210618 |
| 856 | PDIA5 | 9.619768523 | 2.393941587 | 38.65589157 | 0.001422367 |
| 857 | PEAK1 | 4.108552835 | 1.238529167 | 13.62923607 | 0.020909404 |
| 858 | PER1 | 3.146838511 | 1.428274401 | 6.933256388 | 0.004449183 |
| 859 | PFKFB4 | 1.927338002 | 1.131305028 | 3.283492676 | 0.015785895 |
| 860 | PGC | 0.540888496 | 0.30120711 | 0.971293027 | 0.039639965 |
| 861 | PHF1 | 5.234154935 | 1.671751155 | 16.38783248 | 0.004477331 |
| 862 | PHF19 | 0.320530813 | 0.125077655 | 0.821409722 | 0.017801981 |
| 863 | PHF21B | 2.120761857 | 1.294894267 | 3.473357607 | 0.002820528 |
| 864 | PHLDA1 | 0.577312194 | 0.366793459 | 0.908656797 | 0.017602809 |
| 865 | PHLDB1 | 2.305681839 | 1.059088841 | 5.019568271 | 0.035326437 |
| 866 | PHRF1 | 4.936382859 | 2.061379154 | 11.82115172 | 0.000338985 |
| 867 | PI4K2B | 0.487197438 | 0.251783386 | 0.942720435 | 0.03275258 |
| 868 | PICALM | 4.113763556 | 1.224636984 | 13.81883024 | 0.022152106 |
| 869 | PIDD | 2.636454477 | 1.154418463 | 6.021120097 | 0.021405063 |
| 870 | PIGX | 0.243314047 | 0.069409946 | 0.85292856 | 0.027206903 |
| 871 | PIK3CA | 0.466869742 | 0.231862965 | 0.940069734 | 0.032922428 |
| 872 | PION | 0.456500082 | 0.220103803 | 0.946791111 | 0.035129072 |
| 873 | PITRM1 | 0.307732483 | 0.120215772 | 0.787744235 | 0.013992585 |
| 874 | PITX2 | 0.792749491 | 0.636439783 | 0.987448886 | 0.038201643 |
| 875 | PIWIL3 | 0.783693914 | 0.620060949 | 0.990509322 | 0.041373413 |
| 876 | PKD1 | 5.492724446 | 1.718070292 | 17.56041181 | 0.004070542 |
| 877 | PKIA | 1.67882549 | 1.032989279 | 2.728445574 | 0.036532095 |
| 878 | PKM2 | 2.090208384 | 1.04594326 | 4.177063191 | 0.036876758 |
| 879 | PKP3 | 3.107199643 | 1.313734698 | 7.349040593 | 0.009844643 |
| 880 | PLAA | 0.354000734 | 0.178335576 | 0.702700619 | 0.002992025 |
| 881 | PLCB4 | 0.475654259 | 0.331278554 | 0.682950861 | 5.67E-05 |
| 882 | PLCG2 | 2.132042044 | 1.098848912 | 4.136695433 | 0.025175298 |
| 883 | PLD3 | 3.387169891 | 1.152934274 | 9.951061505 | 0.026501637 |
| 884 | PLEKHA2 | 0.480294425 | 0.236207118 | 0.976612125 | 0.042834312 |
| 885 | PLEKHA7 | 2.719760154 | 1.135587017 | 6.51389562 | 0.02474902 |
| 886 | PLEKHA8 | 0.379675932 | 0.17403191 | 0.828318288 | 0.014965352 |
| 887 | PLEKHG2 | 1.895814189 | 1.02154792 | 3.518299406 | 0.042607164 |
| 888 | PLEKHH3 | 2.199091854 | 1.032425808 | 4.684118649 | 0.041084068 |
| 889 | PLEKHO1 | 2.115559136 | 1.199635523 | 3.730791873 | 0.009630669 |
| 890 | PLK4 | 0.423766949 | 0.23877445 | 0.75208393 | 0.003353042 |
| 891 | PLOD1 | 3.222419108 | 1.458034544 | 7.121905959 | 0.00382895 |
| 892 | PLOD2 | 1.965751412 | 1.082124702 | 3.570918035 | 0.026479673 |
| 893 | PLSCR2 | 0.720529837 | 0.525673009 | 0.987616327 | 0.041607771 |
| 894 | PLVAP | 1.80293491 | 1.071782624 | 3.032867129 | 0.026336873 |
| 895 | PLXNA3 | 1.895517145 | 1.024753428 | 3.506194907 | 0.041561342 |
| 896 | PLXND1 | 1.758888061 | 1.028581337 | 3.00772248 | 0.039120806 |
| 897 | PMPCA | 0.278566177 | 0.084445476 | 0.918925655 | 0.035833988 |
| 898 | PNO1 | 0.429840285 | 0.204639461 | 0.902869219 | 0.025760565 |
| 899 | PNPLA2 | 3.132277288 | 1.428193864 | 6.869628316 | 0.004379587 |
| 900 | PNPT1 | 0.391869754 | 0.180496487 | 0.85077503 | 0.017857864 |
| 901 | POC1B | 0.285463064 | 0.134640806 | 0.605233756 | 0.001077082 |
| 902 | POC5 | 0.225327852 | 0.075469538 | 0.672756745 | 0.007580645 |
| 903 | POLA1 | 0.374167358 | 0.151268811 | 0.925512741 | 0.033379701 |
| 904 | POLB | 0.337463859 | 0.145546255 | 0.782444426 | 0.011349838 |
| 905 | POLR1B | 0.377940387 | 0.166041689 | 0.860259473 | 0.020414167 |
| 906 | POLR2A | 2.621978579 | 1.23833947 | 5.551605063 | 0.011785858 |
| 907 | POLR2J | 0.389917343 | 0.15828575 | 0.960513089 | 0.040604035 |
| 908 | POLR2J4 | 0.311516751 | 0.121240204 | 0.800416717 | 0.015421007 |
| 909 | POLR2K | 0.274221056 | 0.115045911 | 0.65362764 | 0.00350652 |
| 910 | POLR3F | 0.463077843 | 0.256629161 | 0.835606863 | 0.010578744 |
| 911 | POTEF | 2.912052913 | 1.232025953 | 6.883014233 | 0.014875495 |
| 912 | POU5F1 | 0.576486328 | 0.35487442 | 0.936490397 | 0.026079465 |
| 913 | PPA1 | 0.268388779 | 0.116470082 | 0.618463862 | 0.002014206 |
| 914 | PPARD | 2.78380546 | 1.15673954 | 6.699496796 | 0.022317494 |
| 915 | PPM1K | 2.212525305 | 1.054275706 | 4.643252422 | 0.035754249 |
| 916 | PPME1 | 3.571009545 | 1.406294195 | 9.067881537 | 0.007426881 |
| 917 | PPP2R5A | 0.210294307 | 0.073316606 | 0.603187979 | 0.003728488 |
| 918 | PPP2R5B | 6.70326875 | 2.034298486 | 22.08811157 | 0.001764765 |
| 919 | PRDM1 | 2.084080699 | 1.057103364 | 4.10876789 | 0.033979966 |
| 920 | PRDM13 | 0.737883519 | 0.552508894 | 0.985453979 | 0.039472287 |
| 921 | PRDM4 | 0.145254063 | 0.029874567 | 0.706244296 | 0.016802831 |
| 922 | PRDX4 | 0.42275135 | 0.1926068 | 0.927894052 | 0.031829353 |
| 923 | PRKACA | 3.706150408 | 1.106754121 | 12.41066158 | 0.033631804 |
| 924 | PRKCD | 5.80153837 | 1.689242178 | 19.92482067 | 0.005225648 |
| 925 | PRKCSH | 2.299129977 | 1.127379973 | 4.688746273 | 0.022037798 |
| 926 | PRKRA | 0.236763514 | 0.060916311 | 0.920229088 | 0.037526832 |
| 927 | PRKRIR | 0.326102293 | 0.117208573 | 0.907294603 | 0.031848144 |
| 928 | PRR24 | 3.181160034 | 1.248438453 | 8.105949589 | 0.015311507 |
| 929 | PRSS37 | 0.745339628 | 0.5830746 | 0.952761723 | 0.01896324 |
| 930 | PSMA2 | 0.252645544 | 0.086184452 | 0.740618166 | 0.012170225 |
| 931 | PSMA3 | 0.287352209 | 0.102850392 | 0.802829141 | 0.017364134 |
| 932 | PSMB7 | 0.493374933 | 0.284568539 | 0.855396119 | 0.011860708 |
| 933 | PSMC6 | 0.287102261 | 0.103985708 | 0.792683052 | 0.016025333 |
| 934 | PSMD12 | 0.27107796 | 0.084609125 | 0.868502786 | 0.02800032 |
| 935 | PSMD14 | 0.243116034 | 0.067262902 | 0.878722208 | 0.030992367 |
| 936 | PSMD5 | 0.298903577 | 0.121880495 | 0.733040575 | 0.008328082 |
| 937 | PTBP3 | 0.273428576 | 0.086393844 | 0.865376317 | 0.027388327 |
| 938 | PTF1A | 0.770429041 | 0.619436273 | 0.958227558 | 0.019111281 |
| 939 | PTOV1 | 4.107738905 | 1.282027346 | 13.16159049 | 0.017400137 |
| 940 | PTPN13 | 0.700719432 | 0.532184776 | 0.922626398 | 0.011287324 |
| 941 | PTPN23 | 3.425289698 | 1.200914224 | 9.769731497 | 0.021316489 |
| 942 | PTPN3 | 0.356062636 | 0.161551598 | 0.784768476 | 0.010435551 |
| 943 | PTPN9 | 5.497273502 | 1.659767853 | 18.20737515 | 0.005283867 |
| 944 | PTPRVP | 2.510041579 | 1.461746605 | 4.31012373 | 0.000849437 |
| 945 | PTRH1 | 0.481274971 | 0.23641237 | 0.979752445 | 0.04376205 |
| 946 | PTTG1IP | 2.82883489 | 1.210478288 | 6.610863589 | 0.016349785 |
| 947 | PTX4 | 0.714765421 | 0.516167503 | 0.989774837 | 0.043192235 |
| 948 | PUS7 | 0.440870189 | 0.215528084 | 0.901815301 | 0.024897157 |
| 949 | PXDN | 1.522594266 | 1.00363512 | 2.309896549 | 0.04803854 |
| 950 | QSOX1 | 2.982912081 | 1.487833411 | 5.980349964 | 0.002073394 |
| 951 | R3HDM1 | 0.390830044 | 0.181359995 | 0.842237139 | 0.016473483 |
| 952 | RAB11FIP3 | 2.429255721 | 1.020873606 | 5.780620954 | 0.044784848 |
| 953 | RAB22A | 0.512780789 | 0.26989605 | 0.97424226 | 0.041384825 |
| 954 | RAB30 | 3.098069288 | 1.283450024 | 7.47830701 | 0.011903253 |
| 955 | RAB33B | 0.673320213 | 0.458594419 | 0.988586192 | 0.043534267 |
| 956 | RAB3GAP2 | 0.398034467 | 0.170745559 | 0.927880281 | 0.032899839 |
| 957 | RABGEF1 | 0.235767712 | 0.072708676 | 0.764508678 | 0.016068969 |
| 958 | RABL5 | 0.309037415 | 0.095747827 | 0.997454735 | 0.04950379 |
| 959 | RACGAP1 | 0.404893455 | 0.180335399 | 0.909076701 | 0.028454302 |
| 960 | RAG1 | 0.528308153 | 0.300107686 | 0.930031179 | 0.02701139 |
| 961 | RALA | 0.237372642 | 0.075670116 | 0.744623826 | 0.013682432 |
| 962 | RAP1GAP2 | 0.38307663 | 0.206766731 | 0.709725901 | 0.002290125 |
| 963 | RASD1 | 1.600782678 | 1.083581687 | 2.364847261 | 0.018120516 |
| 964 | RASGRP3 | 2.06629918 | 1.046792317 | 4.078738667 | 0.036459309 |
| 965 | RASSF7 | 2.167407432 | 1.082093751 | 4.341264302 | 0.029066726 |
| 966 | RBBP8 | 0.507868006 | 0.273955769 | 0.941502025 | 0.031447271 |
| 967 | RBM11 | 0.748254146 | 0.578038149 | 0.968593974 | 0.027645658 |
| 968 | RBM17 | 0.153972368 | 0.035999326 | 0.658553727 | 0.0116256 |
| 969 | RBM18 | 0.238097368 | 0.0634365 | 0.893655181 | 0.033455244 |
| 970 | RBM34 | 0.213691965 | 0.071533537 | 0.638361503 | 0.005712654 |
| 971 | RBMXL1 | 0.425203786 | 0.202001034 | 0.89503631 | 0.024323869 |
| 972 | RCOR2 | 1.800441521 | 1.130211936 | 2.868125497 | 0.013315994 |
| 973 | RELA | 2.731256337 | 1.116595898 | 6.680806537 | 0.0276923 |
| 974 | REXO4 | 0.193299394 | 0.05396384 | 0.692401723 | 0.011582299 |
| 975 | RFC2 | 0.404422582 | 0.189982082 | 0.860910792 | 0.018850407 |
| 976 | RFK | 0.313676322 | 0.127721637 | 0.77036935 | 0.011437282 |
| 977 | RFWD2 | 0.180027861 | 0.042464141 | 0.76323293 | 0.019987353 |
| 978 | RG9MTD1 | 0.184966015 | 0.058468436 | 0.58514352 | 0.004079247 |
| 979 | RGP1 | 3.71903557 | 1.044634333 | 13.24025559 | 0.042625105 |
| 980 | RHEB | 0.201828847 | 0.068922332 | 0.591025904 | 0.003508317 |
| 981 | RHOT2 | 3.019908012 | 1.047983941 | 8.702274955 | 0.040681763 |
| 982 | RHOXF2 | 0.776737984 | 0.604696295 | 0.997727127 | 0.047953934 |
| 983 | RIMKLA | 1.824102811 | 1.17857414 | 2.82320047 | 0.006991541 |
| 984 | RIPK2 | 0.370107907 | 0.163253956 | 0.839059989 | 0.01730537 |
| 985 | RMI1 | 0.225948758 | 0.061601221 | 0.828763465 | 0.024882911 |
| 986 | RNF152 | 2.101978099 | 1.345133989 | 3.284663064 | 0.001107103 |
| 987 | RNF32 | 0.357448025 | 0.140319816 | 0.910556283 | 0.031055183 |
| 988 | RNH1 | 3.037702833 | 1.186172798 | 7.779337478 | 0.020568542 |
| 989 | ROPN1 | 0.582952683 | 0.400229558 | 0.849097285 | 0.004915698 |
| 990 | RPAP2 | 0.359568216 | 0.1448882 | 0.892338387 | 0.027412755 |
| 991 | RPAP3 | 0.449050957 | 0.219587633 | 0.918297444 | 0.028272655 |
| 992 | RPGRIP1L | 0.480423588 | 0.299521222 | 0.770585879 | 0.002357876 |
| 993 | RPL23AP53 | 0.178062467 | 0.042882336 | 0.739377677 | 0.017517985 |
| 994 | RPP38 | 0.187279832 | 0.058886483 | 0.59561607 | 0.004543506 |
| 995 | RPS6KL1 | 0.477267548 | 0.238316227 | 0.955806977 | 0.036840629 |
| 996 | RRBP1 | 1.904261812 | 1.039834769 | 3.487297365 | 0.036933153 |
| 997 | RRNAD1 | 3.606804216 | 1.232498882 | 10.55500888 | 0.019204843 |
| 998 | RRP15 | 0.293973428 | 0.150484813 | 0.574279718 | 0.000339199 |
| 999 | RRS1 | 0.464166781 | 0.217506655 | 0.990548086 | 0.047197945 |
| 1000 | RSRC1 | 0.343244957 | 0.14394458 | 0.818489312 | 0.015878088 |
| 1001 | RSU1 | 0.355143352 | 0.163604227 | 0.770926298 | 0.008848549 |
| 1002 | RTKN2 | 0.344706489 | 0.169183871 | 0.702327962 | 0.003356341 |
| 1003 | RUVBL1 | 0.416344163 | 0.193229704 | 0.897079789 | 0.025268403 |
| 1004 | RYBP | 3.640415639 | 1.006010181 | 13.1734512 | 0.048942091 |
| 1005 | SAMD15 | 0.698177339 | 0.525507944 | 0.927581784 | 0.013191164 |
| 1006 | SCAI | 0.41364935 | 0.202259364 | 0.845972131 | 0.01559823 |
| 1007 | SCAMP2 | 2.277640455 | 1.105633985 | 4.692010298 | 0.025595648 |
| 1008 | SCLT1 | 0.395401868 | 0.214789659 | 0.72788717 | 0.002881984 |
| 1009 | SCN1B | 0.673313 | 0.460458099 | 0.984563844 | 0.04133002 |
| 1010 | SCYL1 | 2.863353747 | 1.212198217 | 6.763575928 | 0.016450913 |
| 1011 | SDC4P | 0.725018378 | 0.54417305 | 0.965964134 | 0.028055789 |
| 1012 | SDCCAG3 | 0.361741128 | 0.171383432 | 0.763531466 | 0.0076341 |
| 1013 | SDF4 | 3.509285739 | 1.393144023 | 8.8397798 | 0.00773597 |
| 1014 | SEC14L1 | 3.034108446 | 1.276617533 | 7.211097938 | 0.011975425 |
| 1015 | SEC24D | 2.62710138 | 1.055023253 | 6.54171521 | 0.037983313 |
| 1016 | SEC31A | 6.282948689 | 1.526086397 | 25.86710968 | 0.010914823 |
| 1017 | SEMA3B | 1.61680547 | 1.037930634 | 2.518530471 | 0.03362059 |
| 1018 | SEMA3C | 0.592066447 | 0.377822877 | 0.927796328 | 0.022197998 |
| 1019 | SEMA3E | 1.351453371 | 1.106653991 | 1.650404037 | 0.003138006 |
| 1020 | SEMA4B | 2.715154174 | 1.030494199 | 7.153909452 | 0.043307335 |
| 1021 | SEPN1 | 4.980926416 | 1.295991835 | 19.14335205 | 0.019417811 |
| 1022 | SEPT9 | 2.328362426 | 1.009131924 | 5.372212944 | 0.047560521 |
| 1023 | SET | 0.113360401 | 0.032459712 | 0.395893231 | 0.000644413 |
| 1024 | SETP20 | 0.374492033 | 0.156596057 | 0.895579915 | 0.02725311 |
| 1025 | SEZ6L2 | 2.067427319 | 1.262598301 | 3.3852855 | 0.003892955 |
| 1026 | SF3A2 | 2.856657715 | 1.132918419 | 7.203072313 | 0.026119181 |
| 1027 | SF3B14 | 0.203896617 | 0.067466091 | 0.616218159 | 0.004833098 |
| 1028 | SGOL2 | 0.468343087 | 0.261112594 | 0.840040857 | 0.0109372 |
| 1029 | SGSH | 2.520764945 | 1.075047998 | 5.910671819 | 0.03347025 |
| 1030 | SH2D3C | 1.798632673 | 1.028669261 | 3.144917045 | 0.03948346 |
| 1031 | SH3BGRL3 | 2.498642788 | 1.014510809 | 6.153917463 | 0.046448909 |
| 1032 | SHBG | 0.625865131 | 0.414218974 | 0.945652389 | 0.026059738 |
| 1033 | SIAH1 | 3.568077509 | 1.086516995 | 11.71742105 | 0.03601669 |
| 1034 | SIDT2 | 3.035591058 | 1.15248148 | 7.995627894 | 0.024629346 |
| 1035 | SKA2 | 0.349354759 | 0.142089146 | 0.858958976 | 0.021952462 |
| 1036 | SKI | 2.265162621 | 1.157221935 | 4.433861426 | 0.017028462 |
| 1037 | SLC11A2 | 2.023467727 | 1.027211034 | 3.985959563 | 0.041592107 |
| 1038 | SLC16A3 | 1.51783779 | 1.03181678 | 2.232791324 | 0.034089196 |
| 1039 | SLC22A11 | 0.714659913 | 0.530712631 | 0.962364114 | 0.026923388 |
| 1040 | SLC22A18 | 1.969582616 | 1.205449403 | 3.218099137 | 0.006812271 |
| 1041 | SLC22A3 | 0.72399306 | 0.577420158 | 0.907772171 | 0.005136648 |
| 1042 | SLC25A22 | 1.938066699 | 1.030966817 | 3.643281694 | 0.039911625 |
| 1043 | SLC25A43 | 0.371801772 | 0.146005998 | 0.946786843 | 0.03802148 |
| 1044 | SLC26A6 | 2.511369535 | 1.030913884 | 6.117850425 | 0.042664167 |
| 1045 | SLC2A5 | 4.247445864 | 1.333640075 | 13.5274852 | 0.014401219 |
| 1046 | SLC35E2 | 4.054938642 | 1.39727902 | 11.7675333 | 0.01001325 |
| 1047 | SLC37A3 | 0.355695534 | 0.146521362 | 0.863487151 | 0.022352495 |
| 1048 | SLC39A7 | 2.86589453 | 1.39016845 | 5.908169947 | 0.004338609 |
| 1049 | SLC43A3 | 1.588474212 | 1.065688123 | 2.367719288 | 0.02306471 |
| 1050 | SLC5A12 | 0.468196135 | 0.22467957 | 0.975645542 | 0.042787079 |
| 1051 | SLC7A13 | 0.573168818 | 0.361072795 | 0.90985114 | 0.018241993 |
| 1052 | SLC7A3 | 0.687688283 | 0.511567679 | 0.92444303 | 0.013122471 |
| 1053 | SLC7A9 | 1.327183544 | 1.006353043 | 1.750296451 | 0.04498248 |
| 1054 | SLC9A3R1 | 1.932874418 | 1.055161886 | 3.540692253 | 0.032857354 |
| 1055 | SLCO1B3 | 0.808530591 | 0.708209425 | 0.923062717 | 0.001664343 |
| 1056 | SLITRK5 | 1.298920296 | 1.02744261 | 1.642129612 | 0.028795265 |
| 1057 | SLMO2 | 0.339217861 | 0.146689245 | 0.784438949 | 0.011484951 |
| 1058 | SMAD3 | 2.897585487 | 1.136474688 | 7.387759483 | 0.025889571 |
| 1059 | SMAD7 | 2.672797276 | 1.025351727 | 6.967214368 | 0.0443065 |
| 1060 | SMC2 | 0.476143813 | 0.255394191 | 0.887698072 | 0.01955486 |
| 1061 | SMC6 | 0.507753706 | 0.260097061 | 0.991221606 | 0.047055783 |
| 1062 | SMEK2 | 0.417602917 | 0.208332673 | 0.837085195 | 0.013848295 |
| 1063 | SMG8 | 0.202344431 | 0.048530488 | 0.843660764 | 0.0282832 |
| 1064 | SMYD1 | 0.731842117 | 0.536005382 | 0.999230422 | 0.049436315 |
| 1065 | SMYD2 | 0.254510705 | 0.091252014 | 0.709855005 | 0.008928176 |
| 1066 | SMYD3 | 0.227891705 | 0.072112933 | 0.720184671 | 0.01176579 |
| 1067 | SNAP47 | 0.184695743 | 0.043116583 | 0.791169323 | 0.022873239 |
| 1068 | SNAPC2 | 2.994158559 | 1.292203367 | 6.937751209 | 0.010531353 |
| 1069 | SNAPC5 | 0.357183768 | 0.139556605 | 0.914182775 | 0.031786119 |
| 1070 | SNHG5 | 0.711647957 | 0.511739102 | 0.989650415 | 0.043197347 |
| 1071 | SNHG7 | 0.358414874 | 0.154025518 | 0.83402558 | 0.017259019 |
| 1072 | SNTN | 0.691042361 | 0.536498658 | 0.890103895 | 0.004218459 |
| 1073 | SNX16 | 0.598225512 | 0.364091592 | 0.982922352 | 0.042565066 |
| 1074 | SNX2 | 0.303995879 | 0.112369507 | 0.822407227 | 0.019026298 |
| 1075 | SNX33 | 9.653111236 | 2.808440174 | 33.17947001 | 0.000319166 |
| 1076 | SNX9 | 2.701606745 | 1.222629418 | 5.969657604 | 0.014015742 |
| 1077 | SOCS4 | 0.431453525 | 0.193730215 | 0.960883381 | 0.039624895 |
| 1078 | SORL1 | 2.310781194 | 1.214085156 | 4.39813443 | 0.010749704 |
| 1079 | SPA17 | 0.358782564 | 0.167237899 | 0.769711467 | 0.008487223 |
| 1080 | SPAG6 | 1.682493386 | 1.074282003 | 2.635047395 | 0.02302607 |
| 1081 | SPANXN4 | 0.599482251 | 0.382354365 | 0.939910727 | 0.025744563 |
| 1082 | SPAST | 0.497805109 | 0.297648423 | 0.832559179 | 0.007852954 |
| 1083 | SPATA17 | 0.703395491 | 0.52695475 | 0.938914048 | 0.016953055 |
| 1084 | SPATA5 | 0.637308372 | 0.48163331 | 0.843301226 | 0.001617908 |
| 1085 | SPATA8 | 0.771417827 | 0.606483903 | 0.981205704 | 0.034468061 |
| 1086 | SPC25 | 0.51363461 | 0.274005554 | 0.96282907 | 0.037698709 |
| 1087 | SPDYA | 0.719723001 | 0.533911417 | 0.97020064 | 0.030888072 |
| 1088 | SPINT1 | 2.455887326 | 1.121388686 | 5.378494212 | 0.024677926 |
| 1089 | SRBD1 | 0.314435955 | 0.102993456 | 0.95996361 | 0.042182375 |
| 1090 | SREK1IP1 | 0.432080404 | 0.188579353 | 0.989999551 | 0.047286626 |
| 1091 | SRFBP1 | 0.28398807 | 0.107475922 | 0.750393416 | 0.01111079 |
| 1092 | SRL | 1.607114457 | 1.048552012 | 2.46322247 | 0.029438429 |
| 1093 | SRP9 | 0.205734904 | 0.073093555 | 0.579077737 | 0.002747411 |
| 1094 | SRPK2 | 0.260924121 | 0.099948723 | 0.681163248 | 0.006065791 |
| 1095 | SRSF4 | 4.227303019 | 1.060758917 | 16.84651483 | 0.04099492 |
| 1096 | SSBP1 | 0.301341232 | 0.111600387 | 0.813675836 | 0.017941969 |
| 1097 | SSX2IP | 0.437434109 | 0.204882509 | 0.933943073 | 0.032634083 |
| 1098 | SSX4B | 0.743440025 | 0.561874893 | 0.983676397 | 0.037971123 |
| 1099 | ST14 | 3.025003889 | 1.302160007 | 7.027284268 | 0.010055964 |
| 1100 | ST5 | 5.459635815 | 1.851342737 | 16.10054294 | 0.002096656 |
| 1101 | STAB1 | 1.868127846 | 1.027606039 | 3.396147468 | 0.040436566 |
| 1102 | STAM | 0.237914175 | 0.076769165 | 0.737316275 | 0.012846023 |
| 1103 | STAMBPL1 | 0.318067084 | 0.146131129 | 0.692300608 | 0.003893512 |
| 1104 | STAR | 0.639590772 | 0.42701937 | 0.957980795 | 0.030141728 |
| 1105 | STAU2 | 0.557887209 | 0.314868597 | 0.988469922 | 0.045532522 |
| 1106 | STIM1 | 3.647553119 | 1.292719501 | 10.29198039 | 0.014481917 |
| 1107 | STOML2 | 0.522289203 | 0.272975015 | 0.999307616 | 0.049755941 |
| 1108 | STRBP | 0.248751203 | 0.105240577 | 0.587959157 | 0.001524058 |
| 1109 | STX17 | 0.120449551 | 0.03717156 | 0.390300926 | 0.000418057 |
| 1110 | STX5 | 2.424868274 | 1.029324095 | 5.712473043 | 0.042757251 |
| 1111 | STX6 | 0.251674843 | 0.085630901 | 0.739688893 | 0.012137007 |
| 1112 | STYX | 0.353097157 | 0.135981663 | 0.916870701 | 0.032498303 |
| 1113 | SUMO1P3 | 0.294411365 | 0.10291196 | 0.842254405 | 0.022603059 |
| 1114 | SUPT5H | 10.78940299 | 2.284101463 | 50.96586938 | 0.002676338 |
| 1115 | SUPT6H | 3.48189666 | 1.189113682 | 10.19549647 | 0.022849207 |
| 1116 | SUPV3L1 | 0.339446803 | 0.126441601 | 0.911283402 | 0.032005232 |
| 1117 | SUV39H2 | 0.185780013 | 0.068596412 | 0.503148961 | 0.000929065 |
| 1118 | SVIP | 0.422583329 | 0.201918664 | 0.884399026 | 0.02225498 |
| 1119 | SWI5 | 0.261255609 | 0.070897042 | 0.962726952 | 0.043690736 |
| 1120 | SYNGR4 | 1.786977275 | 1.013241641 | 3.151556007 | 0.044919597 |
| 1121 | SYNPO | 3.706146133 | 1.69595242 | 8.099000301 | 0.001022161 |
| 1122 | SYPL1 | 0.203628478 | 0.067029816 | 0.618598704 | 0.004998072 |
| 1123 | SYVN1 | 6.708809317 | 2.27313512 | 19.80002071 | 0.000566689 |
| 1124 | TAF15 | 2.679844069 | 1.190619773 | 6.031786465 | 0.017243615 |
| 1125 | TAF1A | 0.435327715 | 0.21139614 | 0.896469632 | 0.024039437 |
| 1126 | TAF1B | 0.552737115 | 0.31413137 | 0.97258137 | 0.039744523 |
| 1127 | TAF9 | 0.259650117 | 0.084686949 | 0.796087054 | 0.018328928 |
| 1128 | TAF9B | 0.35203467 | 0.171362253 | 0.723195491 | 0.004480123 |
| 1129 | TAS2R3 | 0.704746038 | 0.506671778 | 0.980253885 | 0.037670283 |
| 1130 | TATDN3 | 0.241006422 | 0.084878095 | 0.68432374 | 0.007531983 |
| 1131 | TBC1D2B | 4.018304129 | 1.400901332 | 11.52598524 | 0.009681576 |
| 1132 | TBK1 | 0.192960147 | 0.065912295 | 0.564896403 | 0.002681722 |
| 1133 | TBX2 | 1.71257991 | 1.037858122 | 2.825944977 | 0.035258234 |
| 1134 | TCF24 | 0.696397775 | 0.537824493 | 0.901725131 | 0.006057867 |
| 1135 | TCF25 | 3.844661764 | 1.097908719 | 13.46325411 | 0.03520082 |
| 1136 | TCF7L1 | 2.219474114 | 1.157485124 | 4.255834688 | 0.016383274 |
| 1137 | TCIRG1 | 2.815314376 | 1.251895587 | 6.331194966 | 0.012304308 |
| 1138 | TCN2 | 2.129867372 | 1.148069018 | 3.951273794 | 0.016489392 |
| 1139 | TCP10 | 0.538861605 | 0.290517713 | 0.999497851 | 0.049814029 |
| 1140 | TCTN1 | 0.192676037 | 0.057136907 | 0.649738621 | 0.007926019 |
| 1141 | TDGF1 | 0.669461871 | 0.493065109 | 0.908965548 | 0.010121415 |
| 1142 | TENC1 | 2.678747209 | 1.218900402 | 5.887016363 | 0.014179213 |
| 1143 | TERF1 | 0.525571616 | 0.288931972 | 0.956022699 | 0.035092126 |
| 1144 | TESC | 0.734572276 | 0.541788749 | 0.995953552 | 0.047025555 |
| 1145 | TEX10 | 0.231306473 | 0.086138647 | 0.621122881 | 0.003673794 |
| 1146 | TFAM | 0.253194059 | 0.076153299 | 0.841818181 | 0.025034178 |
| 1147 | TGFB1 | 1.645306736 | 1.013316117 | 2.671460769 | 0.044066817 |
| 1148 | TGFB1I1 | 2.305885382 | 1.143615843 | 4.649382418 | 0.019542225 |
| 1149 | THAP1 | 0.326956426 | 0.156876602 | 0.681430522 | 0.002848272 |
| 1150 | THAP10 | 0.511861165 | 0.26990119 | 0.970732484 | 0.040273545 |
| 1151 | THAP5 | 0.295953376 | 0.112162935 | 0.780903254 | 0.013911773 |
| 1152 | THAP9 | 0.526651745 | 0.278993806 | 0.994151322 | 0.047922018 |
| 1153 | THBS1 | 1.958578902 | 1.073448902 | 3.573557444 | 0.028453706 |
| 1154 | THOC7 | 0.382317364 | 0.147120356 | 0.993516945 | 0.048459959 |
| 1155 | THUMPD1 | 0.45416177 | 0.217231299 | 0.949508263 | 0.035935401 |
| 1156 | TIGD4 | 0.669022242 | 0.494467429 | 0.90519766 | 0.009169826 |
| 1157 | TIMP4 | 1.474461733 | 1.022432216 | 2.126338909 | 0.037642378 |
| 1158 | TLN2 | 0.510616809 | 0.261780911 | 0.99598372 | 0.048635889 |
| 1159 | TLR4 | 0.550847295 | 0.333492615 | 0.909863454 | 0.019865004 |
| 1160 | TMCO7 | 0.381701832 | 0.182370169 | 0.798904169 | 0.010596267 |
| 1161 | TMEM117 | 0.38925321 | 0.157578673 | 0.961539136 | 0.040857987 |
| 1162 | TMEM198 | 1.840903051 | 1.176842867 | 2.879674201 | 0.00751141 |
| 1163 | TMEM206 | 0.520783513 | 0.280304468 | 0.967574541 | 0.038993237 |
| 1164 | TMEM211 | 0.666792359 | 0.491958896 | 0.903758531 | 0.008996114 |
| 1165 | TMEM237 | 0.300839477 | 0.122566892 | 0.738408146 | 0.008743813 |
| 1166 | TMEM5 | 0.199801157 | 0.053209269 | 0.750254674 | 0.017050365 |
| 1167 | TMEM60 | 0.241662309 | 0.100257448 | 0.582507062 | 0.001556891 |
| 1168 | TMEM65 | 1.824636337 | 1.001379657 | 3.324710802 | 0.049475883 |
| 1169 | TMEM67 | 0.525171168 | 0.316384195 | 0.871739993 | 0.012743993 |
| 1170 | TMEM80 | 3.278164786 | 1.37663079 | 7.806279243 | 0.007318086 |
| 1171 | TMPPE | 2.565414135 | 1.001909491 | 6.568806609 | 0.049536968 |
| 1172 | TMPRSS11B | 0.771506044 | 0.605218342 | 0.98348238 | 0.036220972 |
| 1173 | TNFRSF21 | 1.93425379 | 1.023244909 | 3.656346287 | 0.0422859 |
| 1174 | TNIP1 | 5.069211186 | 1.987038745 | 12.93226018 | 0.000681399 |
| 1175 | TNS1 | 1.745206038 | 1.057240118 | 2.88084425 | 0.029433792 |
| 1176 | TOLLIP | 3.242216677 | 1.467763103 | 7.161897557 | 0.003626047 |
| 1177 | TOM1L2 | 2.894475242 | 1.069744556 | 7.831764019 | 0.036374395 |
| 1178 | TOMM20 | 0.28007739 | 0.104172939 | 0.753010765 | 0.01166451 |
| 1179 | TOMM5 | 0.391311576 | 0.16317893 | 0.938385549 | 0.035512241 |
| 1180 | TOP2A | 0.543728 | 0.334938065 | 0.882671064 | 0.013707875 |
| 1181 | TOR1A | 0.249725705 | 0.063847816 | 0.976743314 | 0.046176075 |
| 1182 | TP53INP1 | 2.034611201 | 1.056745549 | 3.917350532 | 0.033578809 |
| 1183 | TP53TG1 | 0.51784339 | 0.275130784 | 0.974670201 | 0.04140261 |
| 1184 | TPCN1 | 3.052160834 | 1.242223822 | 7.499200702 | 0.014979441 |
| 1185 | TPR | 0.57161596 | 0.331305363 | 0.986234579 | 0.044455226 |
| 1186 | TPRG1 | 0.774967061 | 0.622322532 | 0.965052549 | 0.022738468 |
| 1187 | TRAF5 | 0.553963605 | 0.331524981 | 0.925648727 | 0.02413853 |
| 1188 | TRDMT1 | 0.301681944 | 0.135380129 | 0.672269969 | 0.003375821 |
| 1189 | TRDN | 1.592318028 | 1.120194785 | 2.263424839 | 0.009527685 |
| 1190 | TRIAP1 | 0.274363921 | 0.094434033 | 0.797123231 | 0.017470839 |
| 1191 | TRIM24 | 0.445270321 | 0.260830482 | 0.760132241 | 0.003026072 |
| 1192 | TRIM3 | 4.329615008 | 1.489231199 | 12.58741163 | 0.007115819 |
| 1193 | TRIM37 | 0.398366701 | 0.194700796 | 0.815076424 | 0.011743603 |
| 1194 | TRIM48 | 0.739291217 | 0.560560111 | 0.975009625 | 0.032420029 |
| 1195 | TRIM5 | 2.809445736 | 1.07107572 | 7.369213207 | 0.035771015 |
| 1196 | TRIM59 | 0.394425052 | 0.163021228 | 0.954299781 | 0.039043352 |
| 1197 | TRIM72 | 0.73430498 | 0.548881413 | 0.982368489 | 0.037547638 |
| 1198 | TRMT6 | 0.55409244 | 0.312687785 | 0.981868967 | 0.043109614 |
| 1199 | TRMT61B | 0.346548855 | 0.168459361 | 0.712908491 | 0.003983625 |
| 1200 | TRPV6 | 1.613939142 | 1.084570005 | 2.401688727 | 0.018261937 |
| 1201 | TRUB1 | 0.321821197 | 0.112336797 | 0.921949757 | 0.034746922 |
| 1202 | TRUB2 | 0.181556558 | 0.055789765 | 0.590839268 | 0.004596776 |
| 1203 | TSEN15 | 0.31674284 | 0.145587027 | 0.689113785 | 0.003745662 |
| 1204 | TSG1 | 0.720200686 | 0.530366301 | 0.977982628 | 0.035502407 |
| 1205 | TSPAN4 | 1.862647531 | 1.002886173 | 3.459471193 | 0.048943021 |
| 1206 | TSSC1 | 0.41456991 | 0.209916316 | 0.818746314 | 0.011215298 |
| 1207 | TTC18 | 0.743870589 | 0.571846696 | 0.967643 | 0.027447764 |
| 1208 | TTC25 | 0.532357039 | 0.323594983 | 0.875798548 | 0.013061196 |
| 1209 | TTC26 | 0.494707053 | 0.29286059 | 0.835670886 | 0.008511009 |
| 1210 | TTC27 | 0.28921248 | 0.109576775 | 0.763335645 | 0.012233527 |
| 1211 | TTC7A | 3.157791892 | 1.257798183 | 7.927861376 | 0.014352156 |
| 1212 | TTF1 | 0.401544023 | 0.206830836 | 0.77956269 | 0.007024869 |
| 1213 | TTK | 0.472240698 | 0.245813713 | 0.907236926 | 0.024309589 |
| 1214 | TUBD1 | 0.306733361 | 0.11514856 | 0.817077997 | 0.018073995 |
| 1215 | TUT1 | 3.268138667 | 1.112612413 | 9.599686485 | 0.031234818 |
| 1216 | TWF1 | 0.321612243 | 0.131208903 | 0.788318723 | 0.01314064 |
| 1217 | TWF2 | 3.052959274 | 1.218021787 | 7.652211502 | 0.017282199 |
| 1218 | TWISTNB | 0.221260985 | 0.08785355 | 0.557250373 | 0.001370747 |
| 1219 | TXK | 0.505750797 | 0.30431911 | 0.840512017 | 0.008529802 |
| 1220 | TXNDC11 | 2.392184816 | 1.009550953 | 5.668409479 | 0.047528536 |
| 1221 | TXNDC15 | 3.370915456 | 1.061184122 | 10.70791654 | 0.039334757 |
| 1222 | TXNDC9 | 0.274635409 | 0.078984921 | 0.95492414 | 0.042102522 |
| 1223 | TYW3 | 0.442682861 | 0.236729342 | 0.827815064 | 0.010721027 |
| 1224 | U2AF1L4 | 2.256727106 | 1.006089546 | 5.061991998 | 0.04830295 |
| 1225 | UBA6 | 0.526969919 | 0.290210793 | 0.956881348 | 0.035310583 |
| 1226 | UBA7 | 2.207406762 | 1.056647617 | 4.611418727 | 0.035156125 |
| 1227 | UBE2CBP | 0.233038161 | 0.068652785 | 0.791035419 | 0.019496732 |
| 1228 | UBE2N | 0.394940501 | 0.177260942 | 0.879934397 | 0.023032353 |
| 1229 | UBE2Q1 | 0.366663822 | 0.156852882 | 0.857123926 | 0.020567936 |
| 1230 | UBE2V2 | 0.475646517 | 0.264757596 | 0.854516029 | 0.012921154 |
| 1231 | UBE3C | 0.167613085 | 0.038127575 | 0.736845869 | 0.018070159 |
| 1232 | UBLCP1 | 0.307402695 | 0.114183209 | 0.827585929 | 0.019570359 |
| 1233 | UBN1 | 5.469145564 | 1.136035497 | 26.32976987 | 0.034088032 |
| 1234 | UBXN2B | 0.498452976 | 0.248579014 | 0.999502593 | 0.049836395 |
| 1235 | UBXN8 | 0.548912786 | 0.320638668 | 0.939703398 | 0.028765355 |
| 1236 | UCHL5 | 0.24581657 | 0.072603453 | 0.83227152 | 0.024132062 |
| 1237 | ULK1 | 2.944891984 | 1.145055569 | 7.57377112 | 0.025025295 |
| 1238 | UNC5B | 1.914473028 | 1.147920562 | 3.192909944 | 0.012825496 |
| 1239 | UQCRB | 0.622747592 | 0.392175178 | 0.988880953 | 0.044711913 |
| 1240 | URB2 | 0.449402539 | 0.209660355 | 0.963284841 | 0.039770646 |
| 1241 | USP14 | 0.473327591 | 0.259909645 | 0.861988053 | 0.01446344 |
| 1242 | USP4 | 3.192746132 | 1.029248924 | 9.903948043 | 0.044444178 |
| 1243 | USP6NL | 0.382657599 | 0.172966632 | 0.846561191 | 0.017734177 |
| 1244 | UTP15 | 0.352000521 | 0.149277303 | 0.830028172 | 0.017050709 |
| 1245 | UTP18 | 0.212955078 | 0.063344967 | 0.715918992 | 0.012413232 |
| 1246 | UTP3 | 0.234181933 | 0.078573193 | 0.697962947 | 0.009178615 |
| 1247 | VAMP4 | 0.299435426 | 0.122216796 | 0.733627265 | 0.008352911 |
| 1248 | VAMP7 | 0.270021979 | 0.111574715 | 0.653480216 | 0.00369087 |
| 1249 | VASH1 | 1.987651042 | 1.029880272 | 3.836131997 | 0.040586578 |
| 1250 | VBP1 | 0.347058169 | 0.13235543 | 0.910044819 | 0.031427923 |
| 1251 | VDAC3 | 0.379648767 | 0.173460312 | 0.830928902 | 0.015376061 |
| 1252 | VEGFA | 2.408599796 | 1.157704904 | 5.011080939 | 0.018685921 |
| 1253 | VIL1 | 2.547694031 | 1.073483451 | 6.04643217 | 0.033941401 |
| 1254 | VLDLR | 2.127347059 | 1.253430855 | 3.610574522 | 0.005159742 |
| 1255 | VPS26A | 0.16161815 | 0.048161576 | 0.542349911 | 0.003172814 |
| 1256 | VPS37A | 0.264035569 | 0.087650292 | 0.795374206 | 0.017938697 |
| 1257 | VPS37C | 4.215226415 | 1.405679667 | 12.64024382 | 0.010237469 |
| 1258 | VPS54 | 0.2892608 | 0.091664207 | 0.912807883 | 0.034382486 |
| 1259 | VRK2 | 0.34900484 | 0.149629777 | 0.81403836 | 0.014846176 |
| 1260 | VWA3A | 0.627277579 | 0.431186421 | 0.912545344 | 0.014749181 |
| 1261 | WASF2 | 2.689821173 | 1.009673386 | 7.165820195 | 0.047791195 |
| 1262 | WDHD1 | 0.480714333 | 0.249986694 | 0.92439428 | 0.028119335 |
| 1263 | WDR43 | 0.284607114 | 0.113217985 | 0.715444717 | 0.00754141 |
| 1264 | WDR6 | 3.308871363 | 1.057566385 | 10.35266424 | 0.039769186 |
| 1265 | WDR66 | 0.739443099 | 0.565882906 | 0.966235402 | 0.026992962 |
| 1266 | WDR75 | 0.260649179 | 0.081040916 | 0.83831721 | 0.024080219 |
| 1267 | WDR82 | 6.178869015 | 1.401168762 | 27.24755456 | 0.016150055 |
| 1268 | WDR85 | 0.385564586 | 0.155828868 | 0.953995568 | 0.039221673 |
| 1269 | WDR92 | 0.2334366 | 0.072992691 | 0.746549349 | 0.014177023 |
| 1270 | WEE1 | 2.372367392 | 1.051882182 | 5.350529879 | 0.037355456 |
| 1271 | WNT10A | 1.515359062 | 1.000669217 | 2.294777385 | 0.04963181 |
| 1272 | WRB | 0.400351164 | 0.178951514 | 0.895667495 | 0.025869687 |
| 1273 | WSB1 | 3.249458688 | 1.199598331 | 8.802097751 | 0.020454555 |
| 1274 | WWP1 | 0.510755012 | 0.261191357 | 0.998772263 | 0.049581802 |
| 1275 | XPA | 0.223656959 | 0.079355294 | 0.630360402 | 0.004613692 |
| 1276 | XPNPEP2 | 1.513430901 | 1.02451422 | 2.235667449 | 0.037376693 |
| 1277 | YEATS4 | 0.324972187 | 0.138949514 | 0.760038084 | 0.009516153 |
| 1278 | YES1 | 0.530579664 | 0.301041152 | 0.935137199 | 0.028387283 |
| 1279 | YME1L1 | 0.184995853 | 0.052672391 | 0.649742017 | 0.008471353 |
| 1280 | ZAK | 0.582440824 | 0.366570405 | 0.92543563 | 0.02213926 |
| 1281 | ZBTB33 | 0.458265544 | 0.231004762 | 0.909103808 | 0.025573582 |
| 1282 | ZBTB6 | 0.419187341 | 0.207127451 | 0.848357022 | 0.015641673 |
| 1283 | ZC3H15 | 0.152869144 | 0.050792415 | 0.460087898 | 0.000834991 |
| 1284 | ZC3H8 | 0.531758652 | 0.289254978 | 0.977570951 | 0.042054122 |
| 1285 | ZCCHC10 | 0.336849054 | 0.141743324 | 0.80051238 | 0.013748637 |
| 1286 | ZCCHC9 | 0.32952506 | 0.118612379 | 0.915475823 | 0.03322452 |
| 1287 | ZDHHC1 | 0.421016033 | 0.183198197 | 0.967555925 | 0.041584837 |
| 1288 | ZFAND1 | 0.427175678 | 0.190667365 | 0.957054501 | 0.038770346 |
| 1289 | ZFP1 | 0.287448709 | 0.127749751 | 0.646786076 | 0.00258624 |
| 1290 | ZFP161 | 0.314460238 | 0.112562448 | 0.878492279 | 0.027306076 |
| 1291 | ZFP90 | 0.243578521 | 0.080825887 | 0.734053142 | 0.012097879 |
| 1292 | ZFYVE27 | 2.518366114 | 1.143135055 | 5.548047762 | 0.021910075 |
| 1293 | ZMYND19 | 0.505656617 | 0.271336252 | 0.942331195 | 0.031794669 |
| 1294 | ZNF107 | 0.563157817 | 0.362393661 | 0.875144247 | 0.010682296 |
| 1295 | ZNF117 | 0.559554448 | 0.319609616 | 0.979636295 | 0.042157087 |
| 1296 | ZNF200 | 0.40260561 | 0.166596983 | 0.972954456 | 0.043293686 |
| 1297 | ZNF207 | 0.108903266 | 0.018928392 | 0.626567836 | 0.013005702 |
| 1298 | ZNF229 | 2.236692565 | 1.202647304 | 4.159817774 | 0.010995357 |
| 1299 | ZNF23 | 0.522177853 | 0.278211604 | 0.980080291 | 0.043114613 |
| 1300 | ZNF334 | 1.204476299 | 1.012660866 | 1.432624885 | 0.03554267 |
| 1301 | ZNF341 | 6.583306875 | 1.99433044 | 21.73156892 | 0.001982139 |
| 1302 | ZNF398 | 0.210426884 | 0.064380361 | 0.687779202 | 0.009897853 |
| 1303 | ZNF420 | 0.394162612 | 0.156466497 | 0.992954837 | 0.048272443 |
| 1304 | ZNF473 | 0.483786196 | 0.261522005 | 0.894949866 | 0.020689546 |
| 1305 | ZNF484 | 0.40616282 | 0.168570778 | 0.978628908 | 0.044631471 |
| 1306 | ZNF485 | 0.486733891 | 0.252898952 | 0.936776836 | 0.03112478 |
| 1307 | ZNF503-AS1 | 0.789007331 | 0.633715932 | 0.982352719 | 0.034074978 |
| 1308 | ZNF566 | 0.50018725 | 0.25760481 | 0.971205798 | 0.040730527 |
| 1309 | ZNF57 | 0.409952154 | 0.190449231 | 0.882443933 | 0.022626572 |
| 1310 | ZNF575 | 2.096576238 | 1.081799071 | 4.063260951 | 0.028317425 |
| 1311 | ZNF596 | 0.575040348 | 0.359940379 | 0.918683817 | 0.020625091 |
| 1312 | ZNF642 | 0.478674357 | 0.281774747 | 0.813164212 | 0.006431549 |
| 1313 | ZNF643 | 0.403518285 | 0.207709015 | 0.783918823 | 0.007395778 |
| 1314 | ZNF678 | 0.585117338 | 0.370050143 | 0.925178132 | 0.02186862 |
| 1315 | ZNF680 | 0.576229476 | 0.34243654 | 0.969640709 | 0.037887262 |
| 1316 | ZNF721 | 0.593904894 | 0.355980975 | 0.99084796 | 0.046024269 |
| 1317 | ZNF761 | 0.558588073 | 0.355210058 | 0.878411602 | 0.011694323 |
| 1318 | ZNF77 | 0.40089396 | 0.186743738 | 0.860623061 | 0.019024676 |
| 1319 | ZNF786 | 0.342052048 | 0.125485798 | 0.932373265 | 0.036009024 |
| 1320 | ZNF815 | 0.359249941 | 0.165025456 | 0.782064313 | 0.009900003 |
| 1321 | ZP1 | 2.138551089 | 1.200092852 | 3.810872427 | 0.009915637 |
| 1322 | ZP3 | 0.482176704 | 0.288343399 | 0.80631072 | 0.005425334 |
| 1323 | ZPBP2 | 0.614934343 | 0.411362852 | 0.919247434 | 0.017766788 |
| 1324 | ZUFSP | 0.243320153 | 0.071650607 | 0.826297215 | 0.023460892 |
| 1325 | ZWILCH | 0.518544129 | 0.275529516 | 0.975895496 | 0.041791217 |
| 1326 | ZWINT | 0.3440004 | 0.14874637 | 0.795557396 | 0.012608694 |
| 1327 | ZYG11A | 1.402307834 | 1.005977276 | 1.954782983 | 0.046028824 |

*Note:* **DFS:** Disease-free survival.
