# Supplementary material for: Deciphering colorectal cancer radioresistance and immune microrenvironment: unraveling the role of EIF5A through single-cell RNA sequencing and machine learning
Source: Front Immunol. 2024 Sep 3;15:1466226. doi: 10.3389/fimmu.2024.1466226 (PMC11405197; doi:10.3389/fimmu.2024.1466226)
Supplement: Supplementary file 2 [file DataSheet1.docx]

Supplementary Material

# Supplementary figures S1-9:


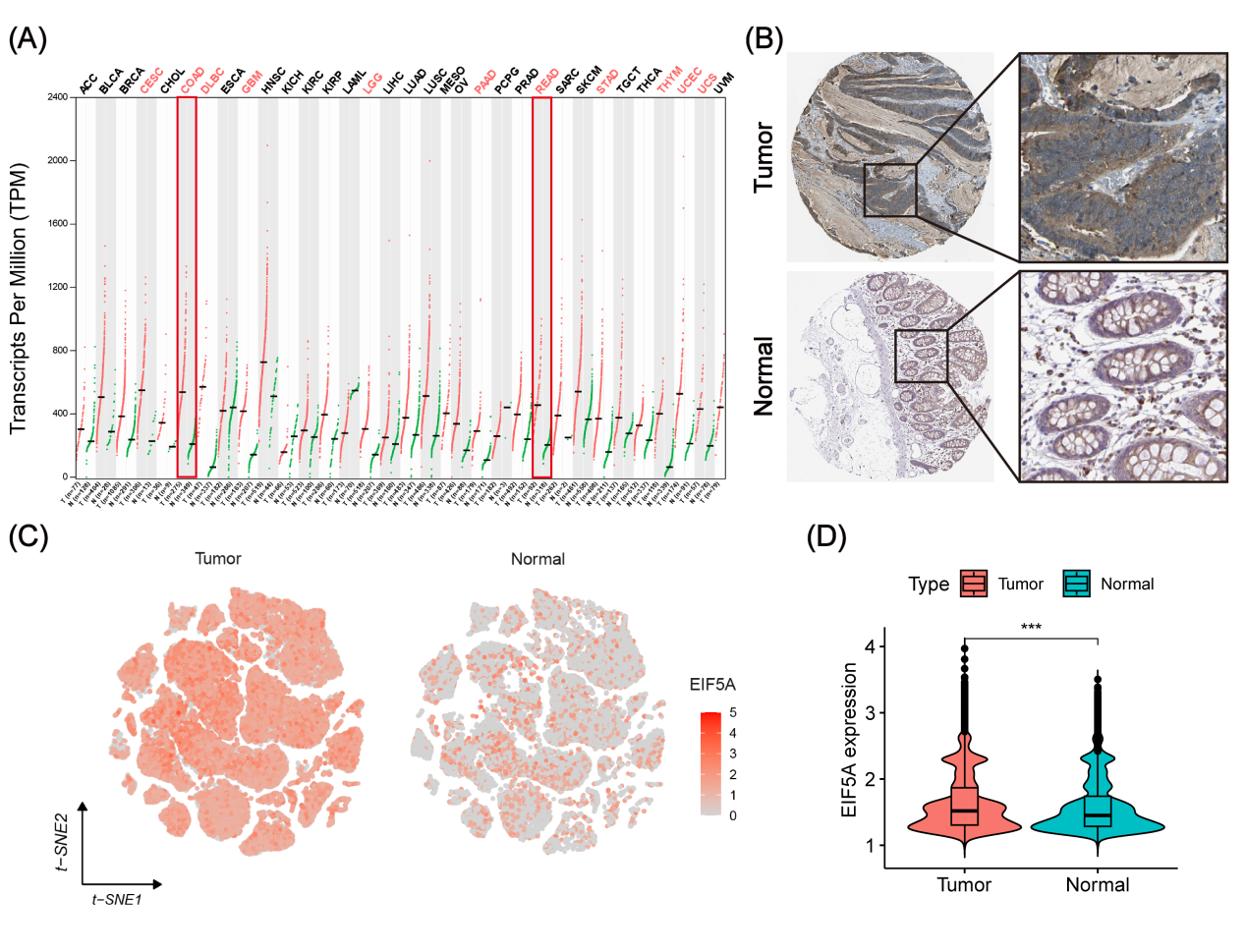


**Figure S1. The differential expression of EIF5A between tumors and normal/peritumors in TCGA and scRNA-seq cohorts. (A)** EIF5A expression profiles across tumor samples compared to peritumor samples for 33 TCGA tumor types using GEPIA. **(B)** Representative IHC images of EIF5A immunoreactivity in CRC tissue compared to normal liver tissue via HPA. **(C-D)** t-SNE plot and violin plot showing the distribution of EIF5A expression between tumor and peritumor samples. **TCGA:** The Cancer Genome Atlas; **scRNA-seq:** Single-cell RNA sequencing; **GEPIA:** Gene Expression Profiling Interactive Analysis; **IHC：**Immunohistochemistry; **CRC:** Colorectal cancer; **HPA:** The Human Protein Atlas; **t-SNE:** Stochastic neighbor embedding; *****:** P < 0.001.


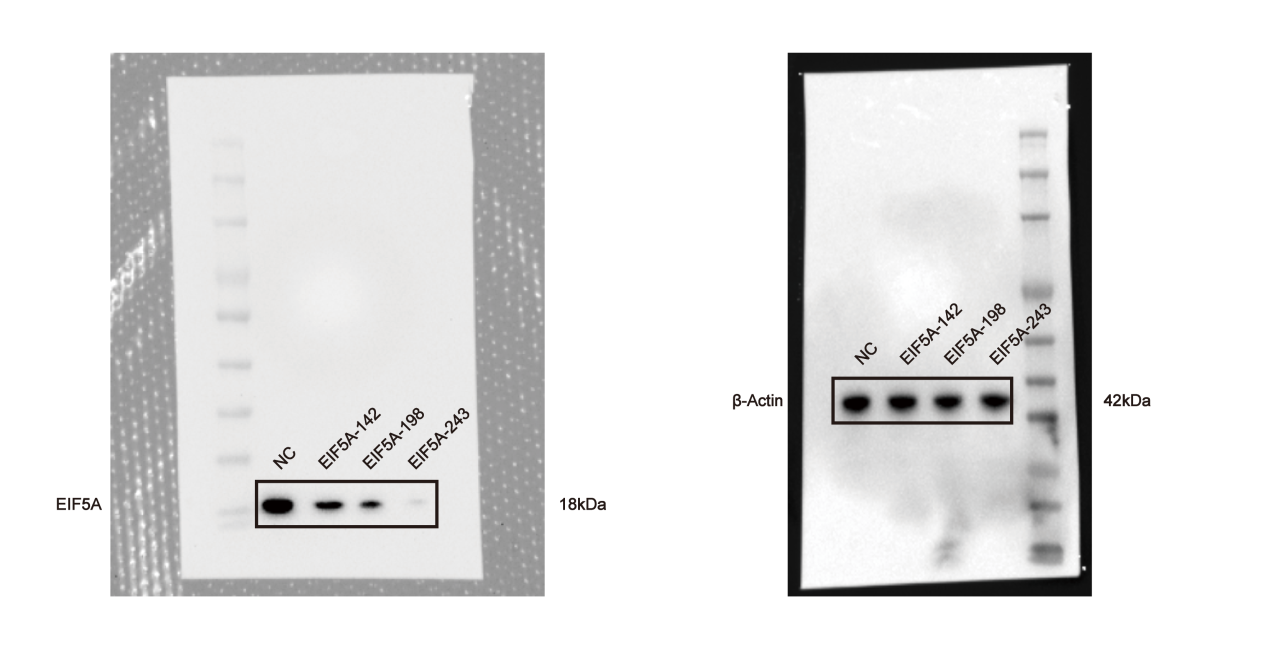


**Figure S2.** **The comprehensive scan of the unprocessed blots detailing the expression levels of EIF5A proteins after transfecting with siRNA, as visualized in Figure 4A. siRNA: small interfering RNA.**


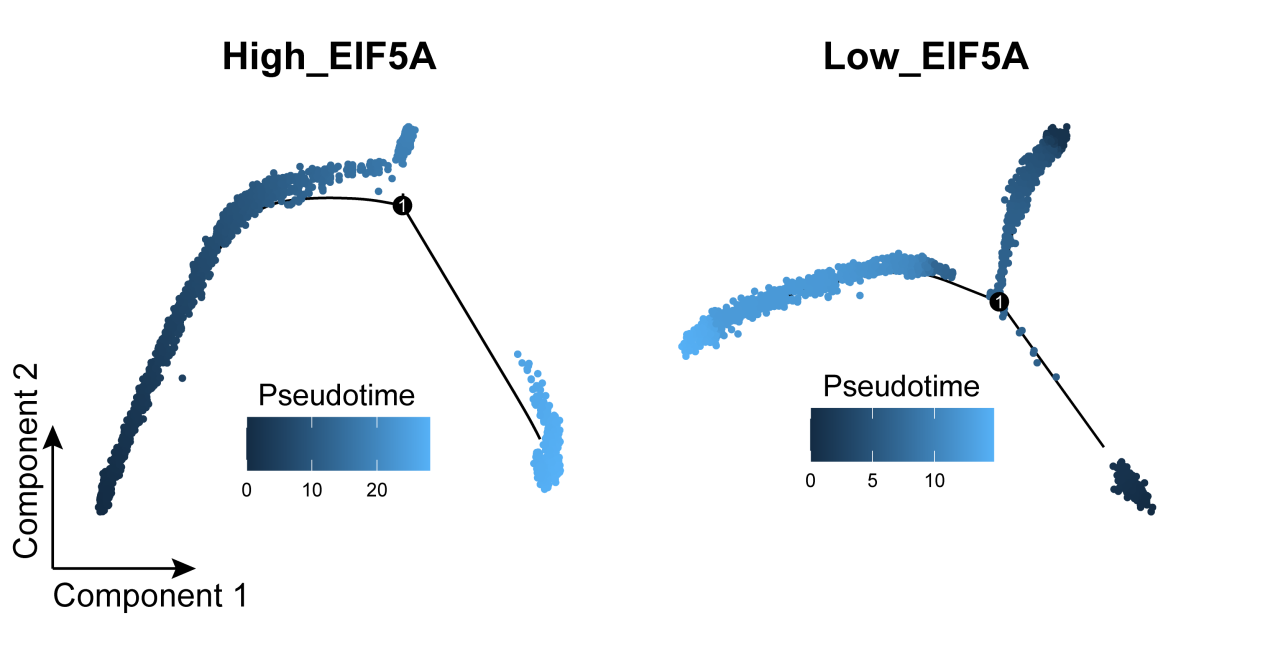


**Figure S3. Pseudotime of high- and low-EIF5A expression groups via pseudotime analysis.**


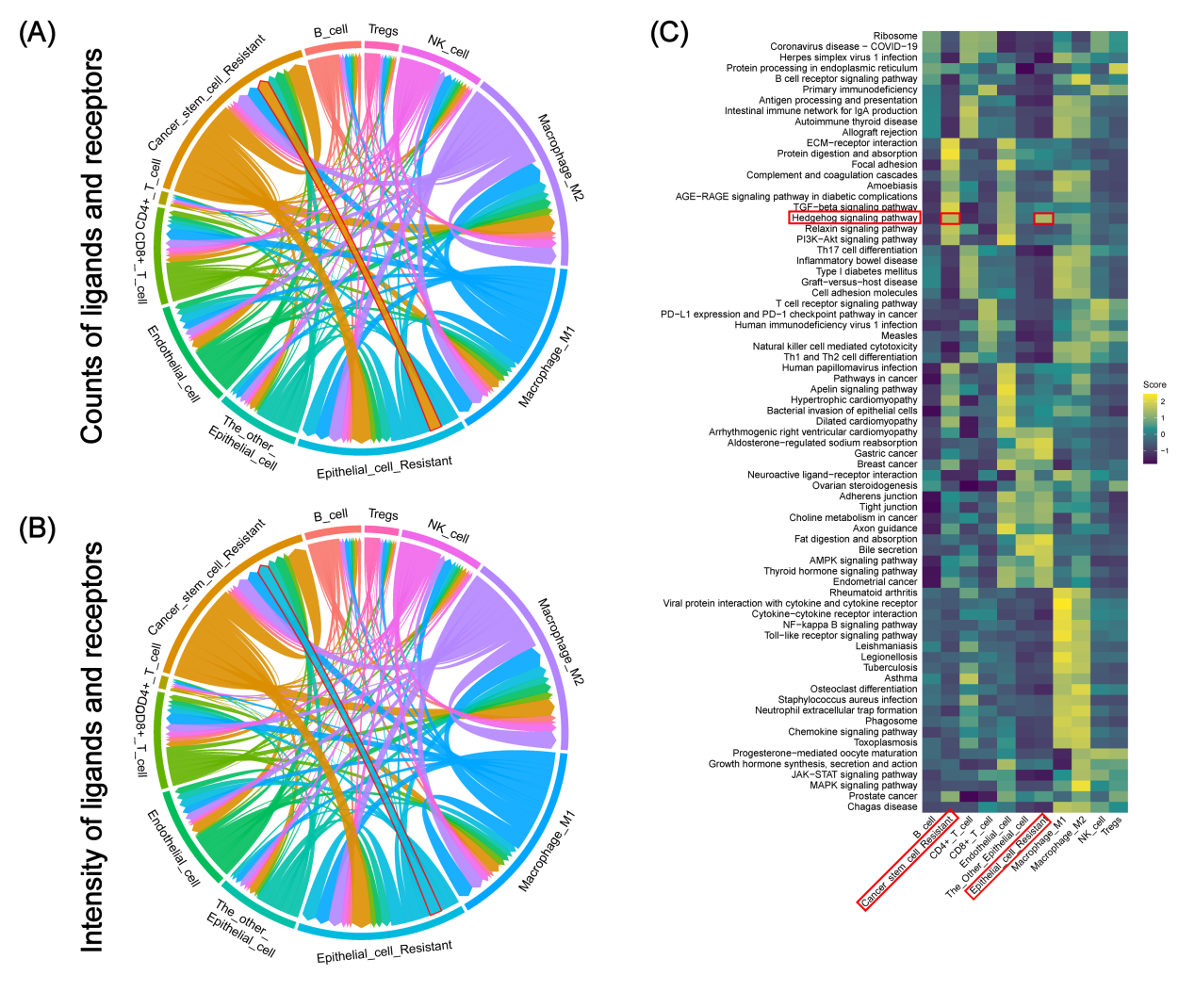


**Figure S4. Cellular communication and potential signaling pathways among cell subpopulations. (A)** Circos plots showing the interaction counts and interaction intensity of ligands and receptors among cell subpopulations. **(B)** Heatmap indicating the potential pathways activated in cell subpopulations.


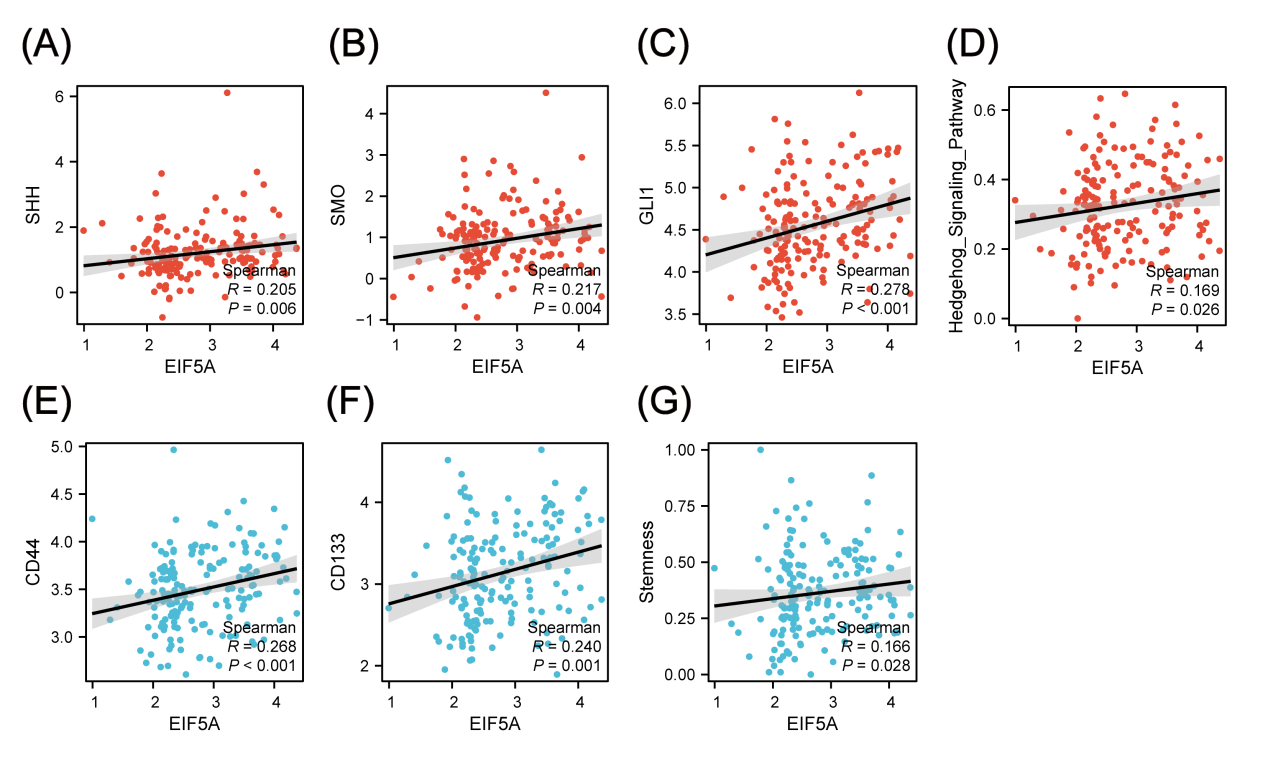


**Figure S5. The correlations between EIF5A expression and the Hedgehog signaling pathway and stemness. (A-D)** Scatter plot showing the correlations between EIF5A expression and the Hedgehog signaling pathway, the expression of SHH, SMO, and GLI1. **(E-G)** Scatter plot indicating the correlations between EIF5A expression and stemness, the expression of CD133 and CD44.


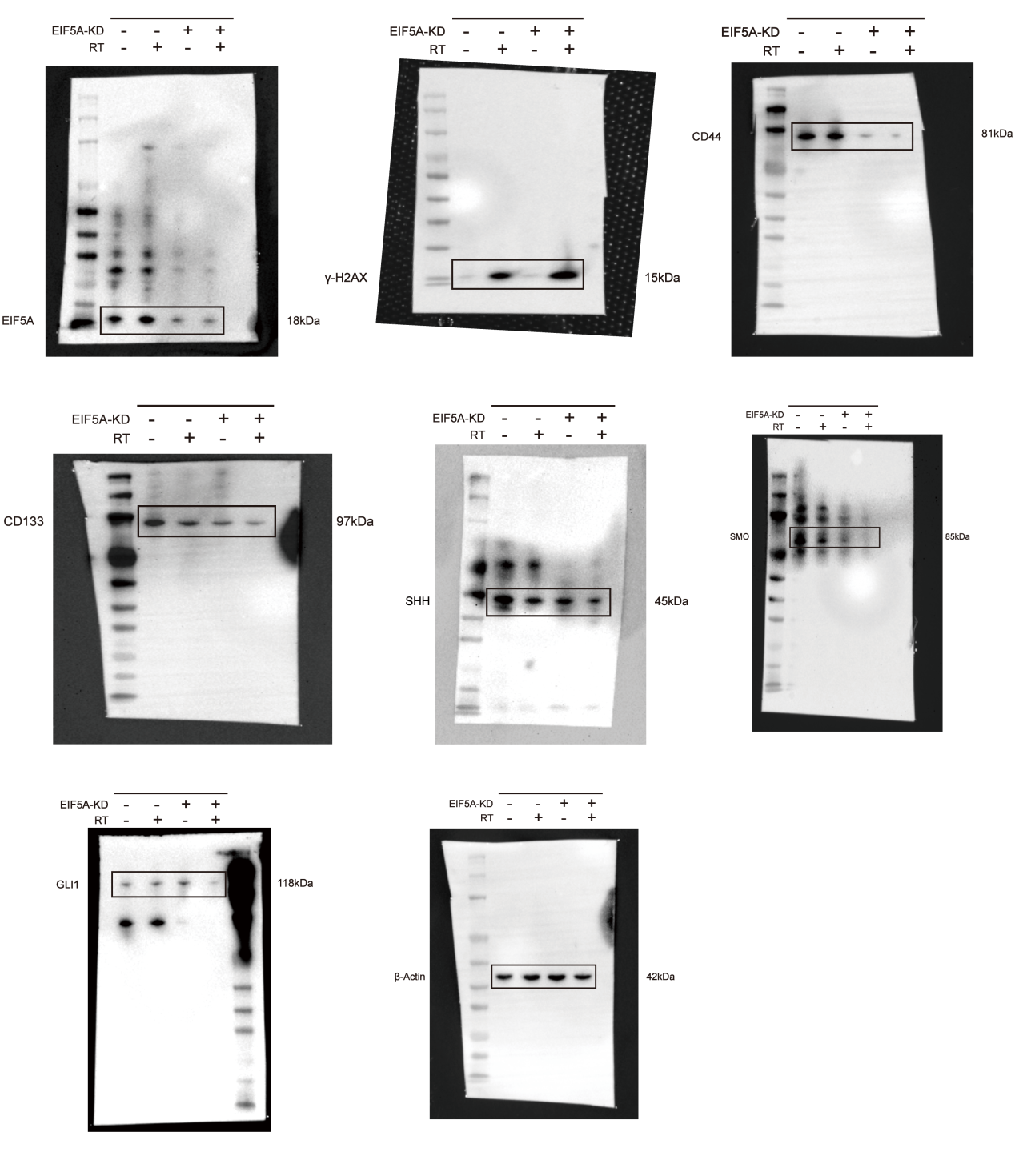


**Figure S6. The comprehensive scan of the unprocessed blots of the expression of GLI1, SMO, SHH, CD133 , CD44, and γ-H2AX, as visualized in Figure 6G.**


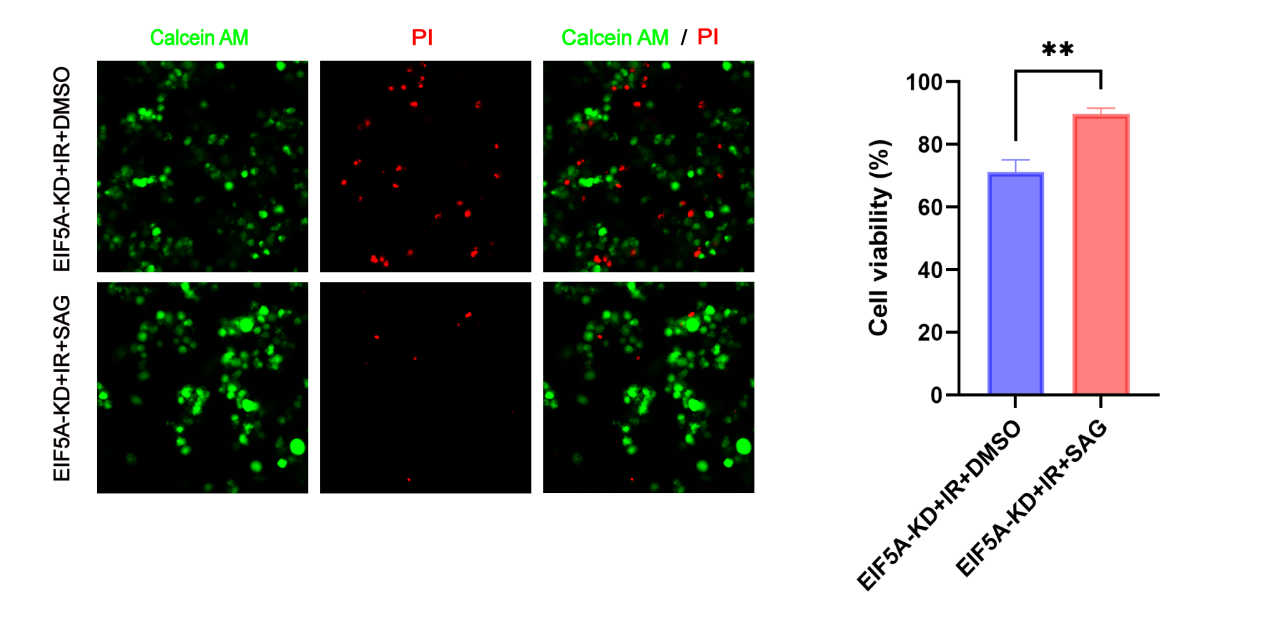
**Figure S7. Calcein-AM/PI staining assay to detect the cell death in irradiated EIF5A-KD CRC cells within the DMSO Group and the SAG (a Hedgehog pathway agonist) group. PI:** Propidium Iodide; **CRC:** Colorectal cancer; ****:** P < 0.01.


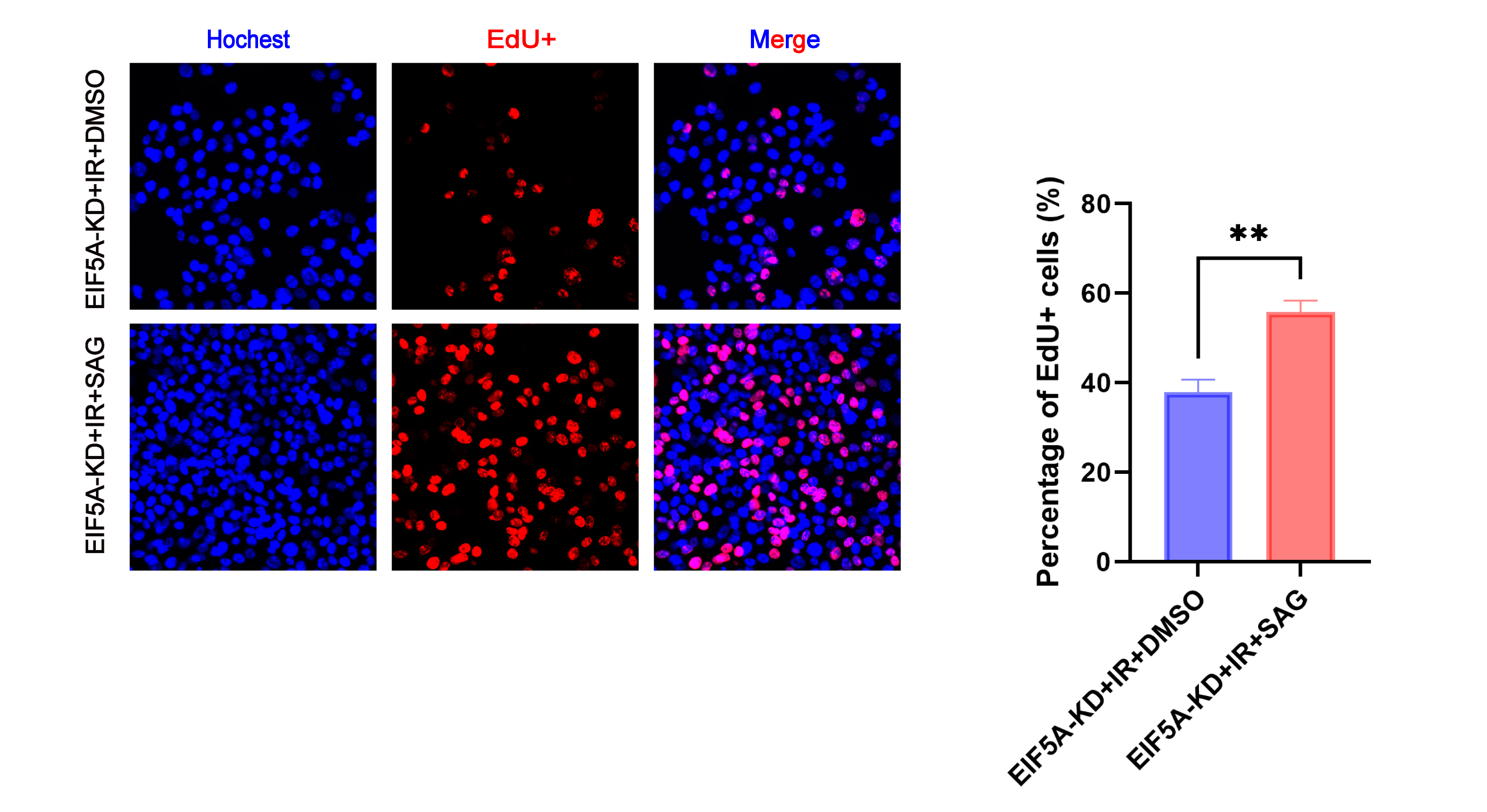


**Figure S8. EdU incorporation assay to detect the proliferation in irradiated EIF5A-KD CRC cells within the DMSO Group and the SAG (a Hedgehog pathway agonist) group. EdU:** 5-Ethynyl-2-Deoxyuridine; **CRC:** Colorectal cancer; ****:** P < 0.01.


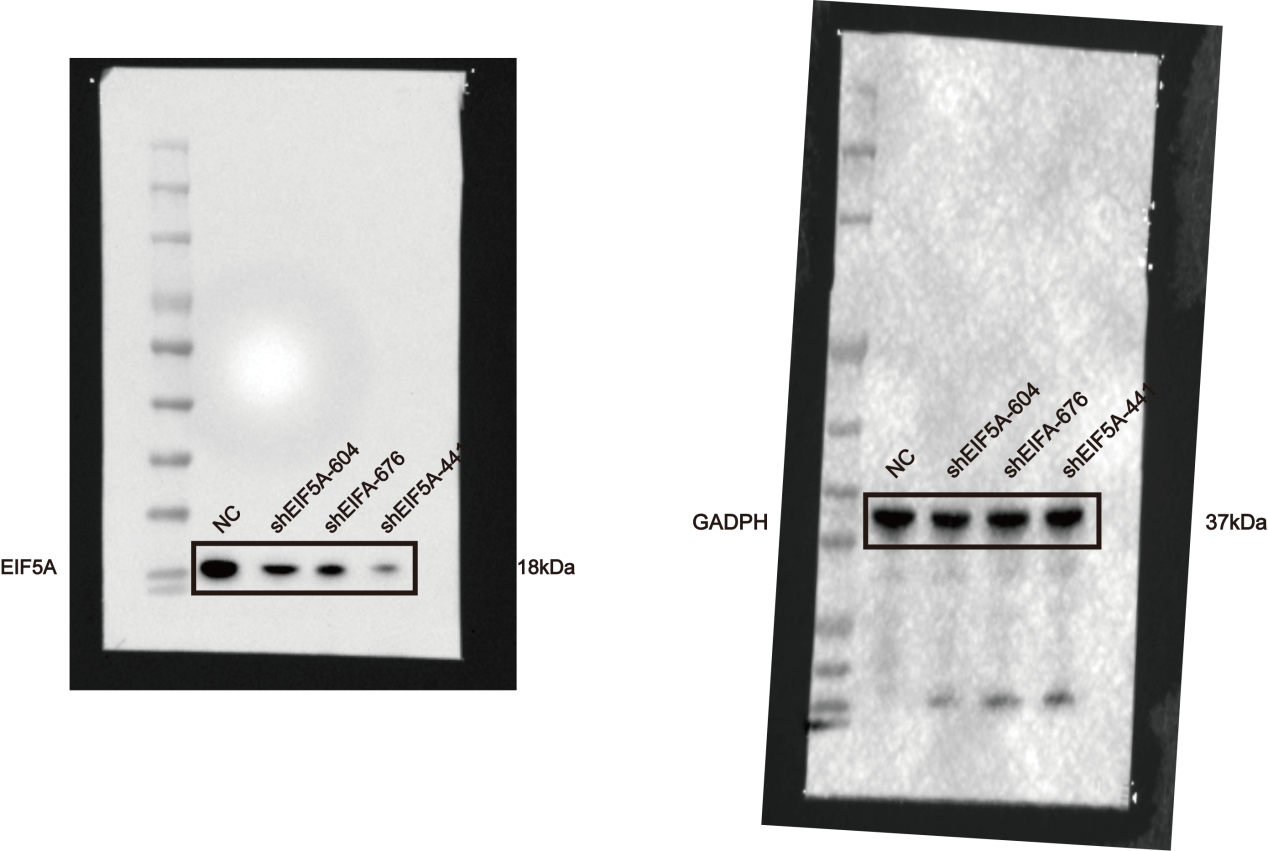


**Figure S9. The comprehensive scan of the unprocessed blots detailing the expression levels of EIF5A proteins after transfecting with shRNA, as visualized in Figure 7D. shRNA: short hairpin RNA.**


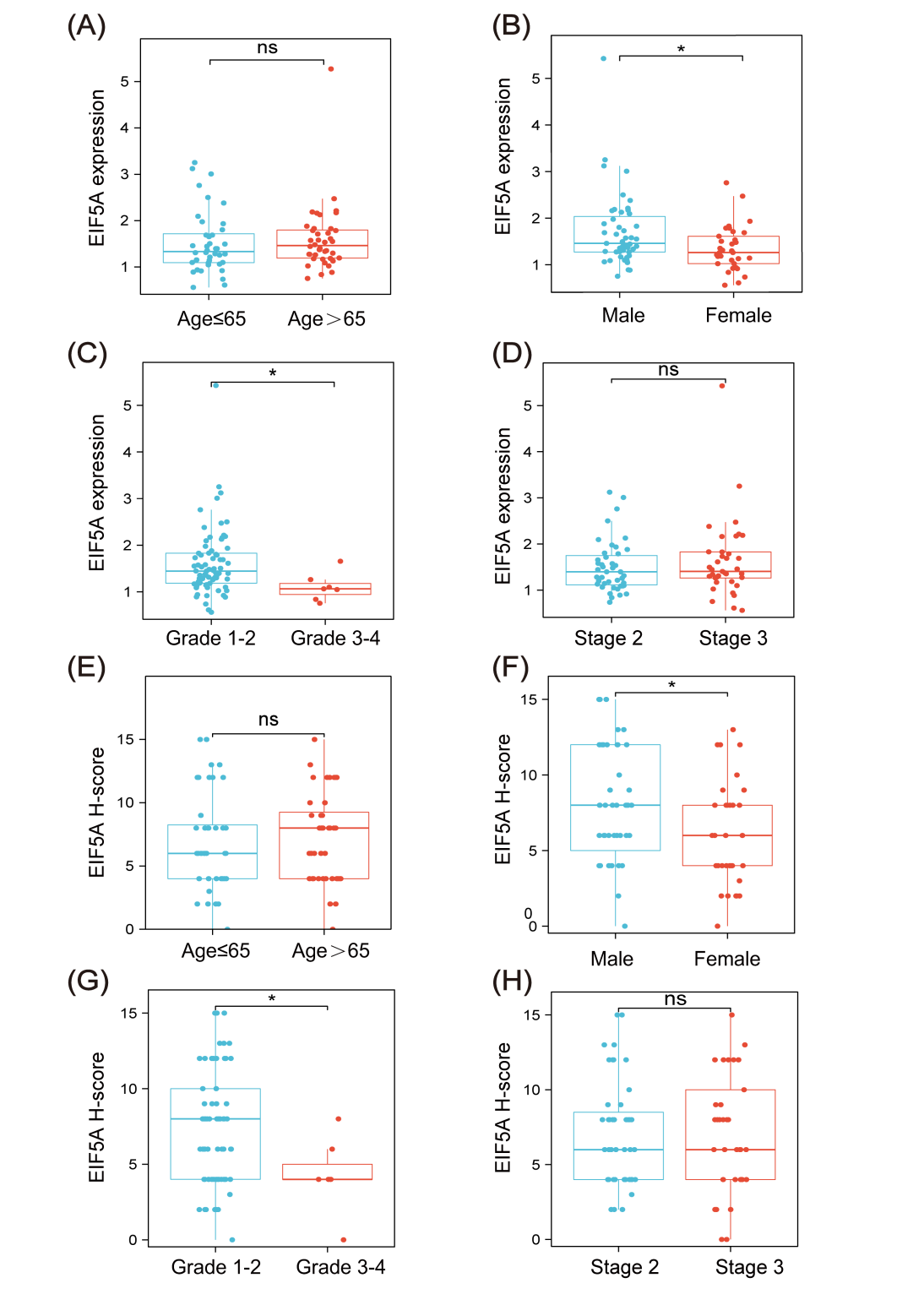


**Figure S10. Clinicopathological characteristics evaluation by EIF5A in in-house cohort. (A-D)**Boxplot showing the distribution of EIF5A expression between the groups of age, gender, grade and clinical stage.**(E-H)**Boxplot indicating the distribution of EIF5A H-score between the groups of age, gender, grade and clinical stage.**ns:** Non-statistics significance;***:** P < 0.05.


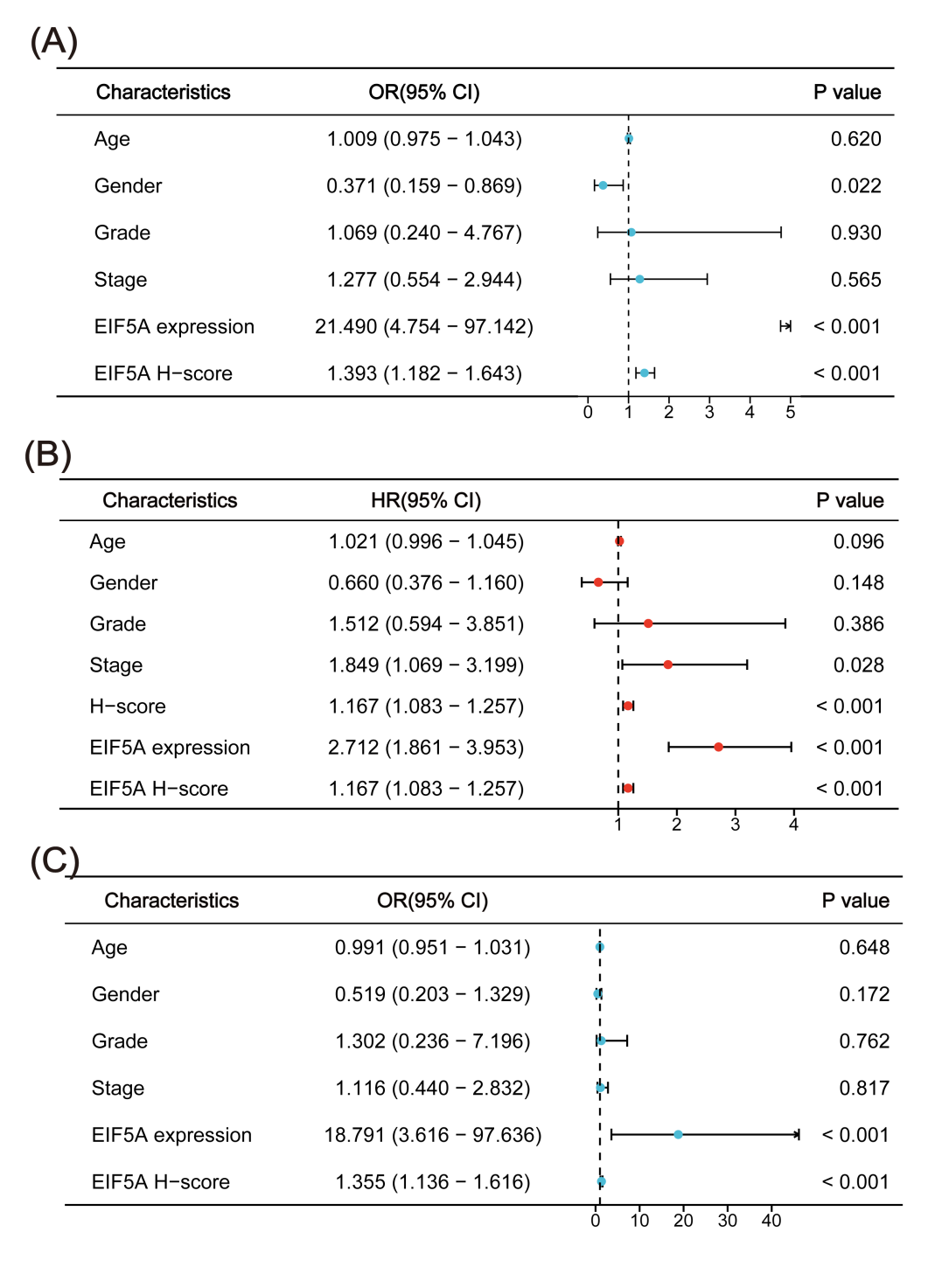


**Figure S11. Forest plots of logistic regression & univariate and multivariate COX regression analysis for EIF5A in in-house cohort. (A)** Logistic regression analysis of EIF5A expression & H-score for predicting radiotherapy sensitivity**. (B)** Univariate and multivariate Cox regression analysis of EIF5A expression & H-score for predicting the prognosis. **(C)** Logistic regression analysis of EIF5A expression & H-score for predicting CRC. **CRC:** Colorectal cancer;
